# A scoping literature review of global dengue age-stratified seroprevalence data: estimating dengue force of infection in endemic countries

**DOI:** 10.1016/j.ebiom.2024.105134

**Published:** 2024-05-07

**Authors:** Anna Vicco, Clare McCormack, Belen Pedrique, Isabela Ribeiro, Gathsaurie Neelika Malavige, Ilaria Dorigatti

**Affiliations:** a Department of Molecular Medicine, University of Padua, Padua, Italy; b MRC Centre for Global Infectious Disease Analysis, School of Public Health, Jameel Institute, Imperial College London, London, United Kingdom; c Drugs for Neglected Diseases Initiative (DNDi), Geneva, Switzerland

**Keywords:** Catalytic models, Antibodies, Age of first infection, Dengue transmission intensity, Endemic transmission

## Abstract

**Background:**

Dengue poses a significant burden worldwide, and a more comprehensive understanding of the heterogeneity in the intensity of dengue transmission within endemic countries is necessary to evaluate the potential impact of public health interventions.

**Methods:**

This scoping literature review aimed to update a previous study of dengue transmission intensity by collating global age-stratified dengue seroprevalence data published in the Medline, Embase and Web of Science databases from 2014 to 2023. These data were then utilised to calibrate catalytic models and estimate the force of infection (FOI), which is the yearly per-capita risk of infection for a typical susceptible individual.

**Findings:**

We found a total of 66 new publications containing 219 age-stratified seroprevalence datasets across 30 endemic countries. Together with the previously available average FOI estimates, there are now more than 250 dengue average FOI estimates obtained from seroprevalence studies from across the world.

**Interpretation:**

The results show large heterogeneities in average dengue FOI both across and within countries. These new estimates can be used to inform ongoing modelling efforts to improve our understanding of the drivers of the heterogeneity in dengue transmission globally, which in turn can help inform the optimal implementation of public health interventions.

**Funding:**

UK Medical Research Council, Wellcome Trust, Community Jameel, Drugs for Neglected Disease initiative (DNDi) funded by the French Development Agency, Médecins Sans Frontières International; Swiss Agency for Development and Cooperation and UK aid.

**Research in context**

**Evidence before this study**

With half of the world's population at risk of infection, dengue poses a significant public health challenge worldwide. However, its transmission intensity in several endemic countries remains poorly understood, making it challenging to assess the burden of disease and the potential impact of control interventions.

**Added value of this study**

In this work, we conducted a scoping literature review to gain a more comprehensive understanding of the heterogeneity in dengue transmission intensity within endemic countries. We collated global dengue age-specific seroprevalence data from endemic areas published between 2014 and 2023 in Medline, Embase and Web of Science. These data were used to calibrate mathematical models and estimate the average yearly force of infection (FOI), which is a fundamental measure of transmission intensity.

**Implications of all the available evidence**

We found a total of 66 new publications with 219 relevant datasets from 30 dengue endemic countries and estimated the dengue FOI at the finest available spatial resolution. The FOI estimates published in this study quantify the average risk of infection, characterise its spatial heterogeneities, and can be used to estimate dengue infection and disease burden as well as the potential impact of new interventions, such as vaccination, thus contributing to ongoing efforts to better characterise and map dengue transmission intensity and burden worldwide.

## Introduction

Dengue is a rapidly spreading mosquito-borne viral infection transmitted to humans by *Aedes* mosquitoes, primarily affecting tropical and sub-tropical regions.^1^ With half of the world's population at risk of contracting the disease, dengue is the leading arboviral disease among humans. However, its transmission dynamics in several countries remain poorly understood.^2^

Notifications of suspected or virologically confirmed dengue cases, together with information on the age and location of the reported cases, provide the necessary information to reconstruct the immunity profile (i.e., the age-dependent susceptibility to infection) of local populations. This, in turn, can be used to estimate the transmission intensity of dengue, as defined by the force of infection (FOI), which is the per-capita rate of infection for a susceptible individual. However, while routinely collected case-notification data exists for several locations in South East Asia and Latin America, case reporting in Africa is more variable and not mandatory due to the lack of point-of-care diagnostics and more limited testing capacity– with cases mainly reported in sporadic outbreaks or in individual case reports—which has hindered arbovirus surveillance across the continent.^3^

Serological surveillance involves the collection and testing of blood samples for the presence of IgG antibodies against dengue virus using qualitative or quantitative serological assays. This approach allows for the detection of previous infections, regardless of the level of symptoms experienced during infection.^4^ This is particularly important for diseases such as dengue, where asymptomatic infections are very frequent^5^ and antibody levels against the infecting serotype are long-lived. By providing information on past exposure, serological surveillance is particularly useful for gaining insight into the historical circulation of dengue and for reconstructing the immunity profile of a population. This, in turn, can be used to inform surveillance, preparedness planning, the optimal implementation of existing and new vector control strategies, such as the release of Wolbachia-carrying mosquitoes,^6^ and vaccination strategies^7^^,^^8^ and to assess their potential impact.

Several different approaches have been taken to map the risk of dengue infection in the current and changing climate,^9^ using multiple risk metrics including environmental, climate and habitat suitability,^2^^,^^10^^,^^11^ R0 estimates^12^ and occurrence data.^13^ While more limited in number than occurrence data, FOI estimates have the advantage of allowing us to estimate changes in the burden of infection and disease in the presence of large-scale interventions, such as age-targeted vaccination campaigns in endemic settings, where accounting for pre-existing immunity is important.^14^ The first global map of dengue FOI was developed by Cattarino et al.,^15^ where machine learning was used to link 382 geolocated dengue force of infection (FOI) estimates with a set of ecological and demographic variables. Dengue FOI predictions were then imputed globally, according to underlying climatic, environmental and demographic conditions, including in places with no (serological nor case-based) dengue surveillance. Ongoing efforts aiming to validate and refine the FOI projections and burden estimates obtained by Cattarino et al.^15^ require an update of the earlier literature review conducted by Imai et al., in 2014,^4^ which provided the serologically-derived FOI estimates used by Cattarino et al.^15^ for the model calibration.

In this work, a literature search was conducted to collect global dengue age-stratified seroprevalence data published between 2014 and the end of 2023 to update our current understanding of dengue transmission intensity in endemic countries. As in Imai et al.,^4^ these data have been used to calibrate catalytic models to estimate setting-specific dengue FOI which help characterise the heterogeneity and underlying drivers of dengue transmission and will inform preparedness and response planning going forward.

## Methods

### Literature search

We searched Medline, Embase and Web of Science for publications reporting age-stratified dengue seroprevalence datasets published between January 2014 and October 2023 to complement the literature search previously conducted by Imai et al.^4^ We used a Boolean search query with search terms:

Dengue and sero∗ and (prevalence or seroprevalence or positiv∗ or seropositiv∗)

The specific search terms for each database are reported in the Supplementary data (Tables S1 and S2). After removing duplicate articles, articles in languages other than English, and non-article publications (e.g., conference posters and book chapters), we retained all remaining papers for evaluation of their potential relevance according to the titles and abstracts. The selected papers were evaluated for full-text eligibility, with the aim of collating data on age-specific dengue seroprevalence studies conducted in the general population in specific, geolocated endemic settings. Following Brady et al.,^16^ we defined endemic countries as those with “good” or “complete presence” of dengue.

Given our focus on estimating the susceptibility profile of the population and the FOI, we excluded papers where only the overall seroprevalence (i.e., not stratified by age) was reported, or where the study focused on hospital cases, clinical trials of antivirals, vaccine studies, or reported a secondary analysis of data published elsewhere (in which case, we included the original article presenting the primary data when the publication date fell within the search criteria). From the selected papers, we recorded the country and specific location of the survey (when available), the age range of the subjects, the number of age groups tested and the respective survey sizes, as well as the date of the survey and the type of assay used.

### Estimating the force of infection

We used catalytic models and a Bayesian inferential approach to estimate the dengue force of infection *λ* from serological data, as done by Imai et al.^4^ We used a simple catalytic model (model A), which assumes a constant infection hazard *λ* and no waning of immunity, to fit most cross-sectional serological (IgG) datasets, with the proportion seropositive subjects in age group *a_i_* (z(*a_i_*)) given by Eq (1),

(1) $$z\left(a_{i}\right)=1\;-\;e^{-\lambda {\;a}_{i}}$$

where *i* denotes the age group and *a*_*i*_ the mid-age of the age group. Whenever the data showed evidence of declining seroprevalence with age, we used model B, which is a modified version of model A with an extra parameter *α*, representing the antibody decay rate to account for antibody waning. Assuming a constant force of infection *λ* and decay rate *α*, the seroprevalence for model B is given by Eq (2).

(2) $$z\left(a_{i}\right)=\frac{\lambda }{\lambda \;+\;\alpha }\left[1-\;e^{{–\;a}_{i}\left(\lambda +\alpha \right)}\right]$$

We fitted two versions of models A and B to the age-stratified serological data, assuming a binomial (models A1 and B1) and beta-binomial (models A2 and B2) distribution of the observed age-stratified seroprevalence, using the Hamiltonian Monte-Carlo algorithm implemented in the *CmdStanR*.^17^^,^^18^

In models A2 and B2, we accounted for overdispersion of the data through an over–dispersion parameter *γ*, which was separately estimated for each spatial location as described by Imai et al.^4^

For the datasets where we only had information on the percentage of people testing positive, and not on the total number of tested people, we fitted the data using model C, where we assumed that the age-stratified seroprevalence ($X_{a_{i}}$) was normally distributed with mean ($\mu_{a_{i}}$ = *z* (*a*_*i*_)) and variance ($\sigma^{2}$) to be estimated, as described by Eq (3).

(3) $$X_{a_{i}}\;∼\;\text{Normal}\left(\mu_{a_{i}},\sigma^{2}\right)$$

For the analysis of PRNT (Plaque Reduction Neutralization Test) results, since we only had information on the percentage of seropositive subjects per serotype, we used a modified version of model C, named model D, where we assumed a serotype-specific average FOI ${\lambda \;}_{j}$ and that the mean of the seroprevalence shown in Eq (3) was serotype specific, as shown in Eq. (4).

(4) $$X_{a_{i},j}∼\text{Normal}\left(\mu_{a_{i},j},\sigma^{2}\right)\;with\;j=1,\ldots ,4\text{,}$$

We estimated the average force of infection across the four serotypes *λ* as the average of the serotype-specific mean FOIs ${\lambda \;}_{j}$. We assumed informative prior distributions on all estimated parameters. The prior distributions of the FOI λ and antibody decay rate α were defined as normal distribution with mean 0.1 and standard deviation 0.1, while for the variance $\sigma^{2}$, we assumed a normal prior distribution with mean 0 and standard deviation 1, and for the over–dispersion parameter *γ* we assumed an exponential prior distribution centred in 1. We set a lower bound of 0 for all parameters.

For each dataset we reported the average age of first infection, calculated as 1/λ, and the age at which we expect x seroprevalence (with x = 50% and 70%), calculated as −ln(1−x)/λ for model A, C and D and as $-\ln\left(1-x\frac{\lambda +\alpha }{\lambda })/(\lambda +\alpha \right)$ for model B.

### Statistics

We used Rhatt, n_eff_ and visual inspection of the trace plots to assess convergence.^17^ The deviance information criterion (DIC) was used for model selection. The DIC is defined as $\text{DIC}=2\text{ll}\left(\bar{\theta }\right)-4\bar{\text{ll}\left(\theta \right)}$, where and $\text{ll}\left(\bar{\theta }\right)$ and $\bar{\text{ll}\left(\theta \right)}$ respectively denote the loglikelihood computed at the mean of the parameters and the mean loglikelihood. Models with the lowest DIC are preferred, and differences in DIC of at least 4 units are typically used for model selection.^19^ We calculated the median and 95% credible interval (CrI) for each estimated parameter. Uncertainty around the modelled seroprevalence, the average age of first infection and the age of 50% and 70% seroprevalence were estimated by extracting 100 random values from the posterior distribution of the parameters and reporting the median and 95% CrI (2.5 and 97.5 percentiles) of the modelled statistic. Each observed seroprevalence data point was presented as mean and the 95% binomial confidence interval (CI) calculated with the Agresti-Coull method.^20^

### Ethics

We used publicly available data published in the papers identified in the literature review.

### Role of funders

The funders had no role in the study design, data collection, data analyses, interpretation or writing of report; the findings and conclusions contained herein are those of the authors and do not necessarily reflect positions or policies of the aforementioned funding bodies.

## Results

### Article selection and dataset characteristics

**Fig. 1:**
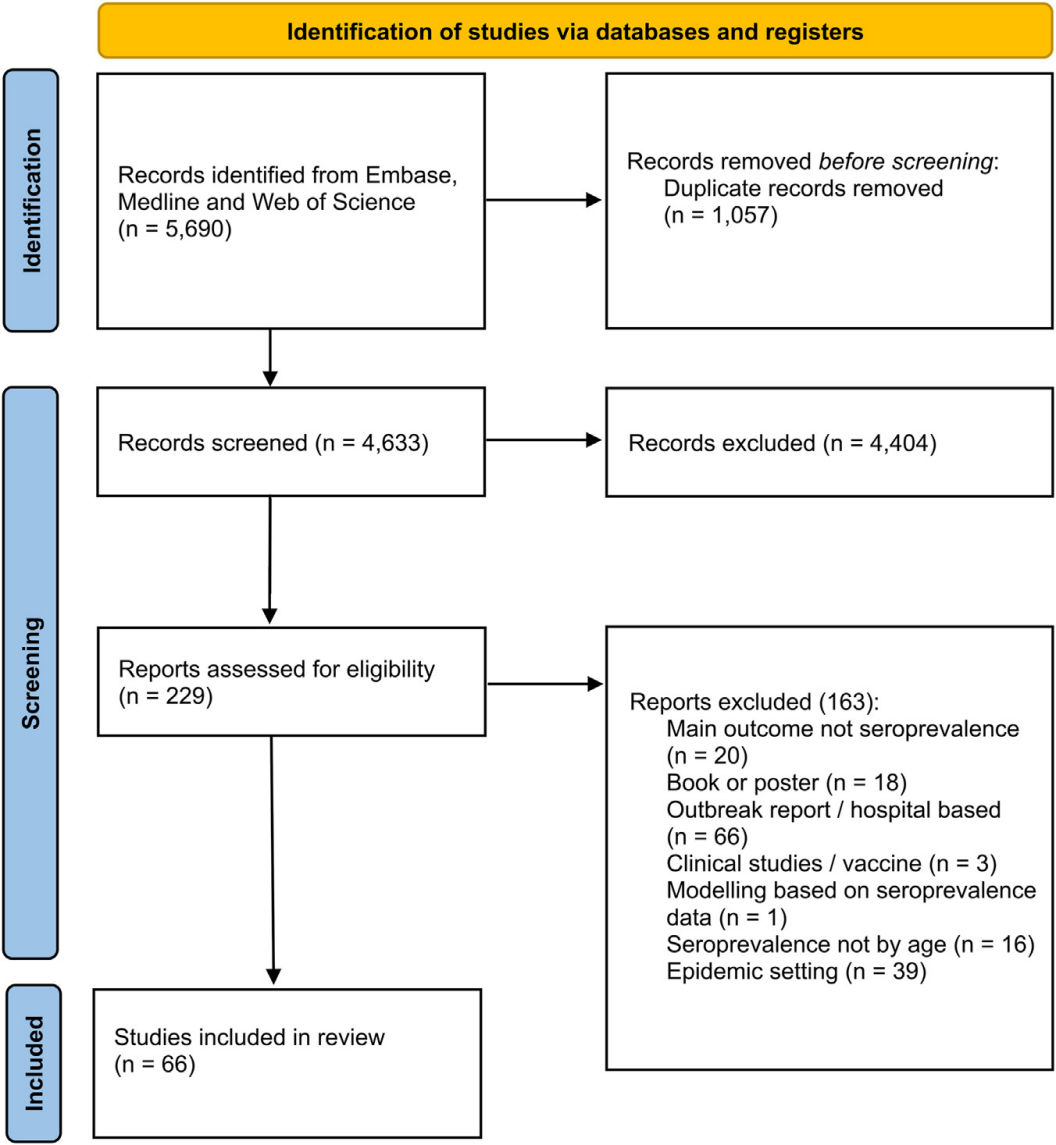
**PRISMA chart of the literature search**. Literature review on dengue age-stratified seroprevalence studies published from 2014 to the end of October 2023 in Embase, Medline and Web of Science using the word search “dengue AND sero∗ AND (prevalence OR seroprevalence OR positiv∗ OR seropositiv∗).

**Fig. 2:**
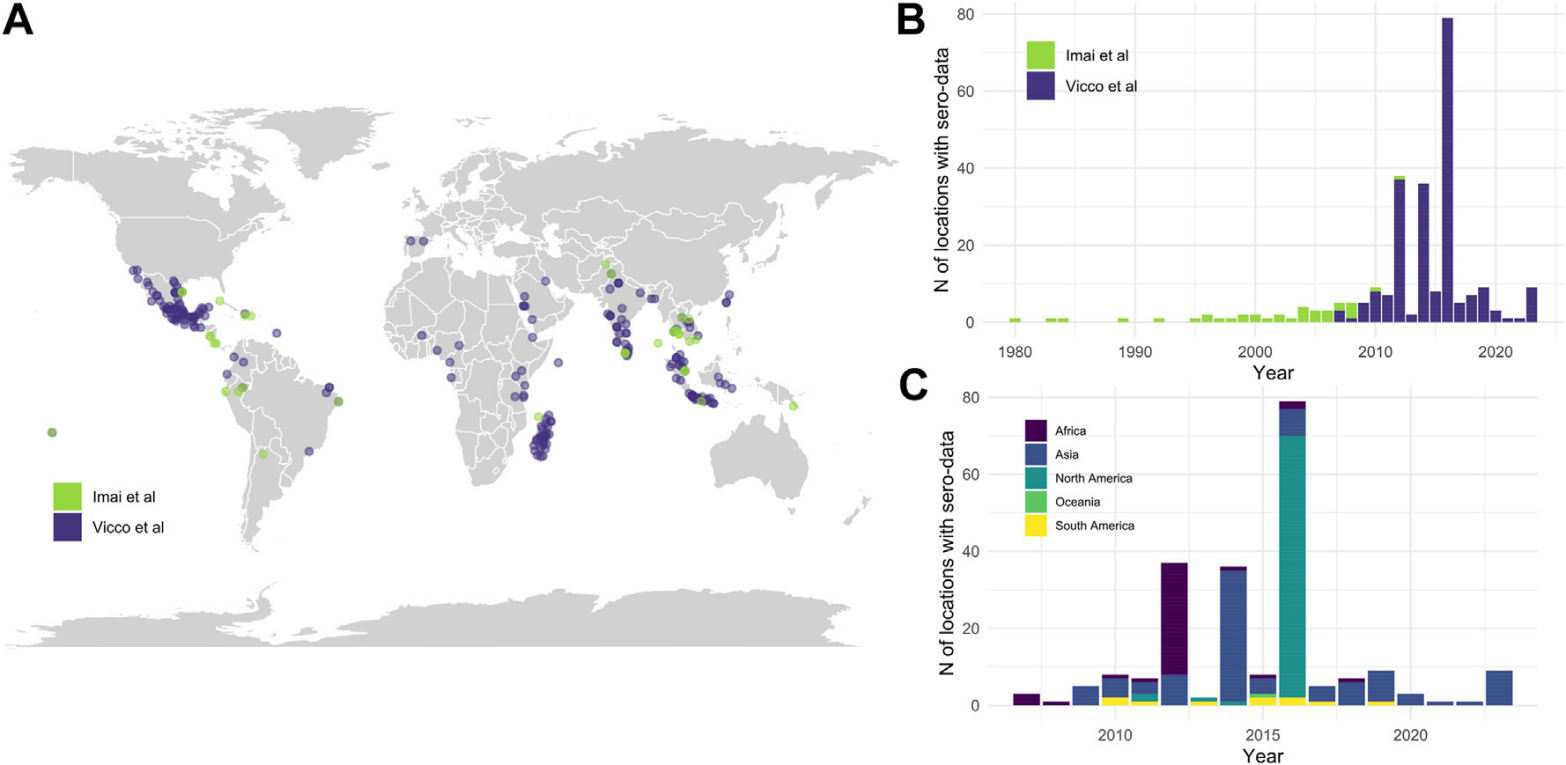
**Location and year of published dengue seroprevalence studies**. Panel A: location of published age-stratified dengue seroprevalence data, as identified in Imai et al.^4^ (green) and in this literature review (purple). Panel B: number of age-stratified dengue serosurveys per year as identified in Imai et al.^4^ (green) and in this literature review (purple) by year of the serosurvey. Panel C: number of age-stratified dengue serosurveys by serosurvey year and continent (in colour).

We found a total of 5690 potentially relevant articles. Once we removed duplicates, 4633 papers were retained for evaluation of titles and abstracts. Of these, 4404 articles were found to be irrelevant for the purpose of the study and were therefore excluded. The remaining 229 papers were evaluated for full-text eligibility, from which we identified 66 studies reporting age-specific seroprevalence datasets (Fig. 1) from surveys conducted between 2006 and 2023 in various dengue endemic countries and published after 2014 (Fig. 2).

This literature review identified 219 new dengue FOIs from serological survey, more than five times the number published in Imai et al.,^4^ which indicates an increased interest in using serology for dengue surveillance worldwide in the last decade (Fig. 2).

Table 1 reports the estimated yearly FOI together with the main characteristics of each serosurvey, including the country, location, age range, number of age groups tested and size of the survey, the type of assay used and relative commercial name, and the date when the survey was performed. IgG ELISA was the assay most frequently used. Out of 66 studies, 17 focused on children and young people (up to 22 years old), mainly tested in schools and universities.

**Table 1:** Summary of age-specific seroprevalence datasets identified in this literature review and location-specific yearly FOI estimates provided as median and 95% CrI.

Country	Location	Age range (years)	Age groups	Total tested	Type of test	Reference	Study date	Model	FOI (95% CrI)	Commercial name
Bangladesh	blood service	18–67	5	507	IgG ELISA	unpublished Hoque et al.^21^	Jan–March 2022	B	0.133 (0.038, 0.288)	SD Bioline Dengue DUO IgM/IgG test
Bangladesh	Dhaka city	0–67+	7	1126	IgG ELISA	Dhar-Chowdhury et al.^22^	2012	A	0.045 (0.026, 0.071)	in-house
Brazil	Sau Paulo	10–91	4	1322	IgG ELISA	Chiaravallotti et al.^23^	Oct 2015–Mar 2016	A	0.030 (0.013, 0.055)	anti-dengue IgG elisa kit (Abcam, Cambridge, UK)
Brazil	Fortaleza	05–13	9	2117	IgG capture ELISA	CBC et al.^24^	Sep 2011–Jan 2015	C	0.096 (0.080, 0.115)	IgG capture ELISA (Panbio, Brisbane, Australia)
Brazil	Fortaleza	02–12	2	483	indirect IgG ELISA	Zhang et al.^25^	Nov 2019–Feb 2020	A	0.086 (0.024, 0.215)	Panbio Dengue IgG nidirect ELISA
Brazil	Recife	05–65	6	2070	IgG for NS1 ELISA	Braga et al.^26^	Aug 2018–Feb 2019	A	0.052 (0.028, 0.080)	in-house
Brazil	Tapera of Itarema (municipality) in state of Ceara	0–59+	3	288	indirect IgG ELISA	Sacramento et al.^27^	Oct–Nov 2015	A	0.009 (0.007, 0.011)	dengue IgG indirect ELISA (Panbio, Australia)
Burkina faso	Ougadougou	01–55	8	2897	indirect IgG ELISA	Lim et al.^28^	Jun 2015–Mar 2017	A	0.063 (0.059, 0.066)	Panbio dengue IgG indirect elisa (Abbott)
Cameroon	Doualaa	02–45+	5	666	IgG ELISA	Demanou et al.^29^	2006–2007	A	0.034 (0.030, 0.038)	in-house (IMTSSA, Marseille, France)
Cameroon	Garoua	02–45+	5	710	IgG ELISA	Demanou et al.^29^	2006–2007	A	0.009 (0.008, 0.011)	in-house (IMTSSA, Marseille, France)
Colombia	Anapoima, Apulo, Buenaventura, Quibdo, Tumaco, Tierralta	04–95	4	1318	indirect IgG ELISA	Vaelandia-Romero et al.^30^	Apr 2013–Oct 2015	A	0.047 (0.020, 0.088)	Panbio Alere test
Colombia	Quobdo	0-45+	4	58	capture IgG ELISA	Castellanos et al.^31^	2015∗	A	0.041 (0.028, 0.060)	capture UltraMicro-ELISA (Tecnosuma, La Havana)
Djibouti	Djibouti	0–60+	4	1035	indirect IgG ELISA	Andayi et al.^32^	Nov 2010–Feb 2011	A	0.008 (0.007, 0.009)	PanBio, Brisbane, Australia
Ecuador	Town of Quininde	0–60	11	319	IgG ELISA	Ster et al.^33^	Oct 2015–Feb 2016	A	0.022 (0.019, 0.027)	Abcam, Cambridge, UK
France caribbean	Guadeloupe and Martinique	18–70	7	783	IgG ELISA	L'Azou et al.^34^	2011	A	0.078 (0.070, 0.087)	unspecified
French guiana	national	02–65+	7	2697	MIA testing	Bailly et al.^35^	Jun–Oct 2017	C	0.069 (0.042, 0.173)	unspecified
French polynesia	Tahiti	0–16	11	476	IgG elisa	Nemoto et al.^36^	2014	C	0.091 (0.077, 0.108)	unspecified
Gabon	Lambarene	01–56	4	387	indirect IgG ELISA	Ushijima et al.^37^	Nov 2014–Jan 2017	A	0.028 (0.009, 0.078)	in-house
Haiti	Gressier, Jacmel and Chabin	0–50+	8	673	indirect IgG ELISA	Weppelmann et al.^38^	Feb–May 2013	B	0.209 (0.100, 0.352)	in-house
India	Delhi	05–10	6	967	indirect IgG ELISA	Garg et al.^39^	Jan 2011–Oct 2012	A	0.136 (0.125, 0.148)	Focus Diagnostic, California USA and Panbio Diagnostics, Brisbane, Australia
India	Kalayani	05–10	6	1556	indirect IgG ELISA	Garg et al.^39^	Jan 2011–Oct 2012	A	0.035 (0.032, 0.039)	Focus Diagnostic, California USA and Panbio Diagnostics, Brisbane, Australia
India	Wardha	05–10	6	500	indirect IgG ELISA	Garg et al.^39^	Jan 2011–Oct 2012	A	0.144 (0.085, 0.217)	Focus Diagnostic, California USA and Panbio Diagnostics, Brisbane, Australia
India	Mumbai	05–10	6	388	indirect IgG ELISA	Garg et al.^39^	Jan 2011–Oct 2012	A	0.220 (0.193, 0.249)	Focus Diagnostic, California USA and Panbio Diagnostics, Brisbane, Australia
India	Hyderabad	05–10	6	1177	indirect IgG ELISA	Garg et al.^39^	Jan 2011–Oct 2012	A	0.116 (0.107, 0.126)	Focus Diagnostic, California USA and Panbio Diagnostics, Brisbane, Australia
India	Bangalore	05–10	6	515	indirect IgG ELISA	Garg et al.^39^	Jan 2011–Oct 2012	A	0.141 (0.126, 0.158)	Focus Diagnostic, California USA and Panbio Diagnostics, Brisbane, Australia
India	North (Delhi, Punjab and Uttar Pradesh)	05–45	3	2402	indirect IgG ELISA	Murhekar et al.^40^	Jun 2017–Apr 2018	B	0.058 (0.022, 0.124)	Panbio, Standard diagnostics, Yongin-si, South Korea
India	North-East (Tripura, Meghalaya and Assam)	05–46	3	2360	indirect IgG ELISA	Murhekar et al.^40^	Jun 2017–Apr 2018	A	0.002 (0.002, 0.003)	Panbio, Standard diagnostics, Yongin-si, South Korea
India	East (Bihar, West Bengal and Odisha)	05–47	3	2486	indirect IgG ELISA	Murhekar et al.^40^	Jun 2017–Apr 2018	A	0.008 (0.007, 0.009)	Panbio, Standard diagnostics, Yongin-si, South Korea
India	West (Rajasthan, Madhya Pradesh and Maharashtra)	05–48	3	2336	indirect IgG ELISA	Murhekar et al.^40^	Jun 2017–Apr 2018	A	0.048 (0.045, 0.051)	Panbio, Standard diagnostics, Yongin-si, South Korea
India	South (Andhra Pradesh, Karnataka and Tamil Nadu)	05–49	3	2716	indirect IgG ELISA	Murhekar et al.^40^	Jun 2017–Apr 2018	B	0.069 (0.029, 0.136)	Panbio, Standard diagnostics, Yongin-si, South Korea
India	Chennai	05–40	5	800	indirect IgG ELISA	Rodriguez-Barraquer et al.^41^	Jun–Jul 2011	B	0.088 (0.042, 0.148)	Panbio and Novatec
India	Delhi	0–50+	4	200	capture IgG ELISA	Ranjan et al.^42^	Jul–Aug 2012	A	0.043 (0.036, 0.052)	Novatec, Germany
India	Pune	0–70+	13	1434	indirect IgG ELISA	Mishra et al.^44^	May–Jun 2017	A	0.078 (0.072, 0.084)	Panbio, Brisbane, Australia
India	Vaddu area	0–40+	5	702	indirect IgG ELISA	Shah et al.^45^	Feb–May2011	A	0.021 (0.018, 0.023)	Panbio Brisbane, Queensland, Australia
India	Chennai, Tamil Nadu	0–60	5	608	capture IgG ELISA	Bandi et al.^46^	Jan 2017–Oct 2018	A	0.007 (0.002, 0.022)	EVOLIS, BIoRad, GAC-Capture ELISA kit
India	Kerala (urban Kanjiarappally)	0–50+	6	469	IgG ELISA	Suresh et al.^47^	2016	A	0.024 (0.012, 0.045)	Bioline IgG/IgM WB RDT kits
India	Kerala (rural as Koruthodu and Erumeli in Kottayam district)	0–50+	6	469	IgG ELISA	Suresh et al.^47^	2016	A	0.016 (0.008, 0.032)	Bioline IgG/IgM WB RDT kits
India	Maharashtra (Vaddu area) 2014	05–15	2	819	indirect IgG ELISA	Shah et al.^48^	2014	A	0.017 (0.015, 0.021)	Panbio Inc., Brisbane, Queensland, Australia
India	Maharashtra (Vaddu area) 2016	05–15	2	819	indirect IgG ELISA	Shah et al.^48^	2016	A	0.024 (0.021, 0.028)	Panbio Inc., Brisbane, Queensland, Australia
India	Pune 2019	0–59	6	1654	indirect IgG ELISA	Alagarasu et al.^49^	2009	A	0.059 (0.031, 0.095)	PanBio dengue IgG indirect elisa kit (Abbott)
India	Pune 2009	0–59	6	797	indirect IgG ELISA	Alagarasu et al.^49^	2019	A	0.037 (0.021, 0.061)	PanBio dengue IgG indirect elisa kit (Abbott)
Indonesia	Denpasar Bali	0–40+	2	539	indirect IgG ELISA	Masyeni et al.^43^	Jul 2020–Jun 2021	A	0.036 (0.011, 0.081)	Panbio, Lubecl, Germany
Indonesia	Simpang Kiri (in Subulussalam, Nanggroe Aceh Darussalam)	0–18	18	106	indirect IgG ELISA	Tam et al.^50^	Oct–Nov 2014	A	0.126 (0.096, 0.162)	Panbio dengue IgG ELISA
Indonesia	Medan Denai (in Medan, Sumatera Utara)	0–18	18	107	indirect IgG ELISA	Tam et al.^50^	Oct–Nov 2014	A	0.188 (0.146, 0.242)	Panbio dengue IgG ELISA
Indonesia	Pauh (in Padang, Sumatera Barat)	0–18	18	106	indirect IgG ELISA	Tam et al.^50^	Oct–Nov 2014	A	0.164 (0.127, 0.210)	Panbio dengue IgG ELISA
Indonesia	Bungo Dani (in Bungo, Jambi)	0–18	18	107	indirect IgG ELISA	Tam et al.^50^	Oct–Nov 2014	A	0.225 (0.170, 0.296)	Panbio dengue IgG ELISA
Indonesia	Kalianda (in Lampung Selatan, Lampung)	0–18	18	105	indirect IgG ELISA	Tam et al.^50^	Oct–Nov 2014	A	0.073 (0.054, 0.097)	Panbio dengue IgG ELISA
Indonesia	Cikupa (in Tangerang, Banten)	0–18	18	107	indirect IgG ELISA	Tam et al.^50^	Oct–Nov 2014	A	0.262 (0.200, 0.336)	Panbio dengue IgG ELISA
Indonesia	Benda (in Tangerang, Banten)	0–18	18	101	indirect IgG ELISA	Tam et al.^50^	Oct–Nov 2014	A	0.122 (0.092, 0.157)	Panbio dengue IgG ELISA
Indonesia	Pesanggrahan (in Jakarta Selatan, Dki Jakarta)	0–18	18	105	indirect IgG ELISA	Tam et al.^50^	Oct–Nov 2014	A	0.196 (0.149, 0.255)	Panbio dengue IgG ELISA
Indonesia	Pulo Gadung (in Jakarta Timur, Dki Jakarta)	0–18	18	107	indirect IgG ELISA	Tam et al.^50^	Oct–Nov 2014	A	0.157 (0.121, 0.199)	Panbio dengue IgG ELISA
Indonesia	Kali Deres (in Jakarta Barat, Dki Jakarta)	0–18	18	107	indirect IgG ELISA	Tam et al.^50^	Oct–Nov 2014	A	0.143 (0.110, 0.185)	Panbio dengue IgG ELISA
Indonesia	Gunung Putri (in Bogor, Jawa Barat)	0–18	18	107	indirect IgG ELISA	Tam et al.^50^	Oct–Nov 2014	B	0.162 (0.108, 0.227)	Panbio dengue IgG ELISA
Indonesia	Banjaran (in Bandung, Jawa Barat)	0–18	18	107	indirect IgG ELISA	Tam et al.^50^	Oct–Nov 2014	A	0.124 (0.095, 0.159)	Panbio dengue IgG ELISA
Indonesia	Gunung Sari (in Cirebon, Jawa Barat)	0–18	18	107	indirect IgG ELISA	Tam et al.^50^	Oct–Nov 2014	A	0.220 (0.165, 0.291)	Panbio dengue IgG ELISA
Indonesia	Cikarang Utara (in Bekasi, Jawa Barat)	0–18	18	106	indirect IgG ELISA	Tam et al.^50^	Oct–Nov 2014	A	0.212 (0.161, 0.278)	Panbio dengue IgG ELISA
Indonesia	Bojongloa Kaler (in Bandung, Jawa Barat)	0–18	18	107	indirect IgG ELISA	Tam et al.^50^	Oct–Nov 2014	A	0.253 (0.190, 0.331)	Panbio dengue IgG ELISA
Indonesia	Bekasi Timur (in Bekasi, Jawa Barat)	0–18	18	107	indirect IgG ELISA	Tam et al.^50^	Oct–Nov 2014	A	0.154 (0.117, 0.199)	Panbio dengue IgG ELISA
Indonesia	Singaparna (in Tasikmalaya, Jawa Barat)	0–18	18	107	indirect IgG ELISA	Tam et al.^50^	Oct–Nov 2014	A	0.068 (0.050, 0.090)	Panbio dengue IgG ELISA
Indonesia	Trucuk (in Klaten, Jawa Tengah)	0–18	18	107	indirect IgG ELISA	Tam et al.^50^	Oct–Nov 2014	B	0.115 (0.072, 0.168)	Panbio dengue IgG ELISA
Indonesia	Pecangaan (in Jepara, Jawa Tengah)	0–18	18	107	indirect IgG ELISA	Tam et al.^50^	Oct–Nov 2014	A	0.225 (0.170, 0.298)	Panbio dengue IgG ELISA
Indonesia	Dukuhturi (in Tegal, Jawa Tengah)	0–18	18	107	indirect IgG ELISA	Tam et al.^50^	Oct–Nov 2014	A	0.288 (0.219, 0.385)	Panbio dengue IgG ELISA
Indonesia	Tegal Barat (in Tegal, Jawa Tengah)	0–18	18	107	indirect IgG ELISA	Tam et al.^50^	Oct–Nov 2014	A	0.296 (0.220, 0.398)	Panbio dengue IgG ELISA
Indonesia	Pulung (in Ponorogo, Jawa Timur)	0–18	18	107	indirect IgG ELISA	Tam et al.^50^	Oct–Nov 2014	A	0.047 (0.033, 0.064)	Panbio dengue IgG ELISA
Indonesia	Cluring (in Banyuwangi, Jawa Timur)	0–18	18	106	indirect IgG ELISA	Tam et al.^50^	Oct–Nov 2014	A	0.137 (0.104, 0.179)	Panbio dengue IgG ELISA
Indonesia	Ngoro (in Mojokerto, Jawa Timur)	0–18	18	107	indirect IgG ELISA	Tam et al.^50^	Oct–Nov 2014	A	0.233 (0.175, 0.309)	Panbio dengue IgG ELISA
Indonesia	Kalianget (in Sumenep, Jawa Timur)	0–18	18	105	indirect IgG ELISA	Tam et al.^50^	Oct–Nov 2014	A	0.132 (0.101, 0.170)	Panbio dengue IgG ELISA
Indonesia	Sawahan (in Surabaya, Jawa Timur)	0–18	18	107	indirect IgG ELISA	Tam et al.^50^	Oct–Nov 2014	A	0.258 (0.196, 0.336)	Panbio dengue IgG ELISA
Indonesia	Denpasar Selatan (in Denpasar, Bali)	0–18	18	107	indirect IgG ELISA	Tam et al.^50^	Oct–Nov 2014	A	0.147 (0.114, 0.189)	Panbio dengue IgG ELISA
Indonesia	Samarinda Ulu (in Samarinda, Kalimantan Timur)	0–18	18	107	indirect IgG ELISA	Tam et al.^50^	Oct–Nov 2014	A	0.118 (0.090, 0.150)	Panbio dengue IgG ELISA
Indonesia	Rantepao (in Toraja Utara, Sulawesi Selatan)	0–18	18	107	indirect IgG ELISA	Tam et al.^50^	Oct–Nov 2014	A	0.068 (0.050, 0.089)	Panbio dengue IgG ELISA
Indonesia	Kendari (in Kendari, Sulawesi Tenggara)	0–18	18	107	indirect IgG ELISA	Tam et al.^50^	Oct–Nov 2014	A	0.336 (0.250, 0.451)	Panbio dengue IgG ELISA
Indonesia	national	0–18	5	3194	indirect IgG ELISA	Sasmono et al.^51^	Oct–Nov 2014	A	0.149 (0.142, 0.156)	Panbio dengue IgG ELISA
Kenya	national	15–64	3	1091	indirect IgG ELISA	Ochieng et al.^52^	2007	A	0.004 (0.004, 0.005)	InBIos DENV Detect IgG ELISA
Kenya	Kilifi (Junju and Ngernya)	0–15	16	1847	IgG DENV2 ELISA	Unpublished Karanja et al.^53^	1998–2018	C	0.045 (0.038, 0.052)	in-house
Laos	Champasak	05–20+	4	236	IgG ELISA	Doum et al.^54^	May–Nov 2019	A	0.219 (0.154, 0.306)	Euroimmun, Germany
Laos	Savannakhet	05–20+	4	241	IgG ELISA	Doum et al.^54^	May–Nov 2019	A	0.081 (0.044, 0.133)	Euroimmun, Germany
Laos	Xay, Xiengngeun, Viengxay and Pek districts	06–50+	5	1136	Hemagglutination inhibition (HI)	Conlan et al.^55^	Jan–Mar 2009	D	0.001 (0.000, 0.005)	unspecified
Madagascar	Ambovombe	18–45+	4	60	indirect IgG ELISA	Broban et al.^56^	2011–2013	A	0.001 (0.00, 0.003)	in-house
Madagascar	Ambatondrazaka	18–45+	4	60	indirect IgG ELISA	Broban et al.^56^	2011–2013	A	0.001 (0.000, 0.002)	in-house
Madagascar	Antsohihy	18–45+	4	60	indirect IgG ELISA	Broban et al.^56^	2011–2013	A	0.002 (0.001, 0.004)	in-house
Madagascar	Anjozorobe	18–45+	4	60	indirect IgG ELISA	Broban et al.^56^	2011–2013	A	0.000 (0.000, 0.002)	in-house
Madagascar	Antsirabe	18–45+	4	60	indirect IgG ELISA	Broban et al.^56^	2011–2013	A	0.000 (0.000, 0.001)	in-house
Madagascar	Belo	18–45+	4	60	indirect IgG ELISA	Broban et al.^56^	2011–2013	A	0.002 (0.001, 0.004)	in-house
Madagascar	Ambato-Boeny	18–45+	4	60	indirect IgG ELISA	Broban et al.^56^	2011–2013	A	0.002 (0.001, 0.005)	in-house
Madagascar	Ambositra	18–45+	4	60	indirect IgG ELISA	Broban et al.^56^	2011–2013	A	0.000 (0.000, 0.001)	in-house
Madagascar	Antananarivo-Renivohitra	18–45+	4	60	indirect IgG ELISA	Broban et al.^56^	2011–2013	A	0.001 (0.000, 0.002)	in-house
Madagascar	Antsiranana	18-45+	4	60	indirect IgG ELISA	Broban et al.^56^	2011–2013	A	0.004 (0.002, 0.007)	in-house
Madagascar	Ampanihy	18–45+	4	60	indirect IgG ELISA	Broban et al.^56^	2011–2013	A	0.000 (0.000, 0.002)	in-house
Madagascar	Farafangana	18–45+	4	60	indirect IgG ELISA	Broban et al.^56^	2011–2013	A	0.002 (0.001, 0.005)	in-house
Madagascar	Fianarantsoa	18–45+	4	60	indirect IgG ELISA	Broban et al.^56^	2011–2013	A	0.000 (0.000, 0.001)	in-house
Madagascar	Ihosy	18–45+	4	60	indirect IgG ELISA	Broban et al.^56^	2011–2013	A	0.002 (0.001, 0.004)	in-house
Madagascar	Maevatanana	18–45+	4	60	indirect IgG ELISA	Broban et al.^56^	2011–2013	A	0.000 (0.000, 0.002)	in-house
Madagascar	Mandritsara	18–45+	4	60	indirect IgG ELISA	Broban et al.^56^	2011–2013	A	0.000 (0.000, 0.001)	in-house
Madagascar	Morondava	18–45+	4	60	indirect IgG ELISA	Broban et al.^56^	2011–2013	A	0.002 (0.001, 0.004)	in-house
Madagascar	Miandrivazo	18–45+	4	60	indirect IgG ELISA	Broban et al.^56^	2011–2013	A	0.001 (0.000, 0.002)	in-house
Madagascar	Mahajanga	18–45+	4	60	indirect IgG ELISA	Broban et al.^56^	2011–2013	A	0.001 (0.000, 0.002)	in-house
Madagascar	Mananjary	18–45+	4	60	indirect IgG ELISA	Broban et al.^56^	2011–2013	A	0.004 (0.002, 0.007)	in-house
Madagascar	Morombe	18–45+	4	60	indirect IgG ELISA	Broban et al.^56^	2011–2013	A	0.001 (0.000, 0.003)	in-house
Madagascar	Moramanga	18–45+	4	60	indirect IgG ELISA	Broban et al.^56^	2011–2013	A	0.001 (0.000, 0.003)	in-house
Madagascar	Nosy-Be	18–45+	4	60	indirect IgG ELISA	Broban et al.^56^	2011–2013	A	0.017 (0.011, 0.024)	in-house
Madagascar	Sambava	18–45+	4	60	indirect IgG ELISA	Broban et al.^56^	2011–2013	A	0.001 (0.000, 0.002)	in-house
Madagascar	Tsiroanomandidy	18–45+	4	60	indirect IgG ELISA	Broban et al.^56^	2011–2013	A	0.001 (0.000, 0.003)	in-house
Madagascar	Taolagnaro	18–45+	4	60	indirect IgG ELISA	Broban et al.^56^	2011–2013	A	0.002 (0.001, 0.005)	in-house
Madagascar	Toamasina	18–45+	4	60	indirect IgG ELISA	Broban et al.^56^	2011–2013	A	0.016 (0.010, 0.023)	in-house
Madagascar	Toliary	18–45+	4	60	indirect IgG ELISA	Broban et al.^56^	2011–2013	A	0.001 (0.000, 0.003)	in-house
Malaysia	Federal territory of Kuala Lumpur, Perak, Kedah, Penang, Johor, Pahang, Kelantan and Sabah	07–18	4	1417	capture IgG ELISA	Tiong et al.^57^	2008–2009	A	0.010 (0.008, 0.012)	Standard Diagnostics, Korea
Malaysia	Damansara Damai (in the Petaling Jaya district)	0–25+	2	82	IgG ELISA	Selvarajoo et al.^58^	Sep 2018–Jan 2019	A	0.033 (0.010, 0.075)	Focus Diagnostics Inc, Cypress, CA, USA
Malaysia	Peninsular Malaysia (urban)	35–74	4	1417	indirect IgG ELISA	Azami et al.^59^	2006–2012	A	0.038 (0.036, 0.041)	Abbott Laboratories, Abbott Pakr, IL, USA
Malaysia	Peninsular Malaysia (rural)	35–74	4	1181	indirect IgG ELISA	Azami et al.^59^	2006–2012	A	0.034 (0.032, 0.036)	Abbott Laboratories, Abbott Pakr, IL, USA
Malaysia	Petaling district	0-60+	8	500	indirect IgG ELISA	Ng et al.^60^	Aug–Oct 2018	A	0.059 (0.052, 0.066)	Panbio, Abbott, Chicago, IL, USA
Malaysia	Sungai Segamat	18–55+	5	277	capture IgG ELISA	Dhanoa et al.^61^	Apr–May 2015	A	0.029 (0.025, 0.033)	Panbio indirect and Capture elisa Alere, Brisbane, Queensland, Australia
Malaysia	forest areas of Peninsular Malaysia	0–13+	2	491	capture IgG ELISA	Abd-Jamil et al.^62^	Nov 2007–Oct 2010	A	0.023 (0.005, 0.074)	Standard Diagnostics Dengue IgG Capture ELISA (SD, Korea: 11EK10)
Mexico	Acapulco	06–17	3	75	indirect IgG ELISA	Amaya-Larios et al.^63^	Feb–Jul 2016	A	0.225 (0.164, 0.315)	Panbio, Alere, Waltham, MA, USA
Mexico	Ahome	06–17	3	17	indirect IgG ELISA	Amaya-Larios et al.^63^	Feb–Jul 2016	A	0.054 (0.026, 0.100)	Panbio, Alere, Waltham, MA, USA
Mexico	Apaseo el Grande	06–17	3	23	indirect IgG ELISA	Amaya-Larios et al.^63^	Feb–Jul 2016	A	0.003 (0.000, 0.017)	Panbio, Alere, Waltham, MA, USA
Mexico	Baja California	06–17	3	23	indirect IgG ELISA	Amaya-Larios et al.^63^	Feb–Jul 2016	A	0.053 (0.013, 0.213)	Panbio, Alere, Waltham, MA, USA
Mexico	Campeche	06–17	3	23	indirect IgG ELISA	Amaya-Larios et al.^63^	Feb–Jul 2016	A	0.118 (0.083, 0.181)	Panbio, Alere, Waltham, MA, USA
Mexico	Cancun	06–17	3	29	indirect IgG ELISA	Amaya-Larios et al.^63^	Feb–Jul 2016	A	0.119 (0.072, 0.183)	Panbio, Alere, Waltham, MA, USA
Mexico	Cardenas	06–17	3	53	indirect IgG ELISA	Amaya-Larios et al.^63^	Feb–Jul 2016	A	0.131 (0.092, 0.181)	Panbio, Alere, Waltham, MA, USA
Mexico	Chetumal	06–17	3	25	indirect IgG ELISA	Amaya-Larios et al.^63^	Feb–Jul 2016	A	0.079 (0.046, 0.128)	Panbio, Alere, Waltham, MA, USA
Mexico	Chilpancingo	06–17	3	36	indirect IgG ELISA	Amaya-Larios et al.^63^	Feb–Jul 2016	A	0.047 (0.025, 0.078)	Panbio, Alere, Waltham, MA, USA
Mexico	Ciudad Acuña	06–17	3	18	indirect IgG ELISA	Amaya-Larios et al.^63^	Feb–Jul 2016	A	0.034 (0.011, 0.074)	Panbio, Alere, Waltham, MA, USA
Mexico	Ciudad Apodaca	06–17	3	19	indirect IgG ELISA	Amaya-Larios et al.^63^	Feb–Jul 2016	A	0.011 (0.002, 0.031)	Panbio, Alere, Waltham, MA, USA
Mexico	Ciudad Benito Juarez	06–17	3	15	indirect IgG ELISA	Amaya-Larios et al.^63^	Feb–Jul 2016	A	0.013 (0.002, 0.044)	Panbio, Alere, Waltham, MA, USA
Mexico	Ciudad del carmen	06–17	3	25	indirect IgG ELISA	Amaya-Larios et al.^63^	Feb–Jul 2016	A	0.093 (0.053, 0.150)	Panbio, Alere, Waltham, MA, USA
Mexico	Ciudad Victoria	06–17	3	47	indirect IgG ELISA	Amaya-Larios et al.^63^	Feb–Jul 2016	A	0.029 (0.015, 0.050)	Panbio, Alere, Waltham, MA, USA
Mexico	Coatzacoalcos	06–17	3	12	indirect IgG ELISA	Amaya-Larios et al.^63^	Feb–Jul 2016	A	0.069 (0.027, 0.143)	Panbio, Alere, Waltham, MA, USA
Mexico	Comalcalco	06–17	3	35	indirect IgG ELISA	Amaya-Larios et al.^63^	Feb–Jul 2016	A	0.104 (0.064, 0.157)	Panbio, Alere, Waltham, MA, USA
Mexico	Cordoba	06–17	3	13	indirect IgG ELISA	Amaya-Larios et al.^63^	Feb–Jul 2016	A	0.151 (0.074, 0.291)	Panbio, Alere, Waltham, MA, USA
Mexico	Cuernavaca	06–17	3	19	indirect IgG ELISA	Amaya-Larios et al.^63^	Feb–Jul 2016	A	0.081 (0.043, 0.140)	Panbio, Alere, Waltham, MA, USA
Mexico	Culiacan	06–17	3	12	indirect IgG ELISA	Amaya-Larios et al.^63^	Feb–Jul 2016	A	0.128 (0.058, 0.243)	Panbio, Alere, Waltham, MA, USA
Mexico	Ebano	06–17	3	34	indirect IgG ELISA	Amaya-Larios et al.^63^	Feb–Jul 2016	A	0.025 (0.011, 0.049)	Panbio, Alere, Waltham, MA, USA
Mexico	El Salto	06–17	3	18	indirect IgG ELISA	Amaya-Larios et al.^63^	Feb–Jul 2016	A	0.007 (0.001, 0.023)	Panbio, Alere, Waltham, MA, USA
Mexico	Empalme Escobedo	06–17	3	18	indirect IgG ELISA	Amaya-Larios et al.^63^	Feb–Jul 2016	A	0.003 (0.000, 0.016)	Panbio, Alere, Waltham, MA, USA
Mexico	Etzatlan	06–17	3	40	indirect IgG ELISA	Amaya-Larios et al.^63^	Feb–Jul 2016	A	0.002 (0.000, 0.010)	Panbio, Alere, Waltham, MA, USA
Mexico	General Escobedo	06–17	3	29	indirect IgG ELISA	Amaya-Larios et al.^63^	Feb–Jul 2016	A	0.007 (0.001, 0.022)	Panbio, Alere, Waltham, MA, USA
Mexico	Guadalajara	06–17	3	24	indirect IgG ELISA	Amaya-Larios et al.^63^	Feb–Jul 2016	A	0.031 (0.013, 0.059)	Panbio, Alere, Waltham, MA, USA
Mexico	Guaymas	06–17	3	23	indirect IgG ELISA	Amaya-Larios et al.^63^	Feb–Jul 2016	A	0.077 (0.040, 0.132)	Panbio, Alere, Waltham, MA, USA
Mexio	Guerrero	06–17	3	23	indirect IgG ELISA	Amaya-Larios et al.^63^	Feb–Jul 2016	A	0.061 (0.026, 0.190)	Panbio, Alere, Waltham, MA, USA
Mexico	Hermosillo	06–17	3	55	indirect IgG ELISA	Amaya-Larios et al.^63^	Feb–Jul 2016	A	0.016 (0.007, 0.029)	Panbio, Alere, Waltham, MA, USA
Mexico	Macuspana	06–17	3	18	indirect IgG ELISA	Amaya-Larios et al.^63^	Feb–Jul 2016	A	0.121 (0.065, 0.205)	Panbio, Alere, Waltham, MA, USA
Mexico	Mapastepec	06–17	3	71	indirect IgG ELISA	Amaya-Larios et al.^63^	Feb–Jul 2016	A	0.451 (0.304, 0.706)	Panbio, Alere, Waltham, MA, USA
Mexico	Matamoros	06–17	3	49	indirect IgG ELISA	Amaya-Larios et al.^63^	Feb–Jul 2016	A	0.034 (0.019, 0.056)	Panbio, Alere, Waltham, MA, USA
Mexico	Mazatlan	06–17	3	12	indirect IgG ELISA	Amaya-Larios et al.^63^	Feb–Jul 2016	A	0.071 (0.032, 0.139)	Panbio, Alere, Waltham, MA, USA
Mexico	Merida	06–17	3	99	indirect IgG ELISA	Amaya-Larios et al.^63^	Feb–Jul 2016	A	0.071 (0.054, 0.093)	Panbio, Alere, Waltham, MA, USA
Mexico	Mexicali	06–17	3	19	indirect IgG ELISA	Amaya-Larios et al.^63^	Feb–Jul 2016	A	0.007 (0.001, 0.023)	Panbio, Alere, Waltham, MA, USA
Mexico	Monterrey	06–17	3	60	indirect IgG ELISA	Amaya-Larios et al.^63^	Feb–Jul 2016	A	0.015 (0.008, 0.026)	Panbio, Alere, Waltham, MA, USA
Mexico	Navolato	06–17	3	29	indirect IgG ELISA	Amaya-Larios et al.^63^	Feb–Jul 2016	A	0.129 (0.080, 0.198)	Panbio, Alere, Waltham, MA, USA
Mexico	Nuevo laredo	06–17	3	16	indirect IgG ELISA	Amaya-Larios et al.^63^	Feb–Jul 2016	A	0.026 (0.009, 0.055)	Panbio, Alere, Waltham, MA, USA
Mexico	Oaxaca	06–17	3	36	indirect IgG ELISA	Amaya-Larios et al.^63^	Feb–Jul 2016	A	0.030 (0.015, 0.056)	Panbio, Alere, Waltham, MA, USA
Mexico	Orizaba	06–17	3	25	indirect IgG ELISA	Amaya-Larios et al.^63^	Feb–Jul 2016	A	0.011 (0.003, 0.029)	Panbio, Alere, Waltham, MA, USA
Mexico	Piedras Negras	06–17	3	39	indirect IgG ELISA	Amaya-Larios et al.^63^	Feb–Jul 2016	A	0.002 (0.000, 0.008)	Panbio, Alere, Waltham, MA, USA
Mexico	Poza Rica	06–17	3	22	indirect IgG ELISA	Amaya-Larios et al.^63^	Feb–Jul 2016	A	0.131 (0.077, 0.215)	Panbio, Alere, Waltham, MA, USA
Mexico	Progreso	06–17	3	38	indirect IgG ELISA	Amaya-Larios et al.^63^	Feb–Jul 2016	A	0.108 (0.069, 0.161)	Panbio, Alere, Waltham, MA, USA
Mexico	Reynosa	06–17	3	32	indirect IgG ELISA	Amaya-Larios et al.^63^	Feb–Jul 2016	A	0.042 (0.021, 0.074)	Panbio, Alere, Waltham, MA, USA
Mexico	Salamanca	06–17	3	60	indirect IgG ELISA	Amaya-Larios et al.^63^	Feb–Jul 2016	A	0.001 (0.000, 0.007)	Panbio, Alere, Waltham, MA, USA
Mexico	San Francisco de Campeche	06–17	3	28	indirect IgG ELISA	Amaya-Larios et al.^63^	Feb–Jul 2016	A	0.171 (0.104, 0.276)	Panbio, Alere, Waltham, MA, USA
Mexico	San Gabriel Chilac	06–17	3	29	indirect IgG ELISA	Amaya-Larios et al.^63^	Feb–Jul 2016	A	0.031 (0.015, 0.055)	Panbio, Alere, Waltham, MA, USA
Mexico	San Jose del Cabo	06–17	3	21	indirect IgG ELISA	Amaya-Larios et al.^63^	Feb–Jul 2016	A	0.052 (0.027, 0.091)	Panbio, Alere, Waltham, MA, USA
Mexico	San Pedro	06–17	3	12	indirect IgG ELISA	Amaya-Larios et al.^63^	Feb–Jul 2016	A	0.058 (0.025, 0.116)	Panbio, Alere, Waltham, MA, USA
Mexico	San Pedro Pochutla	06–17	3	19	indirect IgG ELISA	Amaya-Larios et al.^63^	Feb–Jul 2016	A	0.152 (0.085, 0.260)	Panbio, Alere, Waltham, MA, USA
Mexico	Santa Catarina	06–17	3	22	indirect IgG ELISA	Amaya-Larios et al.^63^	Feb–Jul 2016	A	0.013 (0.003, 0.035)	Panbio, Alere, Waltham, MA, USA
Mexico	Tanquián de Escobedo	06–17	3	15	indirect IgG ELISA	Amaya-Larios et al.^63^	Feb–Jul 2016	A	0.003 (0.000, 0.018)	Panbio, Alere, Waltham, MA, USA
Mexico	Tapachula	06–17	3	75	indirect IgG ELISA	Amaya-Larios et al.^63^	Feb–Jul 2016	A	0.274 (0.202, 0.370)	Panbio, Alere, Waltham, MA, USA
Mexico	Tecpan de Galeana	06–17	3	44	indirect IgG ELISA	Amaya-Larios et al.^63^	Feb–Jul 2016	A	0.235 (0.160, 0.342)	Panbio, Alere, Waltham, MA, USA
Mexico	Teocaltiche	06–17	3	26	indirect IgG ELISA	Amaya-Larios et al.^63^	Feb–Jul 2016	A	0.003 (0.000, 0.015)	Panbio, Alere, Waltham, MA, USA
Mexico	Tepic	06–17	3	36	indirect IgG ELISA	Amaya-Larios et al.^63^	Feb–Jul 2016	A	0.055 (0.030, 0.089)	Panbio, Alere, Waltham, MA, USA
Mexico	Tijuana	06–17	3	41	indirect IgG ELISA	Amaya-Larios et al.^63^	Feb–Jul 2016	A	0.004 (0.001, 0.015)	Panbio, Alere, Waltham, MA, USA
Mexico	Tlapacoyan	06–17	3	20	indirect IgG ELISA	Amaya-Larios et al.^63^	Feb–Jul 2016	A	0.124 (0.068, 0.210)	Panbio, Alere, Waltham, MA, USA
Mexico	Tonala	06–17	3	35	indirect IgG ELISA	Amaya-Larios et al.^63^	Feb–Jul 2016	A	0.024 (0.011, 0.045)	Panbio, Alere, Waltham, MA, USA
Mexico	Tuxtla Gutierrez	06–17	3	33	indirect IgG ELISA	Amaya-Larios et al.^63^	Feb–Jul 2016	A	0.159 (0.102, 0.237)	Panbio, Alere, Waltham, MA, USA
Mexico	Uruapan	06–17	3	44	indirect IgG ELISA	Amaya-Larios et al.^63^	Feb–Jul 2016	A	0.004 (0.001, 0.014)	Panbio, Alere, Waltham, MA, USA
Mexico	Valle de Santiago	06–17	3	25	indirect IgG ELISA	Amaya-Larios et al.^63^	Feb–Jul 2016	A	0.002 (0.000, 0.011)	Panbio, Alere, Waltham, MA, USA
Mexico	Veracruz	06–17	3	33	indirect IgG ELISA	Amaya-Larios et al.^63^	Feb–Jul 2016	A	0.130 (0.085, 0.194)	Panbio, Alere, Waltham, MA, USA
Mexico	Xalapa	06–17	3	78	indirect IgG ELISA	Amaya-Larios et al.^63^	Feb–Jul 2016	A	0.005 (0.002, 0.010)	Panbio, Alere, Waltham, MA, USA
Mexico	Zacatepec	06–17	3	17	indirect IgG ELISA	Amaya-Larios et al.^63^	Feb–Jul 2016	A	0.046 (0.018, 0.095)	Panbio, Alere, Waltham, MA, USA
Mexico	Zamora	06–17	3	16	indirect IgG ELISA	Amaya-Larios et al.^63^	Feb–Jul 2016	A	0.032 (0.012, 0.066)	Panbio, Alere, Waltham, MA, USA
Mexico	Zapopan	06–17	3	32	indirect IgG ELISA	Amaya-Larios et al.^63^	Feb–Jul 2016	A	0.005 (0.001, 0.016)	Panbio, Alere, Waltham, MA, USA
Mexico	Zaragoza	06–17	3	30	indirect IgG ELISA	Amaya-Larios et al.^63^	Feb–Jul 2016	A	0.019 (0.007, 0.042)	Panbio, Alere, Waltham, MA, USA
Mexico	Zihuatanejo	06–17	3	25	indirect IgG ELISA	Amaya-Larios et al.^63^	Feb–Jul 2016	A	0.649 (0.245, 0.984)	Panbio, Alere, Waltham, MA, USA
Mexico	Yucatan	0–50	5	1667	indirect IgG ELISA	Pavia-Ruz et al.^64^	Jan–Jun 2014	A	0.045 (0.020, 0.081)	Panbio
Mexico	State of Morelos	05–89	15	928	indirect IgG ELISA	Amaya-Larios2 et al.^65^	Jun–Nov 2011	A	0.044 (0.018, 0.087)	Panbio
Nigeria	Osogbo, Osun State	15–33	2	89	IgG ELISA	Sule et al.^66^	Nov 2014–Aug 2015	A	0.018 (0.013, 0.026)	DIA.PRO Diagnostic Bioproblems Srl., Milano, Italy
Pakistan	Lahore	0–12	3	400	IgG ELISA	Mohsin et al.^67^	2015∗	A	0.060 (0.050, 0.070)	Calbiotech, USA
Peru	Iquitos	05–60+	12	3127	PRNT	Forshey et al.^68^	Mar–Jun 2010	D	0.051 (0.045, 0.066)	unspecified
Saudi Arabia	Makkah	0–30+	3	478	capture IgG ELISA	Al-Raddadi et al.^69^	Sep 2016–Jan 2017	B	0.021 (0.007, 0.058)	Standard Diagnostics, Korea
Saudi Arabia	Madinah	0–30+	3	233	capture IgG ELISA	Al-Raddadi et al.^69^	Sep 2016–Jan 2018	A	0.004 (0.003, 0.006)	Standard Diagnostics, Korea
Saudi Arabia	Jeddah	0–30+	3	462	capture IgG ELISA	Al-Raddadi et al.^69^	Sep 2016–Jan 2019	A	0.007 (0.006, 0.009)	Standard Diagnostics, Korea
Saudi Arabia	Jizan	0–30+	3	537	capture IgG ELISA	Al-Raddadi et al.^69^	Sep 2016–Jan 2020	B	0.023 (0.007, 0.060)	Standard Diagnostics, Korea
Saudi Arabia	Jeddah	0–50+	6	1904	indirect IgG ELISA	Jamjoom et al.^70^	2015∗	A	0.017 (0.016, 0.018)	Panbio, Australia
Singapore	national	01–15	3	1200	IgG ELISA	Ang et al.^71^	Aug 2008–Jul 2010	A	0.020 (0.013, 0.030)	Euroimmun, Germany
Singapore	blood service	16–60	9	3627	indirect IgG ELISA	Low et al.^72^	Dec 2009–Feb 2010	A	0.049 (0.012, 0.148)	Panbio
Singapore	national	18–79	6	1000	IgG ELISA	Ang2 et al.^73^	Mar–Jun 2010	B	0.034 (0.007, 0.143)	Euroimmun, Germany
Sri Lanka	City of Colombo	01–12	5	799	IgG ELISA	Tissera et al.^74^	Nov 2008–Jan 2010	A	0.149 (0.076, 0.248)	in-house
Sri Lanka	Ramalana community in Colombo district	18–80	4	150	indirect IgG ELISA	Abeygoonawardena^75^	2017	A	0.077 (0.061, 0.099)	Panbio (Alere, Australia)
Sri Lanka	Trincomalee district	10–19	3	255	indirect IgG ELISA	Jeewandara et al.^76^	Sep 2022–Mar 2023	A	0.056 (0.047, 0.066)	Panbio
Sri Lanka	Jaffna district	10–19	3	300	indirect IgG ELISA	Jeewandara et al.^76^	Sep 2022–Mar 2023	A	0.032 (0.035, 0.037)	Panbio
Sri Lanka	Kurunegala district	10–19	3	828	indirect IgG ELISA	Jeewandara et al.^76^	Sep 2022–Mar 2023	A	0.012 (0.010, 0.014)	Panbio
Sri Lanka	Matara district	10–19	3	506	indirect IgG ELISA	Jeewandara et al.^76^	Sep 2022–Mar 2023	A	0.014 (0.011, 0.017)	Panbio
Sri Lanka	Ratnapura district	10–19	3	495	indirect IgG ELISA	Jeewandara et al.^76^	Sep 2022–Mar 2023	A	0.025 (0.021, 0.030)	Panbio
Sri Lanka	Polonaruwa district	10–19	3	257	indirect IgG ELISA	Jeewandara et al.^76^	Sep 2022–Mar 2023	A	0.022 (0.017, 0.028)	Panbio
Sri Lanka	Gampha district	10–19	3	1355	indirect IgG ELISA	Jeewandara et al.^76^	Sep 2022–Mar 2023	A	0.025 (0.023, 0.028)	Panbio
Sri Lanka	Kandy district	10–19	3	681	indirect IgG ELISA	Jeewandara et al.^76^	Sep 2022–Mar 2023	A	0.013 (0.011, 0.015)	Panbio
Sri Lanka	Badulla district	10–19	3	502	indirect IgG ELISA	Jeewandara et al.^76^	Sep 2022–Mar 2023	A	0.011 (0.009, 0.014)	Panbio
Sri Lanka	Sri Jayewadenpura	06–80	11	1671	indirect IgG ELISA	Jeewandara2 et al.^77^	2013–2014	A	0.068 (0.063, 0.073)	Panbio dengue IgG indirect ELISA—Australia
Taiwan	Taipei, Taoyuan, Tainan	0–70+	8	1308	IgG ELISA	Lee et al.^78^	Sep–Oct 2010	A	0.004 (0.002, 0.011)	Euroimmun, Germany
Taiwan	Nanzih district, Kaohsiung City	0–79	7	154	IgG ELISA	Tsai et al.^79^	2015–2016	A	0.001 (0.001, 0.001)	InBios International
Taiwan	Sanmin district, Kaohsiung City	0–79	7	132	IgG ELISA	Tsai et al.^79^	2015–2016	A	0.005 (0.003, 0.007)	InBios International
Tanzania	Buhigwe, Kalambo, Kilindi, Kinondoni, Kondoa, Kyela, Mvomero and Ukerewe	0–42+	3	1818	Indirect IgG ELISA	Mwanyika et al.^80^	Apr–Nov 2018	A	0.011 (0.003, 0.035)	Euroimmun, Lubeck, Germany
Tanzania	Zanzibar	0–36+	3	500	indirect IgG ELISA	Vairo et al.^81^	2010	B	0.114 (0.034, 0.254)	Euroimmun, Germany
Thailand	Mukdahn	05–20+	4	304	IgG ELISA	Doum et al.^54^	May–Nov 2019	A	0.112 (0.089, 0.143)	Euroimmun, Germany
Thailand	Ubon	05–20+	4	290	IgG ELISA	Doum et al.^54^	May–Nov 2019	A	0.119 (0.092, 0.155)	Euroimmun, Germany
Thailand	South (Narathiwat and Trang)	0–45+	3	406	IgG ELISA	Vongpunsawad et al.^82^	Apr–Oct 2014	A	0.070 (0.060, 0.081)	Euroimmun, Germany
Thailand	Central (Ayutthaya and Lop Buri)	0–45+	3	429	IgG ELISA	Vongpunsawad et al.^82^	Apr–Oct 2014	A	0.070 (0.061, 0.081)	Euroimmun, Germany
Thailand	Ratchaburi province	01–55	6	1814	indirect IgG ELISA	Limkittikul et al.^83^	2012–2015	A	0.136 (0.127, 0.146)	in-house
Thailand	Ratchaburi province	10–22	3	115	IgG ELISA	Limothai U et al.^84^	Aug 2019–Aug 2020	A	0.094 (0.074, 0.118)	Bioline, Korea
Venezuela	Cana de Azucar	05–30	5	2002	Hemagglutination inhibition (HI)	Velasco-Salas et al.^85^	Aug 2010–Jan 2011	B	0.059 (0.015, 0.181)	in-house
Vietnam	Nha Trang City, Nih Hoa district and Dien Khanh district (in Khanh Hoa province)	03–60	6	1479	indirect IgG ELISA	Mai et al.^86^	Oct 2011	A	0.012 (0.005, 0.029)	Panbio dengue duo cassette (Alere, Brisbane, Australia)

The estimated force of infection (FOI) is expressed in years and reported as median and 95% credible intervals (CrI). We used ∗ when the date of the survey was not specified, and in these instances we report the year of publication. We used + when the upper bound of the age group was not specified.

### Force of infection estimates

**Fig. 3:**
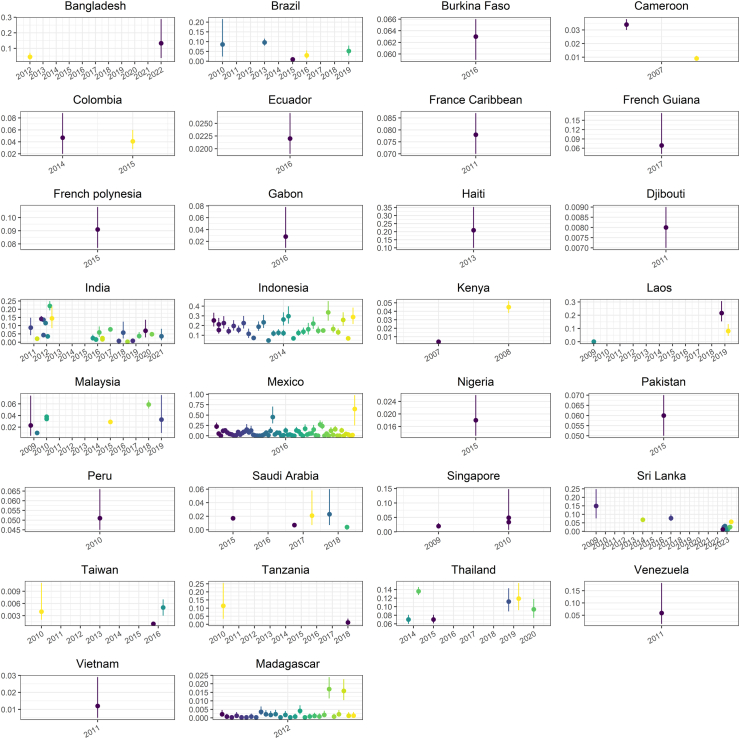
**FOI estimates obtained in this study, grouped by country and using the best fitting model**. The x-axis shows the year of the serological survey and the y-axis shows the median (point) and 95% CrI (line) of the FOI estimates. Different locations within the same country are presented in different colours.

Table 1 and Fig. 3 summarise the FOI estimates obtained for each location and country under the best fitting model, which was selected according to the DIC metric. The FOI estimates and DIC values obtained with each model version are presented in the Supplementary data (Fig. S8 and Tables S3 and S4).

As shown in Fig. 3, this study generated several FOI estimates from Indonesia, Madagascar, Mexico and India.

The 66 papers selected in this study generated a total of 219 dengue FOI estimates which were most frequently obtained using model A (Table 1). For the four datasets (from Kenya,^53^ Brazil,^24^ French Guiana,^35^ French Polynesia^36^) reporting age-specific positivity rates (not the number of positive and tested subjects) the FOI was estimated with model C, while for the two papers reporting serotype-specific seroprevalence estimates using the PRNT assay (in Peru^68^) and the Hemagglutination inhibition (HI) assay (in Laos^55^), we used model D to estimate the overall FOI.

Overall, Laos, Haiti, India, Indonesia and Mexico showed the highest yearly FOI estimates, with average values around 0.2, which correspond to an expected average age of first infection of around 5 years. On the other hand, Taiwan was the country with the lowest estimated yearly FOI, with an average estimate around 0.0011 (95% CrI 0.0011, 0.0013).

**Fig. 4:**
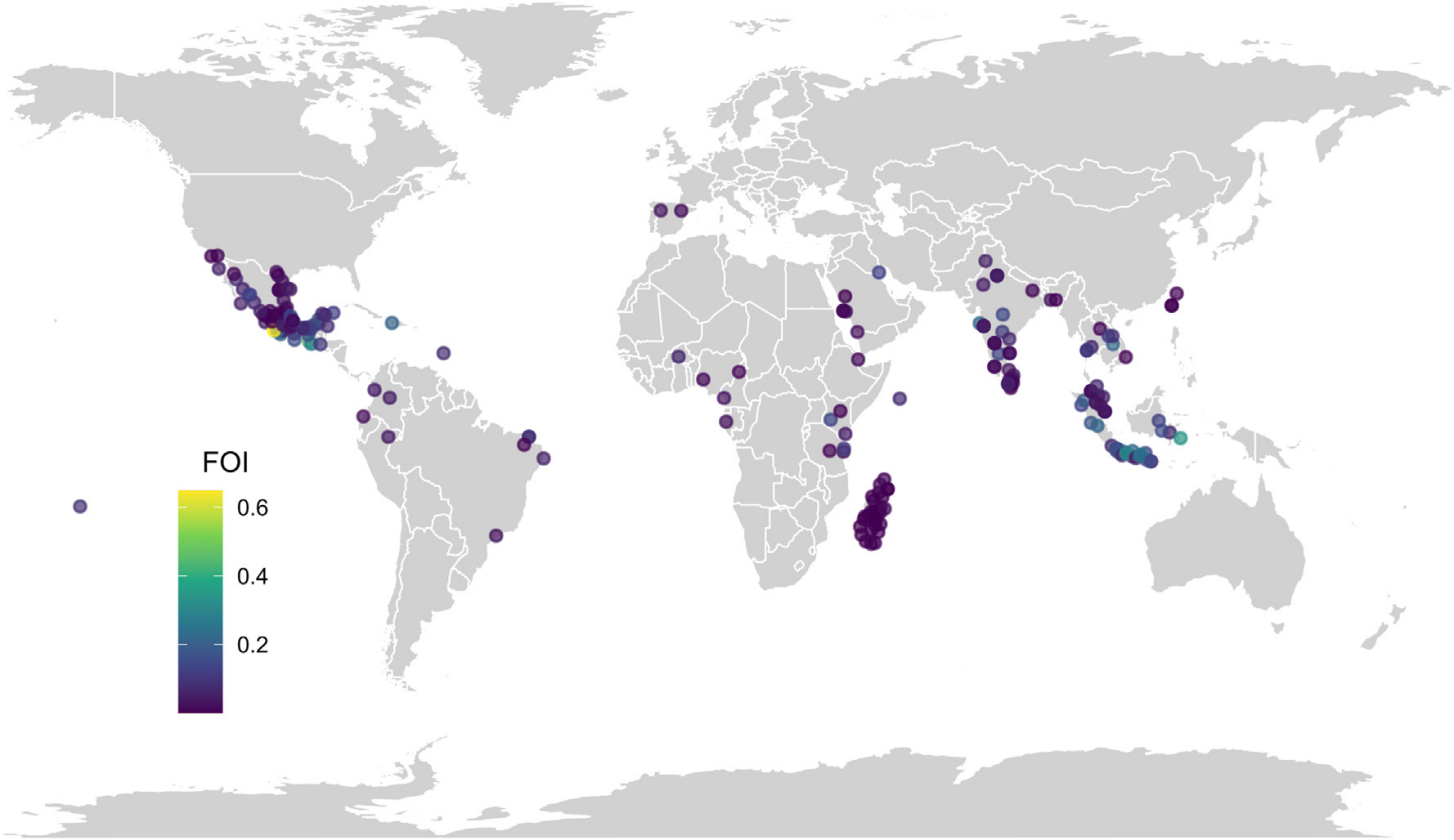
**Worldwide distribution and geolocation of the average force of infection (FOI) estimates obtained in this study**. Average FOI estimates obtained using the best model according to the DIC criterion, as summarized in Table 1.

Fig. 4 shows the geographical distribution of the dengue serosurveys identified in this analysis and the resulting FOI estimates. We find evidence of a high risk of infection across India, Indonesia, Laos and Haiti, with the highest FOI estimate being estimated in Kendari (Indonesia), with a median FOI of 0.34 (95% CrI 0.25–0.45). Lower transmission intensities were found in countries such as Taiwan (median FOI between 0.001 and 0.005) and Saudi Arabia (median FOI between 0.008 and 0.017), which are located on the outskirts of tropical and subtropical regions (Fig. 4). Interestingly, we estimated among the lowest FOI estimates in the North-east of India (median FOI of 0.002) and in Laos (median FOI of 0.001), which are also the countries with the highest dengue FOI estimates—this highlights the vast differences in the intensity of dengue transmission within these two countries.

The patterns of dengue transmission in Latin America and the Caribbean are heterogeneous, with Haiti showing a high FOI estimate of 0.209 (95% CrI 0.100–0.352), while some locations in Brazil (e.g., the state of Ceara) and Mexico (e.g., Salamanca) showing the lowest FOI in these areas (with a mean of 0.009 (95% CrI 0.007, 0.011) and 0.001 (95% CrI 0.00, 0.007), respectively).

## Discussion

Age-stratified serological surveys represent an ideal surveillance tool to estimate the risk of dengue infection and to reconstruct the age-specific susceptibility profile and historical circulation of dengue in the surveyed populations.^54^ In this study, 66 publications were retrieved from published databases, which provided 219 age-stratified dengue seroprevalence datasets collected between 2006 and 2023 from 30 different countries including 38 from Africa, 97 from Asia, 72 from North America, 1 from Oceania, and 11 from South America. Compared to the database collated in Imai et al.,^4^ we found five times more FOI estimates from studies published since 2014, demonstrating an increasing number of dengue serosurveys being conducted globally in the last decade.

We found that the FOI estimates obtained with models A and B (which assumed antibodies did (B) or did not (A) wane over time) were consistent (Fig. S13). As expected, we found high heterogeneities in the average FOI within and between countries.^3^ The newly identified age-stratified seroprevalence datasets from the Ratchaburi province of Thailand and Singapore generated FOI estimates that are consistent with the estimates obtained from previous studies.^4^ On the other hand, the FOI estimates obtained in this study for Lahore (Pakistan) and Colombo city (Sri Lanka) are higher than those previously published in Imai et al.,^4^ highlighting changes in FOI, potentially linked with the geographical expansion of dengue circulation,^41^ and increases in urbanization and population density.^45^ As for the other countries previously studied in Imai et al.^4^—Brazil, Laos, India, Indonesia, Haiti and Mexico—we identified new age-stratified serosurveys in different locations from those previously identified, which also gave higher FOI estimates than previously recorded. Serosurveys performed in Peru, Vietnam and French Polynesia gave lower FOI estimates than those reported in Imai et al.,^4^ which is consistent with the highly heterogeneous nature of dengue transmission (given these were performed in different locations compared to those identified in Imai et al.^4^).

This study also identified countries where age-stratified dengue seroprevalence surveys were conducted for the first time since 2014. These include several endemic countries in Central and South America (Ecuador, Guadeloupe, Martinique, Colombia and Venezuela), as well as in Asia (Malaysia, Bangladesh, Taiwan, French Guiana and Saudi Arabia), and Africa (Burkina Faso, Tanzania, Kenya, Cameroon, Gabon, Djibouti, Nigeria and Madagascar). Of these, the ones from Ecuador, Guadeloupe, Martinique, French Guiana, Gabon, Djibouti, Madagascar and Burkina Faso are the first to be published in the respective countries.^15^ In line with our current understanding of global trends of dengue transmission, we find the highest FOI in Indonesia, India, Laos and Haiti, while the lowest in Taiwan and Saudi Arabia.

Through our literature review, we found two age-stratified dengue seroprevalence surveys conducted in Bangladesh, one in Dhaka city conducted in 2012^22^ and one conducted at the national level in 2022.^21^ These datasets report rather different age-dependent immunity profiles, with the national survey (performed 10 years after the one in Dhaka city) reporting a higher FOI estimate. Whilst the seroprevalence survey conducted in Dhaka city was a household serosurvey and included all age groups, the national survey was conducted in blood donors (and did not include individuals <18 years); differences in the surveyed populations could potentially explain the observed differences in the age-stratified seroprevalence profile, but overall this result suggests an increase in dengue transmission intensity across the country over the last 10 years.

Before this study, 13 published age-stratified seroprevalence surveys from Africa, specifically from Cameroon, Namibia, Nigeria, Kenya, Sudan and Tanzania had been identified.^15^ This review identified 8 additional age-stratified seroprevalence datasets from the African Region: three national surveys in Kenya, Djibouti (Horn of Africa) and Madagascar, two local surveys in Ougadougou (Burkina Faso), one in Osogbdo (Nigeria) and one in Zanzibar (Tanzania), three town-level serosurveys in the towns of Doualaa and Garoua (Cameroon) and Lambarene (Gabon), and a multi-regional survey in Tanzania (Buhigwe, Kalambo, Kilindi, Kinondoni, Kondoa, Kyela, Mvomero and Ukerewe). Amongst all the FOI estimates obtained across Africa, the serosurvey conducted in 2010 in Tanzania shows the highest FOI estimate (median 0.114 (95% CrI 0.034–0.254)), consistent with previous estimates obtained from blood donors.^81^

Notably, we found two age-stratified dengue seroprevalence surveys from Tanzania, one at a multi-regional level^80^ and one in Zanzibar,^81^ reporting rather different age-dependent susceptibility profiles. The multi-regional survey performed in 2018 suggests a relatively recent introduction of dengue (flat immunity profile), whilst the results reported in Zanzibar in 2010 show a clearly increasing age-dependent seroprevalence profile. These differences could reflect fundamental differences in transmission intensity, given that *Aedes aegypti* mosquitoes are urban vectors, and hence dengue is expected to circulate at higher intensity in urban settings, whilst the multi-regional seroprevalence profile likely captures the average immunity profile of the population at the national level, including in rural settings where dengue is likely to be circulating at lower intensities.

This study has a number of limitations, including the choice of relying on the Embase, Medline and Web of Science databases and the inclusion of papers written in the English language only, consistent with the criteria used in Imai et al.^4^ We also assumed time- and age-constant FOIs (and reporting rates), which capture long-term average transmission intensities that can be directly compared with the constant-in-time FOI maps generated in Cattarino et al.,^15^ which we are planning to update with the estimates generated in this literature review. Moreover, in this analysis we did not account for the different sensitivities and specificities of the different tests used across the serosurveys, as the in-house accuracy of the assays is rarely reported in published studies. Furthermore, we used age as the key driver of dengue exposure, but we did not account for other potential determinants including behavioural, occupational, socio-economic and environmental factors, which may have introduced some bias in the transmission intensity estimates generated in this study. Including these factors in future analyses would, however, require individual level (or highly disaggregated) data which are rarely available in published studies. Despite these limitations, this study has several strengths, including the use of well-established methods to estimate the FOI. Another strength of this study is the use of serological data as these data do not depend on the sensitivity of surveillance. This allows for within- and cross-country comparisons that can be used to investigate the fundamental environmental, demographic and climatic drivers of transmission underlying the observed heterogeneities. In this work, we have also tested the effect of alternative modelling assumptions on the FOI estimates, which is important to inform global comparisons of dengue transmission and burden.

In future work, it will be important to validate the serological-derived FOIs against those derived from age-stratified case-notification data, given the latter are collated in the countries where dengue is a notifiable disease (though these may not always be open access). Assessing the evidence of age-trends in case reporting and the model's ability to reconstruct age-dependencies is key to validating the use of case-notification data for estimating the FOI, which further strengthens the value of the serological data collated in this study.

In summary, the dengue FOI estimates generated in this study, along with the estimates obtained in previous analyses^15^ (Table S11), provide an up to date compendium of global FOI estimates obtained from age-stratified seroprevalence surveys, which will be useful to update the existing dengue burden estimates and to evaluate the impact of interventions to inform public health policy locally and globally.

### Conclusion

Age-stratified serological surveys provide key epidemiological data for estimating the transmission intensity of dengue, which in turn can be used to refine burden estimates and inform the optimal implementation of interventions such as vaccination. Our findings, alongside the serological data and FOI estimates published in Imai et al.^4^ and Cattarino et al.,^15^ summarize our current understanding of global heterogeneities in transmission intensity, as well as disparities in the geographical distribution of age-stratified serological surveys, thus highlighting regional gaps in arbovirus surveillance, which can help inform current and future efforts to strengthen surveillance capacity locally and globally.

## Contributors

Data curation: AV, ID.

Formal analysis: AV, ID.

Methodology: AV, ID.

Software: AV, ID.

Validation: AV, CMC.

Visualization: AV, CMC, ID.

Writing- Original Draft Preparation: AV, CMC, ID.

Writing. review & editing: AV, CMC, ID, BP, IR, NM.

Funding acquisition: ID, BP, IR, NM.

Supervision: ID.

Conceptualization: ID, BP, IR, NM.

All authors read and approved the final version of the manuscript.

## Data sharing statement

We provide the code to run the analysis presented in this manuscript, together with the age-stratified seroprevalence data on GitHub at the following link: https://github.com/AVicco/Dengue_FOI_review_code.

## Declaration of interests

ID declares consultancy work for the WHO, all the other authors declare no conflict of interest.
