# Supplementary material for: A scoping literature review of global dengue age-stratified seroprevalence data: estimating dengue force of infection in endemic countries
Source: eBioMedicine. 2024 May 7;104:105134. doi: 10.1016/j.ebiom.2024.105134 (PMC11096825; doi:10.1016/j.ebiom.2024.105134)
Supplement: Supplementary Figures and Tables [file mmc1.docx]

**Supplementary data**

**A scoping literature review of global dengue age-stratified seroprevalence: estimating dengue force of infection in endemic countries**

Anna Vicco, Clare McCormack, Belen Pedrique, Isabela Ribeiro, Gathsaurie Neelika Malavige, Ilaria Dorigatti

**Table of Contents**

Tables…………………………………………………………………………………………...2

Figures…………………………………………………………………………………………34

**Tables**

The literature search was performed on the 29^th^ of October 2023, the specific queries for each database are the following. In Web of Science the query was written as “(((((ALL=(seroprevalence)) OR ALL=(prevalence)) OR ALL=(seropositiv*)) OR ALL=(positiv*)) AND ALL=(dengue)) AND ALL=(sero*)”, while in Embase and Medline is reported in Tables S1 and S2.

**Table S1. Literature search performed in Embase.**

| **Step** | **Search Statement** |
| --- | --- |
| 1 | exp Dengue virus/ or exp dengue/ or exp severe dengue/ or dengue.mp. |
| 2 | seropositiv*.mp. |
| 3 | positiv*.mp. |
| 4 | seroprevalence.mp. or exp seroprevalence/ |
| 5 | sero*.mp. |
| 6 | 2 or 3 or 4 |
| 7 | 1 and 5 and 6 |
| 8 | limit 7 to yr=”2014 -Current” |

**Table S2. Literature search performed in Medline.**

| **Step** | **Search Statement** |
| --- | --- |
| 1 | dengue.mp. or exp Dengue/ or Severe Dengue/ or Dengue Virus/ |
| 2 | sero*.mp. |
| 3 | Prevalence/ or prevalence.mp. |
| 4 | seroprevalence.mp. or Seroepidemiologic Studies/ |
| 5 | positiv*.mp. |
| 6 | seropositiv*.mp. |
| 7 | 3 or 4 or 5 or 6 |
| 8 | 1 and 2 and 7 |
| 9 | limit 8 to yr=”2014 -Current” |

**Table S3. Region-specific FOI estimates obtained under model A.** Specifically, the table reports the FOI estimated under model A1 (binomial likelihood) and model A2 (beta-binomial likelihood) with constant FOI. The DIC (deviance information criterion) of the two models is also reported to compare them. The FOI is reported as median and 95% CrI.

| Country | Location | FOI (model A1) | FOI (model A2) | DIC (model A1) | DIC  (model A2) |
| --- | --- | --- | --- | --- | --- |
| Bangladesh | Dhaka city | 0.064(0.059, 0.069) | 0.045 (0.026, 0.071) | 100 | 61 |
| Brazil | Sau Paulo | 0.035 (0.033, 0.038) | 0.030 (0.013, 0.055) | 116 | 48 |
| Brazil | Fortaleza | 0.044 (0.037, 0.053) | 0.086 (0.024, 0.215) | 23 | 28 |
| Brazil | Tapera (village), in Itarema (municipality) in state of Ceara | 0.009 (0.007, 0.011) | 0.016 (0.005, 0.039) | 27 | 16 |
| Brazil | Recife (city) | 0.079 (0.074, 0.084) | 0.052 (0.028, 0.08) | 63 | 105 |
| Burkina Faso | Ougadougou | 0.063 (0.059, 0.066) | 0.054 (0.035, 0.076) | 54 | 83 |
| Cameroon | Garoua (town) | 0.009 (0.008, 0.011) | 0.014 (0.007, 0.033) | 46 | 29 |
| Cameroon | Doualaa (town) | 0.034 (0.03, 0.038) | 0.033 (0.016, 0.059) | 52 | 86 |
| Colombia | Quobdo | 0.041 (0.028, 0.06) | 0.062 (0.024, 0.139) | 15 | 21 |
| Colombia | Anapoima, Apulo, Buenaventura, Quibdo, Tumaco, Tierralta | 0.099 (0.091, 0.107) | 0.047 (0.020, 0.088) | 333 | 50 |
| Djibouti | Djibouti | 0.008 (0.007, 0.009) | 0.011 (0.004, 0.028) | 43 | 80 |
| Ecuador | Town of Quininde | 0.022 (0.019, 0.027) | 0.024 (0.015, 0.037) | 60 | 59 |
| France (carribean) | Guadeloupe and Martinique | 0.078 (0.07, 0.087) | 0.048 (0.026, 0.073) | 40 | 47 |
| Gabon | Lambarene (city) | 0.009 (0.007, 0.011) | 0.028 (0.009, 0.078) | 44 | 202 |
| India | Chennai | 0.134 (0.121, 0.148) | 0.088 (0.042, 0.148) | 49 | 109 |
| India | Delhi | 0.043 (0.036, 0.052) | 0.045 (0.020, 0.087) | 16 | 25 |
| India | Vaddu area | 0.021 (0.018, 0.023) | 0.024 (0.012, 0.047) | 47 | 50 |
| India | Pune | 0.078 (0.072, 0.084) | 0.063 (0.045, 0.084) | 63 | 91 |
| India | North (Delhi, Punjab and Uttar Pradesh) | 0.053 (0.05, 0.056) | 0.058 (0.022, 0.124) | 44 | 277 |
| India | NorthEast (Tripura, Meghalaya and Assam) | 0.002 (0.002, 0.003) | 0.019 (0.004, 0.071) | 34 | 26 |
| India | East (Bihar, West Bengal and Odisha) | 0.008 (0.007, 0.009) | 0.025 (0.007, 0.077) | 40 | 31 |
| India | West (Rajasthan, Madhya Pradesh and Maharashtra) | 0.048 (0.045, 0.051) | 0.054 (0.021, 0.114) | 42 | 25 |
| India | South (Andhra Pradesh, Karnataka and Tamil Nadu) | 0.078 (0.074, 0.082) | 0.069 (0.029, 0.136) | 43 | 101 |
| India | Kerala (urban Kanjiarappally) | 0.019 (0.017, 0.022) | 0.024 (0.012, 0.045) | 57 | 103 |
| India | Kerala ( rural as Koruthodu and Erumeli in Kottayam district) | 0.011 (0.01, 0.013) | 0.016 (0.008, 0.032) | 49 | 61 |
| India | Maharashtra (Vaddu area) 2014 | 0.017 (0.015, 0.021) | 0.055 (0.012, 0.165) | 26 | 14 |
| India | Maharashtra (Vaddu area) 2016 | 0.024 (0.021, 0.028) | 0.06 (0.015, 0.172) | 26 | 14 |
| India | Pune 2019 | 0.122 (0.113, 0.131) | 0.059 (0.031, 0.095) | 63 | 168 |
| India | Pune 2009 | 0.043 (0.039, 0.047) | 0.037 (0.021, 0.061) | 58 | 66 |
| India | Chennai, Tamil Nadu | 0.002 (0.001, 0.002) | 0.007 (0.002, 0.022) | 35 | 28 |
| India | Delhi | 0.136 (0.125, 0.148) | 0.129 (0.075, 0.196) | 58 | 39 |
| India | Kalayani | 0.035 (0.032, 0.039) | 0.057 (0.028, 0.111) | 61 | 50 |
| India | Wardha | 0.155 (0.138, 0.173) | 0.144 (0.085, 0.217) | 50 | 47 |
| India | Mumbai | 0.22 (0.193, 0.249) | 0.176 (0.104, 0.26) | 45 | 37 |
| India | Hyderabad | 0.116 (0.107, 0.126) | 0.117 (0.067, 0.184) | 62 | 73 |
| India | Bangalore | 0.141 (0.126, 0.158) | 0.125 (0.071, 0.193) | 52 | 43 |
| Indonesia | Denpasar Bali | 0.061 (0.055, 0.069) | 0.036 (0.011, 0.081) | 80 | 25 |
|  |  |  |  |  |  |
| Indonesia | national | 0.149 (0.142, 0.156) | 0.138 (0.111, 0.168) | 129 | 154 |
| Indonesia | Simpang Kiri (in Subulussalam, Nanggroe Aceh Darussalam) | 0.143 (0.11, 0.185) | 0.126 (0.084, 0.178) | 49 | 44 |
| Indonesia | Medan Denai (in Medan, Sumatera Utara) | 0.19 (0.147, 0.243) | 0.162 (0.108, 0.227) | 62 | 64 |
| Indonesia | Pauh (in Padang, Sumatera Barat) | 0.124 (0.095, 0.159) | 0.125 (0.085, 0.175) | 63 | 61 |
| Indonesia | Bungo Dani (in Bungo, Jambi) | 0.22 (0.165, 0.291) | 0.196 (0.135, 0.271) | 39 | 34 |
| Indonesia | Kalianda (in Lampung Selatan, Lampung) | 0.212 (0.161, 0.278) | 0.184 (0.125, 0.257) | 44 | 43 |
| Indonesia | Cikupa (in Tangerang, Banten) | 0.253 (0.19, 0.331) | 0.202 (0.135, 0.282) | 37 | 31 |
| Indonesia | Benda (in Tangerang, Banten) | 0.154 (0.117, 0.199) | 0.154 (0.106, 0.215) | 52 | 48 |
| Indonesia | Pesanggrahan (in Jakarta Selatan, Dki Jakarta) | 0.068 (0.05, 0.09) | 0.072 (0.048, 0.103) | 61 | 55 |
| Indonesia | Pulo Gadung (in Jakarta Timur, Dki Jakarta) | 0.127 (0.096, 0.166) | 0.115 (0.072, 0.168) | 48 | 55 |
| Indonesia | Kali Deres (in Jakarta Barat, Dki Jakarta) | 0.225 (0.17, 0.298) | 0.202 (0.14, 0.284) | 36 | 30 |
| Indonesia | Gunung Putri (in Bogor, Jawa Barat) | 0.126 (0.096, 0.162) | 0.135 (0.092, 0.19) | 50 | 45 |
| Indonesia | Banjaran (in Bandung, Jawa Barat) | 0.288 (0.216, 0.385) | 0.229 (0.162, 0.308) | 38 | 27 |
| Indonesia | Gunung Sari (in Cirebon, Jawa Barat) | 0.296 (0.22, 0.398) | 0.244 (0.168, 0.343) | 35 | 32 |
| Indonesia | Cikarang Utara (in Bekasi, Jawa Barat) | 0.047 (0.033, 0.064) | 0.054 (0.034, 0.082) | 63 | 55 |
| Indonesia | Bojongloa Kaler (in Bandung, Jawa Barat) | 0.137 (0.104, 0.179) | 0.128 (0.086, 0.18) | 48 | 47 |
| Indonesia | Bekasi Timur (in Bekasi, Jawa Barat) | 0.233 (0.175, 0.309) | 0.201 (0.14, 0.279) | 38 | 32 |
| Indonesia | Singaparna (in Tasikmalaya, Jawa Barat) | 0.132 (0.101, 0.17) | 0.127 (0.086, 0.179) | 54 | 53 |
| Indonesia | Trucuk (in Klaten, Jawa Tengah) | 0.258 (0.196, 0.336) | 0.192 (0.131, 0.265) | 46 | 40 |
| Indonesia | Pecangaan (in Jepara, Jawa Tengah) | 0.147 (0.114, 0.189) | 0.138 (0.096, 0.191) | 56 | 49 |
| Indonesia | Dukuhturi (in Tegal, Jawa Tengah) | 0.118 (0.09, 0.15) | 0.115 (0.08, 0.161) | 54 | 46 |
| Indonesia | Tegal Barat (in Tegal, Jawa Tengah) | 0.068 (0.05, 0.089) | 0.068 (0.043, 0.1) | 63 | 57 |
| Indonesia | Pulung (in Ponorogo, Jawa Timur) | 0.188 (0.146, 0.242) | 0.164 (0.114, 0.226) | 51 | 43 |
| Indonesia | Cluring (in Banyuwangi, Jawa Timur) | 0.336 (0.25, 0.451) | 0.252 (0.175, 0.347) | 36 | 27 |
| Indonesia | Ngoro (in Mojokerto, Jawa Timur) | 0.164 (0.127, 0.21) | 0.147 (0.101, 0.203) | 55 | 51 |
| Indonesia | Kalianget (in Sumenep, Jawa Timur) | 0.225 (0.17, 0.296) | 0.206 (0.145, 0.284) | 39 | 31 |
| Indonesia | Sawahan (in Surabaya, Jawa Timur) | 0.073 (0.054, 0.097) | 0.075 (0.049, 0.108) | 54 | 44 |
| Indonesia | Denpasar Selatan (in Denpasar, Bali) | 0.262 (0.2, 0.336) | 0.189 (0.128, 0.266) | 58 | 55 |
| Indonesia | Samarinda Ulu (in Samarinda, Kalimantan Timur) | 0.122 (0.092, 0.157) | 0.114 (0.079, 0.158) | 59 | 50 |
| Indonesia | Rantepao (in Toraja Utara, Sulawesi Selatan) | 0.196 (0.149, 0.255) | 0.167 (0.117, 0.228) | 49 | 42 |
| Indonesia | Kendari (in Kendari, Sulawesi Tenggara) | 0.157 (0.121, 0.199) | 0.133 (0.093, 0.182) | 59 | 50 |
| Kenya | national | 0.004 (0.004, 0.005) | 0.011 (0.003, 0.033) | 19 | 35 |
| Laos | Champasak | 0.216 (0.154, 0.306) | 0.119 (0.062, 0.205) | 20 | 17 |
| Laos | Savannakhet | 0.122 (0.095, 0.157) | 0.081 (0.044, 0.133) | 28 | 22 |
| Malaysia | Petaling district | 0.059 (0.052, 0.066) | 0.043 (0.025, 0.066) | 88 | 65 |
| Malaysia | Federal territory of Kuala, Lumpur, Perak, Kedah, Penang, Johor, Pahang, Kelantan and Sabah | 0.010 (0.008, 0.012) | 0.027 (0.009, 0.078) | 31 | 45 |
| Malaysia | Damansara Damai | 0.042 (0.031, 0.055) | 0.033 (0.010, 0.075) | 21 | 19 |
| Malaysia | Sungai Segamat | 0.029 (0.025, 0.033) | 0.027 (0.014, 0.046) | 37 | 39 |
| Malaysia | Peninsular Malaysia (urban) | 0.038 (0.036, 0.041) | 0.03 (0.014, 0.049) | 42 | 36 |
| Malaysia | Peninsular Malaysia (rural) | 0.034 (0.032, 0.036) | 0.026 (0.013, 0.045) | 42 | 40 |
| Malaysia | forest areas of Peninsular Malaysia | 0.01 (0.008, 0.013) | 0.023 (0.005, 0.074) | 25 | 28 |
| Mexico | state of Morelos | 0.052 (0.047, 0.057) | 0.044 (0.018, 0.087) | 229 | 48 |
| Mexico | Yucatan | 0.056 (0.053, 0.06) | 0.045 (0.020, 0.081) | 362 | 64 |
| Nigeria | Osogbo, Osun State | 0.018 (0.013, 0.026) | 0.035 (0.009, 0.102) | 18 | 10 |
| Pakistan | Lahore | 0.060 (0.050, 0.070) | 0.088 (0.033, 0.193) | 23 | 34 |
| Saudi Arabia | Jeddah | 0.017 (0.016, 0.018) | 0.020 (0.011, 0.037) | 26 | 32 |
| Saudi Arabia | Makkah | 0.009 (0.008, 0.011) | 0.021 (0.007, 0.058) | 31 | 32 |
| Saudi Arabia | Madinah | 0.004 (0.003, 0.006) | 0.016 (0.004, 0.05) | 25 | 14 |
| Saudi Arabia | Jeddah | 0.007 (0.006, 0.009) | 0.016 (0.004, 0.053) | 24 | 16 |
| Saudi Arabia | Jizan | 0.011 (0.009, 0.012) | 0.023 (0.007, 0.06) | 34 | 67 |
| Singapore | national | 0.020 (0.019, 0.021) | 0.020 (0.013, 0.030) | 299 | 102 |
| Singapore | blood service | 0.013 (0.011, 0.016) | 0.049 (0.012, 0.148) | 109 | 37 |
| Sri Lanka | city of Colombo | 0.155 (0.140, 0.171) | 0.149 (0.076, 0.248) | 55 | 51 |
| Sri Lanka | Ramalana community in Colombo district | 0.077 (0.061, 0.099) | 0.049 (0.026, 0.08) | 26 | 17 |
| Sri Lanka | Trincomalee district | 0.056 (0.047, 0.066) | 0.063 (0.026, 0.133) | 29 | 25 |
| Sri Lanka | Jaffna district | 0.032 (0.035, 0.037) | 0.046 (0.017, 0.107) | 30 | 18 |
| Sri Lanka | Kurunegala district | 0.012 (0.010, 0.014) | 0.03 (0.009, 0.093) | 34 | 19 |
| Sri Lanka | Matara district | 0.014 (0.011, 0.017) | 0.032 (0.009, 0.093) | 31 | 19 |
| Sri Lanka | Ratnapura district | 0.025 (0.021, 0.030) | 0.04 (0.014, 0.102) | 33 | 39 |
| Sri Lanka | Polonaruwa district | 0.022 (0.017, 0.028) | 0.04 (0.014, 0.104) | 29 | 20 |
| Sri Lanka | Gampha district | 0.025 (0.023, 0.028) | 0.041 (0.014, 0.104) | 39 | 24 |
| Sri Lanka | Kandy district | 0.013 (0.011, 0.015) | 0.031 (0.01, 0.093) | 33 | 27 |
| Sri Lanka | Badulla district | 0.011 (0.009, 0.014) | 0.029 (0.008, 0.09) | 31 | 18 |
| Sri Lanka | Sri Jayewadenpura | 0.068 (0.063, 0.073) | 0.059 (0.04, 0.08) | 82 | 79 |
| Taiwan | Nanzih district,Kaohsiung City | 0.001 (0.001, 0.001) | 0.004 (0.002, 0.011) | 31 | 37 |
| Taiwan | Sanmin district,Kaohsiung City | 0.005 (0.003, 0.007) | 0.009 (0.004, 0.019) | 20 | 32 |
| Taiwan | Taipei, Taoyuan, Tainan | 0.002 (0.001, 0.004) | 0.004 (0.002, 0.011) | 63 | 61 |
| Tanzania | Buhigwe, Kalambo,Kilindi, Kinondoni, Kondoa, Kyela, Mvomero and Ukerewe | 0.005 (0.004, 0.005) | 0.011 (0.003, 0.035) | 70 | 40 |
| Thailand | Ratchaburi province | 0.094 (0.074, 0.118) | 0.083 (0.035, 0.156) | 18 | 23 |
| Thailand | Ratchaburi province | 0.136 (0.127, 0.146) | 0.089 (0.050, 0.134) | 53 | 60 |
| Thailand | South (Narathiwat and Trang) | 0.07 (0.06, 0.081) | 0.079 (0.033, 0.168) | 22 | 15 |
| Thailand | Central (Ayutthaya and Lop Buri) | 0.07 (0.061, 0.081) | 0.045 (0.019, 0.085) | 28 | 15 |
| Thailand | Mukdahn | 0.112 (0.089, 0.143) | 0.095 (0.053, 0.163) | 20 | 23 |
| Thailand | Ubon | 0.119 (0.092, 0.155) | 0.131 (0.067, 0.237) | 17 | 16 |
| Vietnam | Nha Trang City, Nih Hoa district and Dien Khanh district (in Khanh Hoa province) | 0.008 (0.007, 0.009) | 0.012 (0.005, 0.029) | 67 | 156 |

**Table S4. Region-specific FOI estimates obtained under model B.** Specifically, the table reports the FOI estimated under model B1 (binomial likelihood) and model B2 (beta-binomial likelihood) with constant FOI and antibody decay. The DIC (deviance information criterion) of the two models is also reported to compare them. The FOI is reported as median and 95% CrI.

| Country | Location | FOI (model B1) | FOI (model B2) | DIC (model B1) | DIC (model B2) |
| --- | --- | --- | --- | --- | --- |
| Bangladesh | blood service | 0.111 (0.053, 0.19) | 0.133 (0.038, 0.288) | 1498 | 79 |
| Haiti | Gressier, Jacmel, Chabin | 0.292 (0.213, 0.414) | 0.209 (0.1, 0.351) | 1175 | 128 |
| India | Chennai | 0.067 (0.042, 0.101) | 0.072 (0.024, 0.172) | 1911 | 123 |
| Singapore | national | 0.005 (0.002, 0.01) | 0.034 (0.007, 0.143) | 360 | 81 |
| Tanzania | Zanzibar | 0.109 (0.066, 0.24) | 0.114 (0.034, 0.254) | 1613 | 59 |
| Venezuela | Cana de Azucar | 0.020 (0.012, 0.034) | 0.059 (0.015, 0.181) | 576 | 70 |

**Table S5. Region-specific FOI estimates obtained under model A1.** Specifically, the table reports the FOI estimated under model A1 (binomial likelihood) together withthe age at which we expect 50% seroprevalence and 70% seroprevalence and the average age at first infection. “NA” is used when the average age was above 100 years old. Each measure is reported as median and 95% CrI.

| Country | Location | FOI | Age_50%serop | | Age_70%serop | Age_first_infection |
| --- | --- | --- | --- | --- | --- | --- |
| Bangladesh | Dhaka city | 0.064 (0.059, 0.069) | 11 (10, 12) | 19 (18, 20) | | 16 (15, 17) |
| Brazil | Sau Paulo | 0.035 (0.033, 0.038) | 20 (18, 21) | 34 (31, 36) | | 28 (26, 30) |
| Brazil | Fortaleza | 0.044 (0.037, 0.053) | 16 (13, 18) | 27 (23, 32) | | 22 (19, 27) |
| Brazil | Tapera (village), in Itarema (municipality) in state of Ceara | 0.009 (0.007, 0.011) | 77 (64, 99) | NA | | NA |
| Brazil | Recife (city) | 0.079 (0.074, 0.084) | 9 (8, 9) | 15 (14, 16) | | 13 (12, 13) |
| Burkina Faso | Ougadougou | 0.063 0.059, 0.066) | 11 (11, 12) | 19 (18, 20) | | 16 (15, 17) |
| Cameroon | Garoua (town) | 0.009 (0.008, 0.011) | 74 (62, 89) | NA | | NA |
| Cameroon | Doualaa (town) | 0.034 (0.03, 0.038) | 21 (19, 23) | 36 (32, 40) | | 30 (27, 33) |
| Colombia | Anapoima, Apulo, Buenaventura, Quibdo, Tumaco, Tierralta | 0.099 (0.091, 0.107) | 7 (7, 7) | 12 (11, 13) | | 10 (9, 11) |
| Colombia | Quobdo | 0.041 (0.028, 0.06) | 17 (11, 25) | 29 (20 , 44 ) | | 24 (16, 36) |
| Djibouti | Djibouti | 0.008 (0.007, 0.009) | 86 (75, 95) | NA | | NA |
| Ecuador | Town of Quininde | 0.022 (0.019, 0.027) | 32 (27, 39) | 55 (46, 67) | | 46 (38, 56) |
| French carribean | Guadeloupe and Martinique | 0.078 0.07, 0.087) | 9 (8, 10) | 15 (14, 17) | | 13 (12, 14) |
| Gabon | Lambarene (city) | 0.009 (0.007, 0.011) | 79 (64, 95) | NA | | NA |
| India | Delhi | 0.043 (0.036, 0.052) | 16 (14, 20) | 28 (24, 34) | | 23 (20, 28) |
| India | Denpasar Bali | 0.061 (0.055, 0.069) | 11 (10, 13) | 20 (18, 22) | | 17 (15, 18) |
| India | Pune | 0.078 (0.072, 0.084) | 9 (8, 10) | 16 (14, 17) | | 13 (12, 14) |
| India | Vadu area | 0.021 (0.018, 0.023) | 34 (30, 37) | 59 (53, 64) | | 49 (44, 53) |
| India | Chennai | 0.134 (0.121, 0.148) | 5 (5, 6) | 9 (8, 10) | | 8 (7, 8) |
| India | North (Delhi, Punjab and Uttar Pradesh) | 0.053 (0.05, 0.056) | 13 (13, 14) | 23 (22, 24) | | 19 (18, 20) |
| India | NorthEast (Tripura, Meghalaya and Assam) | 0.002 (0.002, 0.003) | 337 (275, 438) | NA | | NA |
| India | East (Bihar, West Bengal and Odisha) | 0.008 (0.007, 0.009) | 85 (75, 95) | NA | | NA |
| India | West (Rajasthan, Madhya Pradesh and Maharashtra) | 0.048 (0.045, 0.051) | 14 (14, 15) | 25 (24, 27) | | 21 (20, 22) |
| India | South (Andhra Pradesh, Karnataka and Tamil Nadu) | 0.078 (0.074, 0.082) | 9 (8, 9) | 15 (15, 16) | | 13 (12, 13) |
| India | Kerala (urban Kanjiarappally) | 0.019 (0.017, 0.022) | 36 (31, 41) | 62 (55, 72) | | 52 (45, 60) |
| India | Kerala (rural as Koruthodu and Erumeli in Kottayam district) | 0.011 (0.01, 0.013) | 60 (51, 69) | 105 (89, 120) | | 87 (74, 99) |
| India | Maharashtra (Vadu area) 2014 | 0.017 (0.015, 0.021) | 40 (33, 46) | 69 (57, 80) | | 57 (48, 66) |
| India | Maharashtra (Vadu area) 2016 | 0.024 (0.021, 0.028) | 29 (25, 33) | 50 (44, 58) | | 41 (36, 48) |
| India | Pune 2019 | 0.122 (0.113, 0.131) | 6 (5, 6) | 10 (9, 11) | | 8 (8, 9) |
| India | Pune 2009 | 0.043 (0.039, 0.047) | 16 (15, 18) | 28 (25, 30) | | 23 (21, 25) |
| India | Chennai, Tamil Nadu | 0.002 (0.001, 0.002) | 399 (285, 613) | NA | | NA |
| India | Delhi | 0.136 (0.125, 0.148) | 5 (5, 6) | 9 (8, 10) | | 7 (7, 8) |
| India | Kalayani | 0.035 (0.032, 0.039) | 20 (18, 22) | 34 (31, 38) | | 28 (26, 32) |
| India | Wardha | 0.155 (0.138, 0.173) | 4 (4, 5) | 8 (7, 8) | | 6 (6, 7) |
| India | Mumbai | 0.22 (0.193, 0.249) | 3 (3, 4) | 5 (5, 6) | | 5 (4, 5) |
| India | Hyderabad | 0.116 (0.107, 0.126) | 6 (6, 6) | 10 (10, 11) | | 9 (8, 9) |
| India | Bangalore | 0.141 (0.126, 0.158) | 5 (4, 5) | 9 (8, 9) | | 7 (6, 8) |
| Indonesia | national | 0.144 (0.138, 0.151) | 5 (5, 5) | 8 (8, 9) | | 7 (7, 7) |
| Indonesia | Simpang Kiri (in Subulussalam, Nanggroe Aceh Darussalam) | 0.143 (0.11, 0.185) | 5 (4, 6) | 8 (6, 11) | | 7 (5, 9) |
| Indonesia | Medan Denai (in Medan, Sumatera Utara) | 0.19 (0.147, 0.243) | 4 (3, 5) | 7 (5, 8) | | 5 (4, 7) |
| Indonesia | Pauh (in Padang, Sumatera Barat) | 0.124 (0.095, 0.159) | 5 (5, 7) | 10 (8, 12) | | 8 (7, 10) |
| Indonesia | Bungo Dani (in Bungo, Jambi) | 0.22 (0.165, 0.291) | 3 (2, 4) | 5 (4, 8) | | 5 (3, 6) |
| Indonesia | Kalianda (in Lampung Selatan, Lampung) | 0.212 (0.161, 0.278) | 3 (3, 4) | 6 (4, 7) | | 5 (4, 6) |
| Indonesia | Cikupa (in Tangerang, Banten) | 0.253 (0.19, 0.331) | 3 (2, 4) | 5 (4, 6) | | 4 (3, 5) |
| Indonesia | Benda (in Tangerang, Banten) | 0.154 (0.117, 0.199) | 5 (4, 6) | 8 (6, 10) | | 7 (5, 8) |
| Indonesia | Pesanggrahan (in Jakarta Selatan, Dki Jakarta) | 0.068 (0.05, 0.09) | 10 (8, 14) | 17 (13, 24) | | 14 (11, 20) |
| Indonesia | Pulo Gadung (in Jakarta Timur, Dki Jakarta) | 0.127 (0.096, 0.166) | 5 (4, 7) | 9 (7, 12) | | 8 (6, 10) |
| Indonesia | Kali Deres (in Jakarta Barat, Dki Jakarta) | 0.225 (0.17, 0.298) | 3 (2, 4) | 5 (4, 7) | | 4 (3, 6) |
| Indonesia | Gunung Putri (in Bogor, Jawa Barat) | 0.126 (0.096, 0.162) | 6 (5, 7) | 10 (8, 12) | | 8 (7, 10) |
| Indonesia | Banjaran (in Bandung, Jawa Barat) | 0.288 (0.216, 0.385) | 2 (2, 3) | 4 (3, 5) | | 3 (2, 5) |
| Indonesia | Gunung Sari (in Cirebon, Jawa Barat) | 0.296 (0.22, 0.398) | 2 (2, 3) | 4 (3, 6) | | 3 (2, 5) |
| Indonesia | Cikarang Utara (in Bekasi, Jawa Barat) | 0.047 (0.033, 0.064) | 14 (11, 19) | 25 (19, 33) | | 21 (15, 28) |
| Indonesia | Bojongloa Kaler (in Bandung, Jawa Barat) | 0.137 (0.104, 0.179) | 5 (4, 7) | 9 (7, 12) | | 7 (5, 10) |
| Indonesia | Bekasi Timur (in Bekasi, Jawa Barat) | 0.233 (0.175, 0.309) | 3 (2, 4) | 5 (4, 7) | | 4 (3, 5) |
| Indonesia | Singaparna (in Tasikmalaya, Jawa Barat) | 0.132 (0.101, 0.17) | 5 (4, 7) | 9 (7, 12) | | 8 (6, 10) |
| Indonesia | Trucuk (in Klaten, Jawa Tengah) | 0.258 (0.196, 0.336) | 3 (2, 3) | 5 (4, 6) | | 4 (3, 5) |
| Indonesia | Pecangaan (in Jepara, Jawa Tengah) | 0.147 (0.114, 0.189) | 5 (4, 6) | 8 (6, 11) | | 7 (5, 9) |
| Indonesia | Dukuhturi (in Tegal, Jawa Tengah) | 0.118 (0.09, 0.15) | 6 (5, 7) | 10 (8, 13) | | 8 (7, 10) |
| Indonesia | Tegal Barat (in Tegal, Jawa Tengah) | 0.068 (0.05, 0.089) | 10 (8, 14) | 18 (14, 24) | | 15 (11, 20) |
| Indonesia | Pulung (in Ponorogo, Jawa Timur) | 0.188 (0.146, 0.242) | 4 (3, 5) | 6 (5, 8) | | 5 (4, 7) |
| Indonesia | Cluring (in Banyuwangi, Jawa Timur) | 0.336 (0.25, 0.451) | 2 (2, 3) | 4 (3, 5) | | 3 (2, 4) |
| Indonesia | Ngoro (in Mojokerto, Jawa Timur) | 0.164 (0.127, 0.21) | 4 (3, 6) | 8 (6, 10) | | 6 (5, 8) |
| Indonesia | Kalianget (in Sumenep, Jawa Timur) | 0.225 (0.17, 0.296) | 3 (2, 4) | 5 (4, 7) | | 4 (3, 6) |
| Indonesia | Sawahan (in Surabaya, Jawa Timur) | 0.073 (0.054, 0.097) | 9 (7, 13) | 16 (12, 22) | | 14 (10, 18) |
| Indonesia | Denpasar Selatan (in Denpasar, Bali) | 0.262 (0.2, 0.336) | 3 (2, 3) | 5 (4, 6) | | 4 (3, 5) |
| Indonesia | Samarinda Ulu (in Samarinda, Kalimantan Timur) | 0.122 (0.092, 0.157) | 6 (4, 8) | 10 (8, 13) | | 8 (6, 11) |
| Indonesia | Rantepao (in Toraja Utara, Sulawesi Selatan) | 0.196 (0.149, 0.255) | 4 (3, 4) | 6 (5, 8) | | 5 (4, 6) |
| Indonesia | Kendari (in Kendari, Sulawesi Tenggara) | 0.157 (0.121, 0.199) | 4 (3, 6) | 8 (6, 11) | | 6 (5, 9) |
| Kenya | national | 0.004 (0.004, 0.005) | NA | NA | | NA |
| Laos | Champasak | 0.216 (0.154, 0.306) | 3 (2, 4) | 6 (4, 7) | | 5 (3, 6) |
| Laos | Savannakhet | 0.122 (0.095, 0.157) | 6 (4, 7) | 10 (8, 12) | | 8 (6, 10) |
| Malaysia | Federal territory of Kuala Lumpur, Perak, Kedah, Penang, Johor, Pahang, Kelantan and Sabah | 0.01 (0.008, 0.012) | 70 (61, 80) | NA | | NA |
| Malaysia | Damansara Damai | 0.042 (0.031, 0.055) | 17 (13, 21) | 29 (23, 37) | | 24 (19, 31) |
| Malaysia | Petaling district | 0.059 (0.052, 0.066) | 12 (11, 13) | 21 (18, 22) | | 17 (15, 19) |
| Malaysia | Sungai Segamat | 0.029 (0.025, 0.033) | 24 (21, 29) | 42 (36, 51) | | 35 (30, 42) |
| Malaysia | Peninsular Malaysia (urban) | 0.038 (0.036, 0.041) | 18 (17, 19) | 31 (29, 33) | | 26 (24, 28) |
| Malaysia | Peninsular Malaysia (rural) | 0.034 (0.032, 0.036) | 21 (19, 22) | 36 (33, 38) | | 30 (28, 32) |
| Malaysia | forest areas of Peninsular Malaysia | 0.01 (0.008, 0.013) | 67 (54, 81) | NA | | NA |
| Mexico | Yucatan | 0.056 (0.053, 0.06) | 12 (12, 13) | 21 (20, 23) | | 18 (17, 19) |
| Mexico | state of Morelos | 0.052 (0.047, 0.057) | 13 (12, 14) | 23 (22, 25) | | 19 (18, 21) |
| Nigeria | Osogbo, Osun State | 0.018 (0.013, 0.026) | 37 (28, 60) | 65 (48, 104) | | 54 (40, 86) |
| Pakistan | Lahore | 0.06 (0.05, 0.07) | 12 (10, 13) | 20 (17, 23) | | 17 (14, 19) |
| Saudi arabia | Jeddah | 0.017 (0.016, 0.018) | 41 (38, 43) | 71 (66, 75) | | 59 (55, 63) |
| Saudi Arabia | Makkah | 0.009 (0.008, 0.011) | 77 (66, 91) | NA | | NA |
| Saudi Arabia | Madinah | 0.004 (0.003, 0.006) | NA | NA | | NA |
| Saudi Arabia | Jeddah | 0.007 (0.006, 0.009) | 94 (81, 108) | NA | | NA |
| Saudi Arabia | Jizan | 0.011 (0.009, 0.012) | 65 (58, 77) | 114 (101, 134) | | 94 (84, 111) |
| Singapore | blood service | 0.013 (0.011, 0.016) | 52 (45, 61) | 89 (78, 106) | | 74 (65, 88) |
| Singapore | national | 0.02 (0.019, 0.021) | 34 (33, 36) | 59 (57, 63) | | 49 (47, 52) |
| Sri Lanka | city of Colombo | 0.155 (0.14, 0.171) | 4 (4, 5) | 8 (7, 9) | | 6 (6, 7) |
| Sri Lanka | Ramalana community in Colombo district | 0.077 (0.061, 0.099) | 9 (7, 11) | 16 (12, 20) | | 13 (10, 16) |
| Sri Lanka | Trincomalee district | 0.056 (0.047, 0.066) | 12 (11, 15) | 22 (18, 26) | | 18 (15, 21) |
| Sri Lanka | Jaffna district | 0.032 (0.035, 0.037) | 23 (19, 27) | 39 (33, 47) | | 33 (27, 39) |
| Sri Lanka | Kurunegala district | 0.012 (0.010, 0.014) | 59 (50, 71) | 102 (87, 123) | | 85 (72, 102) |
| Sri Lanka | Matara district | 0.014 (0.011, 0.017) | 51 (41, 63) | 88 (72, 109) | | 73 (60, 91) |
| Sri Lanka | Ratnapura district | 0.025 (0.021, 0.030) | 27 (23, 32) | 48 (41, 56) | | 40 (34, 47) |
| Sri Lanka | Polonaruwa district | 0.022 (0.017, 0.028) | 31 (25, 40) | 54 (44, 70) | | 45 (36, 58) |
| Sri Lanka | Gampha district | 0.025 (0.023, 0.028) | 27 (25, 30) | 47 (43, 52) | | 39 (36, 43) |
| Sri Lanka | Kandy district | 0.013 (0.011, 0.015) | 54 (45, 65) | 94 (79, 113) | | 78 (66, 94) |
| Sri Lanka | Kandy district | 0.011 (0.009, 0.014) | 64 (51, 81) | 111 (88, 141) | | 92 (73, 117) |
| Sri Lanka | Sri Jayewadenpura | 0.068 (0.063, 0.073) | 10 (9, 11) | 18 (16, 19) | | 15 (14, 16) |
| Taiwan | Taipei, Taoyuan, Tainan | 0.001 (0.001, 0.001) | NA | NA | | NA |
| Taiwan | Nanzih district, Kaohsiung City | 0.005 (0.003, 0.007) | NA | NA | | NA |
| Taiwan | Sanmin district, Kaohsiung City | 0.002 (0.001, 0.004) | NA | NA | | NA |
| Tanzania | Buhigwe, Kalambo, Kilindi, Kinondoni, Kondoa, Kyela, Mvomero and Ukerewen | 0.005 (0.004, 0.005) | NA | NA | | NA |
| Thailand | Ratchaburi province | 0.094 (0.074, 0.118) | 7 (6, 9) | 13 (10, 16) | | 11 (8, 13) |
| Thailand | Ratchaburi province | 0.136 (0.127, 0.146) | 5 (5, 5) | 9 (8, 9) | | 7 (7, 8) |
| Thailand | South (Narathiwat and Trang) | 0.07 (0.06, 0.081) | 10 (9, 11) | 17 (15, 20) | | 14 (12, 16) |
| Thailand | Central (Ayutthaya and Lop Buri) | 0.07 (0.061, 0.081) | 10 (9, 11) | 17 (15, 19) | | 14 (13, 16) |
| Thailand | Mukdahn | 0.112 (0.089, 0.143) | 6 (5, 8) | 11 (8, 13) | | 9 (7, 11) |
| Thailand | Ubon | 0.119 (0.092, 0.155) | 6 (5, 8) | 10 (8, 14) | | 8 (7, 12) |
| Vietnam | Nha Trang City, Nih Hoa district and Dien Khanh district (in Khanh Hoa province) | 0.008 (0.007, 0.009) | 87 (79, 95) | NA | | NA |

**Table S6. Region-specific FOI estimates obtained under model A2.** Specifically, the table reports the FOI estimated under model A2 (beta-binomial likelihood) together with the age at which we expect 50% seroprevalence and 70% seroprevalence and the average age at first infection. The table also reports the estimates of the over-dispersion parameter phi obtained under model A2. “NA” is used when the average age was above 100 years old. Each measure is reported as median and 95% CrI.

| Country | Location | FOI | Age_50%serop | | | Age_70%serop | Age_first_infection | | Phi | |  |
| --- | --- | --- | --- | --- | --- | --- | --- | --- | --- | --- | --- |
| Bangladesh | Dhaka city | 0.045 (0.026, 0.071) | | 16 (10, 26) | 27 (17, 44) | | | 22 (14, 37) | | 0.243 (0.123, 0.483) | |
| Brazil | Sau Paulo | 0.03 (0.013, 0.055) | | 21 (12, 49) | 37 (20, 85) | | | 31 (17, 70) | | 0.285 (0.136, 0.571) | |
| Brazil | Fortaleza | 0.086 (0.024, 0.215) | | 8 (4, 29) | 13 (6, 50) | | | 11 (5, 42) | | 0.395 (0.17, 0.759) | |
| Brazil | Tapera (village), in Itarema (municipality) in state of Ceara | 0.016 (0.005, 0.039) | | 45 (18, 112) | 78 (32, 194) | | | 65 (26, 161) | | 0.315 (0.141, 0.643) | |
| Brazil | Recife (city) | 0.052 (0.028, 0.08) | | 13 (9, 24) | 23 (16, 42) | | | 19 (14, 35) | | 0.248 (0.12, 0.516) | |
| Burkina Faso | Ougadougou | 0.054 (0.035, 0.076) | | 13 (9, 19) | 22 (16, 33) | | | 18 (13, 27) | | 0.17 (0.089, 0.342) | |
| Cameroon | Garoua (town) | 0.014 (0.007, 0.033) | | 54 (28, 96) | 93 (48, 167) | | | 77 (40, 139) | | 0.249 (0.117, 0.516) | |
| Cameroon | Doualaa (town) | 0.033 (0.016, 0.059) | | 23 (13, 35) | 39 (23, 61) | | | 32 (19, 51) | | 0.253 (0.127, 0.501) | |
| Colombia | Anapoima, Apulo, Buenaventura, Quibdo, Tumaco, Tierralta | 0.047 (0.02, 0.088) | | 14 (8, 33) | 24 (14, 57) | | | 20 (11, 48) | | 0.381 (0.185, 0.681) | |
| Colombia | Quobdo | 0.062 (0.024, 0.139) | | 12 (5, 26) | 21 (9, 45) | | | 18 (8, 37) | | 0.315 (0.147, 0.635) | |
| Djibouti | Djibouti | 0.011 (0.004, 0.028) | | 61 (29, 144) | 106 (50, 249) | | | 88 (42, 207) | | 0.305 (0.142, 0.609) | |
| Ecuador | Town of Quininde | 0.024 (0.015, 0.037) | | 27 (19, 40) | 47 (33, 70) | | | 39 (28, 58) | | 0.189 (0.104, 0.35) | |
| French carribean | Guadeloupe and Martinique | 0.048 (0.026, 0.073) | | 14 (9, 23) | 25 (15, 40) | | | 20 (13, 33) | | 0.261 (0.124, 0.544) | |
| Gabon | Lambarene (city) | 0.028 (0.009, 0.078) | | 26 (9, 79) | 45 (16, 137) | | | 37 (14, 114) | | 0.387 (0.198, 0.67) | |
| India | Delhi | 0.045 (0.02, 0.087) | | 16 (8, 31) | 28 (14, 53) | | | 23 (12, 44) | | 0.311 (0.143, 0.641) | |
| India | Pune | 0.063 (0.045, 0.084) | | 11 (8, 16) | 19 (15, 27) | | | 16 (12, 23) | | 0.142 (0.078, 0.271) | |
| India | Vadu area | 0.024 (0.012, 0.047) | | 29 (18, 54) | 51 (31, 94) | | | 42 (26, 78) | | 0.238 (0.118, 0.476) | |
| India | Chennai | 0.088 (0.042, 0.148) | | 8 (5, 13) | 13 (8, 23) | | | 11 (7, 19) | | 0.335 (0.161, 0.625) | |
| India | North (Delhi, Punjab and Uttar Pradesh) | 0.058 (0.022, 0.124) | | 11 (6, 32) | 19 (10, 55) | | | 16 (8, 46) | | 0.318 (0.146, 0.634) | |
| India | NorthEast (Tripura, Meghalaya and Assam) | 0.019 (0.004, 0.071) | | 33 (9, 163) | 58 (15, 282) | | | 48 (13, 235) | | 0.434 (0.175, 0.776) | |
| India | East (Bihar, West Bengal and Odisha) | 0.025 (0.007, 0.077) | | 27 (8, 77) | 47 (15, 134) | | | 39 (12, 112) | | 0.363 (0.154, 0.705) | |
| India | West (Rajasthan, Madhya Pradesh and Maharashtra) | 0.054 (0.021, 0.114) | | 13 (6, 32) | 22 (10, 55) | | | 19 (9, 46) | | 0.295 (0.137, 0.617) | |
| India | South (Andhra Pradesh, Karnataka and Tamil Nadu) | 0.069 (0.029, 0.136) | | 10 (5, 22) | 17 (9, 39) | | | 14 (7, 32) | | 0.297 (0.135, 0.617) | |
| India | Kerala (urban Kanjiarappally) | 0.024 (0.012, 0.045) | | 30 (13, 54) | 51 (23, 94) | | | 43 (19, 78) | | 0.252 (0.131, 0.477) | |
| India | Kerala (rural as Koruthodu and Erumeli in Kottayam district) | 0.016 (0.008, 0.032) | | 44 (22, 91) | 76 (38, 157) | | | 63 (31, 131) | | 0.25 (0.126, 0.495) | |
| India | Maharashtra (Vadu area) 2014 | 0.055 (0.012, 0.165) | | 12 (3, 45) | 21 (6, 78) | | | 18 (5, 65) | | 0.412 (0.172, 0.774) | |
| India | Maharashtra (Vadu area) 2016 | 0.06 (0.015, 0.172) | | 10 (4, 44) | 17 (6, 76) | | | 14 (5, 63) | | 0.4 (0.169, 0.767) | |
| India | Pune 2019 | 0.059 (0.031, 0.095) | | 12 (8, 24) | 21 (14, 42) | | | 17 (11, 35) | | 0.295 (0.144, 0.562) | |
| India | Pune 2009 | 0.037 (0.021, 0.061) | | 19 (13, 29) | 32 (22, 51) | | | 27 (18, 42) | | 0.222 (0.111, 0.446) | |
| India | Chennai, Tamil Nadu | 0.007 (0.002, 0.022) | | NA | NA | | | NA | | 0.348 (0.147, 0.693) | |
| India | Delhi | 0.129 (0.075, 0.196) | | 5 (3, 9) | 9 (6, 16) | | | 8 (5, 13) | | 0.212 (0.105, 0.442) | |
| India | Kalayani | 0.057 (0.028, 0.111) | | 11 (6, 21) | 20 (11, 36) | | | 16 (9, 30) | | 0.233 (0.113, 0.474) | |
| India | Wardha | 0.144 (0.085, 0.217) | | 5 (3, 7) | 8 (6, 13) | | | 7 (5, 11) | | 0.23 (0.114, 0.467) | |
| India | Mumbai | 0.176 (0.104, 0.26) | | 4 (3, 6) | 7 (5, 11) | | | 6 (4, 9) | | 0.245 (0.121, 0.5) | |
| India | Hyderabad | 0.117 (0.067, 0.184) | | 6 (4, 9) | 10 (6, 16) | | | 8 (5, 14) | | 0.223 (0.112, 0.447) | |
| India | Bangalore | 0.125 (0.071, 0.193) | | 6 (4, 10) | 10 (6, 17) | | | 8 (5, 14) | | 0.224 (0.111, 0.451) | |
| Indonesia | Denpasar Bali | 0.036 (0.011, 0.081) | | 20 (9, 53) | 35 (15, 92) | | | 29 (13, 76) | | 0.386 (0.166, 0.745) | |
|  |  |  | |  |  | | |  | |  | |
| Indonesia | national | 0.138 (0.111, 0.168) | | 5 (4, 6) | 9 (7, 11) | | | 7 (6, 9) | | 0.103 (0.062, 0.185) | |
| Indonesia | Simpang Kiri (in Subulussalam, Nanggroe Aceh Darussalam) | 0.126 (0.084, 0.178) | | 6 (3, 9) | 10 (6, 15) | | | 8 (5, 12) | | 0.235 (0.123, 0.422) | |
| Indonesia | Medan Denai (in Medan, Sumatera Utara) | 0.162 (0.108, 0.227) | | 4 (3, 6) | 8 (5, 11) | | | 6 (5, 9) | | 0.297 (0.154, 0.511) | |
| Indonesia | Pauh (in Padang, Sumatera Barat) | 0.125 (0.085, 0.175) | | 5 (4, 8) | 9 (7, 13) | | | 8 (6, 11) | | 0.239 (0.128, 0.415) | |
| Indonesia | Bungo Dani (in Bungo, Jambi) | 0.196 (0.135, 0.271) | | 3 (2, 5) | 6 (4, 9) | | | 5 (4, 7) | | 0.262 (0.133, 0.481) | |
| Indonesia | Kalianda (in Lampung Selatan, Lampung) | 0.184 (0.125, 0.257) | | 4 (3, 5) | 7 (5, 10) | | | 6 (4, 8) | | 0.319 (0.158, 0.574) | |
| Indonesia | Cikupa (in Tangerang, Banten) | 0.202 (0.135, 0.282) | | 3 (3, 5) | 6 (4, 8) | | | 5 (4, 7) | | 0.28 (0.134, 0.536) | |
| Indonesia | Benda (in Tangerang, Banten) | 0.154 (0.106, 0.215) | | 4 (3, 7) | 8 (6, 12) | | | 6 (5, 10) | | 0.239 (0.125, 0.426) | |
| Indonesia | Pesanggrahan (in Jakarta Selatan, Dki Jakarta) | 0.072 (0.048, 0.103) | | 10 (7, 16) | 17 (12, 27) | | | 14 (10, 23) | | 0.2 (0.112, 0.345) | |
| Indonesia | Pulo Gadung (in Jakarta Timur, Dki Jakarta) | 0.115 (0.072, 0.168) | | 6 (4, 10) | 10 (7, 18) | | | 9 (6, 15) | | 0.407 (0.206, 0.659) | |
| Indonesia | Kali Deres (in Jakarta Barat, Dki Jakarta) | 0.202 (0.14, 0.284) | | 3 (2, 5) | 6 (4, 9) | | | 5 (3, 8) | | 0.262 (0.13, 0.484) | |
| Indonesia | Gunung Putri (in Bogor, Jawa Barat) | 0.135 (0.092, 0.19) | | 5 (4, 7) | 9 (6, 12) | | | 7 (5, 10) | | 0.22 (0.118, 0.402) | |
| Indonesia | Banjaran (in Bandung, Jawa Barat) | 0.229 (0.162, 0.308) | | 3 (2, 5) | 5 (4, 8) | | | 4 (3, 7) | | 0.209 (0.11, 0.393) | |
| Indonesia | Gunung Sari (in Cirebon, Jawa Barat) | 0.244 (0.168, 0.343) | | 3 (2, 4) | 5 (4, 7) | | | 4 (3, 6) | | 0.296 (0.147, 0.54) | |
| Indonesia | Cikarang Utara (in Bekasi, Jawa Barat) | 0.054 (0.034, 0.082) | | 13 (9, 19) | 23 (16, 34) | | | 19 (13, 28) | | 0.198 (0.11, 0.344) | |
| Indonesia | Bojongloa Kaler (in Bandung, Jawa Barat) | 0.128 (0.086, 0.18) | | 6 (4, 8) | 10 (7, 13) | | | 8 (6, 11) | | 0.282 (0.146, 0.498) | |
| Indonesia | Bekasi Timur (in Bekasi, Jawa Barat) | 0.201 (0.14, 0.279) | | 3 (3, 5) | 6 (4, 8) | | | 5 (4, 7) | | 0.244 (0.126, 0.452) | |
| Indonesia | Singaparna (in Tasikmalaya, Jawa Barat) | 0.127 (0.086, 0.179) | | 5 (4, 9) | 9 (7, 15) | | | 8 (6, 13) | | 0.272 (0.142, 0.488) | |
| Indonesia | Trucuk (in Klaten, Jawa Tengah) | 0.192 (0.131, 0.265) | | 4 (3, 5) | 6 (4, 9) | | | 5 (4, 8) | | 0.266 (0.132, 0.506) | |
| Indonesia | Pecangaan (in Jepara, Jawa Tengah) | 0.138 (0.096, 0.191) | | 5 (4, 7) | 9 (6, 13) | | | 7 (5, 11) | | 0.205 (0.112, 0.365) | |
| Indonesia | Dukuhturi (in Tegal, Jawa Tengah) | 0.115 (0.08, 0.161) | | 6 (4, 8) | 10 (7, 14) | | | 9 (6, 12) | | 0.212 (0.112, 0.387) | |
| Indonesia | Tegal Barat (in Tegal, Jawa Tengah) | 0.068 (0.043, 0.1) | | 11 (7, 15) | 18 (12, 27) | | | 15 (10, 22) | | 0.213 (0.116, 0.376) | |
| Indonesia | Pulung (in Ponorogo, Jawa Timur) | 0.164 (0.114, 0.226) | | 4 (3, 6) | 8 (5, 10) | | | 6 (4, 9) | | 0.221 (0.116, 0.407) | |
| Indonesia | Cluring (in Banyuwangi, Jawa Timur) | 0.252 (0.175, 0.347) | | 3 (2, 4) | 5 (4, 7) | | | 4 (3, 6) | | 0.243 (0.122, 0.463) | |
| Indonesia | Ngoro (in Mojokerto, Jawa Timur) | 0.147 (0.101, 0.203) | | 5 (3, 7) | 8 (6, 12) | | | 7 (5, 10) | | 0.224 (0.12, 0.404) | |
| Indonesia | Kalianget (in Sumenep, Jawa Timur) | 0.206 (0.145, 0.284) | | 3 (2, 4) | 6 (4, 8) | | | 5 (4, 6) | | 0.236 (0.121, 0.445) | |
| Indonesia | Sawahan (in Surabaya, Jawa Timur) | 0.075 (0.049, 0.108) | | 9 (6, 13) | 16 (11, 23) | | | 14 (9, 19) | | 0.193 (0.104, 0.352) | |
| Indonesia | Denpasar Selatan (in Denpasar, Bali) | 0.189 (0.128, 0.266) | | 4 (3, 5) | 6 (5, 9) | | | 5 (4, 7) | | 0.305 (0.15, 0.549) | |
| Indonesia | Samarinda Ulu (in Samarinda, Kalimantan Timur) | 0.114 (0.079, 0.158) | | 6 (4, 9) | 11 (7, 15) | | | 9 (6, 13) | | 0.178 (0.1, 0.313) | |
| Indonesia | Rantepao (in Toraja Utara, Sulawesi Selatan) | 0.167 (0.117, 0.228) | | 4 (3, 5) | 7 (5, 9) | | | 6 (4, 8) | | 0.225 (0.118, 0.407) | |
| Indonesia | Kendari (in Kendari, Sulawesi Tenggara) | 0.133 (0.093, 0.182) | | 5 (4, 8) | 9 (7, 13) | | | 7 (5, 11) | | 0.198 (0.107, 0.354) | |
| Kenya | national | 0.011 (0.003, 0.033) | | 64 (26, 179) | NA | | | 92 (37, 259) | | 0.353 (0.151, 0.691) | |
| Laos | Champasak | 0.119 (0.062, 0.205) | | 6 (4, 12) | 11 (7, 20) | | | 9 (5, 17) | | 0.358 (0.158, 0.701 | |
| Laos | Savannakhet | 0.081 (0.044, 0.133) | | 8 (5, 13) | 14 (9, 23) | | | 12 (8, 19) | | 0.350 (0.152, 0.697) | |
| Malaysia | Federal territory of Kuala Lumpur, Perak, Kedah, Penang, Johor, Pahang, Kelantan and Sabah | 0.027 (0.009, 0.078) | | 27 (8, 74) | 48 (14, 129) | | | 40 (12, 107) | | 0.321 (0.141, 0.644) | |
| Malaysia | Damansara Damai | 0.033 (0.01, 0.075) | | 21 (10, 71) | 36 (17, 124) | | | 30 (14, 103) | | 0.371 (0.162, 0.737) | |
| Malaysia | Petaling district | 0.043 (0.025, 0.066) | | 17 (10, 26) | 29 (18, 45) | | | 24 (15, 37) | | 0.246 (0.129, 0.452) | |
| Malaysia | Sungai Segamat | 0.027 (0.014, 0.046) | | 26 (16, 48) | 45 (29, 83) | | | 37 (24, 69) | | 0.257 (0.123, 0.516) | |
| Malaysia | Peninsular Malaysia (urban) | 0.03 (0.014, 0.049) | | 23 (15, 51) | 41 (26, 89) | | | 34 (21, 74) | | 0.273 (0.127, 0.57) | |
| Malaysia | Peninsular Malaysia (rural) | 0.026 (0.013, 0.045) | | 26 (15, 49) | 46 (26, 86) | | | 38 (22, 71) | | 0.276 (0.13, 0.572) | |
| Malaysia | Damansara Damai (in the Petaling Jaya district) | 0.033 (0.01, 0.075) | | 21 (10, 61) | 37 (18, 107) | | | 30 (15, 89) | | 0.368 (0.165, 0.739) | |
| Malaysia | forest areas of Peninsular Malaysia | 0.023 (0.005, 0.074) | | 31 (10, 129) | 54 (18, 224) | | | 45 (15, 186) | | 0.389 (0.166, 0.763) | |
| Mexico | Yucatan | 0.045 (0.02, 0.081) | | 16 (9, 29) | 28 (16, 51) | | | 23 (14, 42) | | 0.296 (0.15, 0.557) | |
| Mexico | state of Morelos | 0.044 (0.018, 0.087) | | 16 (8, 36) | 28 (14, 63) | | | 23 (12, 53) | | 0.293 (0.133, 0.62) | |
| Nigeria | Osogbo, Osun State | 0.035 (0.009, 0.102) | | 21 (8, 66) | 37 (14, 114) | | | 31 (11, 95) | | 0.391 (0.166, 0.769) | |
| Pakistan | Lahore | 0.088 (0.033, 0.193) | | 8 (4, 24) | 14 (7, 41) | | | 12 (6, 34) | | 0.316 (0.142, 0.637) | |
| Saudi Arabia | Jeddah | 0.02 (0.011, 0.037) | | 36 (20, 64) | 62 (35, NA) | | | 51 (29, 92) | | 0.221 (0.11, 0.442) | |
| Saudi Arabia | Makkah | 0.021 (0.007, 0.058) | | 31 (12, 113) | 55 (21, 197) | | | 45 (17, 163) | | 0.34 (0.153, 0.658) | |
| Saudi Arabia | Madinah | 0.016 (0.004, 0.05) | | 43 (16, 149) | 75 (27, 259) | | | 62 (23, 215) | | 0.367 (0.157, 0.706) | |
| Saudi Arabia | Jeddah | 0.016 (0.004, 0.053) | | 37 (13, 162) | 65 (22, 282) | | | 54 (19, 234) | | 0.387 (0.167, 0.756) | |
| Saudi Arabia | Jizan | 0.023 (0.007, 0.06) | | 33 (15, 105) | 57 (26, 182) | | | 47 (21, 151) | | 0.341 (0.159, 0.656) | |
| Singapore | blood service | 0.049 (0.012, 0.148) | | 15 (5, 54) | 26 (8, 94) | | | 21 (7, 78) | | 0.405 (0.175, 0.736) | |
| Singapore | national | 0.02 (0.013, 0.03) | | 34 (24, 48) | 59 (42, 83) | | | 49 (35, 69) | | 0.202 (0.111, 0.371) | |
| Sri lanka | city of Colombo | 0.149 (0.076, 0.248) | | 4 (3, 9) | 8 (5, 15) | | | 6 (4, 13) | | 0.238 (0.118, 0.485) | |
| Sri lanka | Ramalana community in Colombo district | 0.049 (0.026, 0.08) | | 14 (8, 29) | 25 (14, 50) | | | 21 (12, 42) | | 0.308 (0.137, 0.63) | |
| Sri lanka | Trincomalee district | 0.063 (0.026, 0.133) | | 11 (5, 27) | 19 (9, 47) | | | 16 (8, 39) | | 0.314 (0.145, 0.636) | |
| Sri lanka | Jaffna district | 0.046 (0.017, 0.107) | | 15 (6, 41) | 26 (11, 72) | | | 22 (9, 60) | | 0.315 (0.145, 0.652) | |
| Sri lanka | Kurunegala district | 0.03 (0.009, 0.093) | | 23 (7, 77) | 40 (13, 134) | | | 33 (11, 111) | | 0.357 (0.152, 0.706) | |
| Sri lanka | Matara district | 0.032 (0.009, 0.093) | | 22 (7, 73) | 38 (13, 128) | | | 31 (11, 106) | | 0.356 (0.153, 0.701) | |
| Sri lanka | Ratnapura district | 0.04 (0.014, 0.102) | | 17 (7, 50) | 30 (12, 87) | | | 25 (10, 72) | | 0.344 (0.153, 0.676) | |
| Sri lanka | Polonaruwa district | 0.04 (0.014, 0.104) | | 17 (7, 50) | 30 (12, 87) | | | 25 (10, 72) | | 0.334 (0.148, 0.668) | |
| Sri lanka | Gampha district | 0.041 (0.014, 0.104) | | 17 (7, 48) | 29 (12, 84) | | | 24 (10, 70) | | 0.325 (0.146, 0.663) | |
| Sri lanka | Kandy district | 0.031 (0.01, 0.093) | | 22 (7, 72) | 39 (13, 125) | | | 32 (11, 104) | | 0.360 (0.151, 0.710) | |
| Sri lanka | Kandy district | 0.029 (0.008, 0.09) | | 24 (8, 84) | 41 (13, 146) | | | 34 (11, 121) | | 0.36 (0.152, 0.708) | |
| Sri lanka | Sri Jayewadenpura | 0.059 (0.04, 0.08) | | 12 (8, 17) | 20 (15, 30) | | | 17 (12, 25) | | 0.172 (0.091, 0.339) | |
| Taiwan | Taipei, Taoyuan, Tainan | 0.004 (0.002, 0.011) | | NA | NA | | | NA | | 0.258 (0.12, 0.53) | |
| Taiwan | Nanzih district, Kaohsiung City | 0.009 (0.004, 0.019) | | 71 (39, 166) | NA | | | NA | | 0.283 (0.139, 0.536) | |
| Taiwan | Sanmin district, Kaohsiung City | 0.005 (0.002, 0.012) | | NA | NA | | | NA | | 0.259 (0.123, 0.521) | |
| Tanzania | Buhigwe, Kalambo, Kilindi, Kinondoni, Kondoa, Kyela, Mvomero and Ukerewen | 0.011 (0.003, 0.035) | | NA | NA | | | 87 (36, 390 ) | | 0.352 (0.154, 0.698) | |
| Thailand | Ratchaburi province | 0.083 (0.035, 0.156) | | 8 (4, 23) | 14 (8, 40) | | | 12 (6, 33) | | 0.313 (0.144, 0.642) | |
| Thailand | Ratchaburi province | 0.089 (0.05, 0.134) | | 8 (5, 13) | 14 (9, 22) | | | 12 (8, 18) | | 0.239 (0.115, 0.496) | |
| Thailand | South (Narathiwat and Trang) | 0.079 (0.033, 0.168) | | 8 (4, 24) | 14 (8, 42) | | | 12 (6, 35) | | 0.347 (0.153, 0.72) | |
| Thailand | Central (Ayutthaya and Lop Buri) | 0.045 (0.019, 0.085) | | 15 (9, 28) | 26 (16, 48) | | | 22 (13, 40) | | 0.304 (0.135, 0.635) | |
| Thailand | Mukdahn | 0.095 (0.053, 0.163) | | 7 (4, 11) | 13 (7, 19) | | | 10 (6, 16) | | 0.376 (0.175, 0.705) | |
| Thailand | Ubon | 0.131 (0.067, 0.237) | | 5 (3, 11) | 9 (6, 20) | | | 8 (5, 16) | | 0.376 (0.161, 0.746) | |
| Vietnam | Nha Trang City, Nih Hoa district and Dien Khanh district (in Khanh Hoa province) | 0.012 (0.005, 0.029) | | 53 (27, 101) | 92 (46, 176) | | | 77 (38, 146) | | 0.268 (0.13, 0.526) | |

**Table S7. Region-specific FOI estimates obtained under model B1.** Specifically, the table reports the FOI estimated under model B1 (binomial likelihood) together with the age at which we expect 50% seroprevalence and 70% seroprevalence and the average age at first infection. The table also reports the estimates of the age decay alpha parameter obtained under model B1. “NA” is used when the average age was above 100 years old. Each measure is reported as median and 95% CrI.

| Country | Location | FOI | age_50%serop | age_70%serop | age_first_infection | alpha |
| --- | --- | --- | --- | --- | --- | --- |
| Bangladesh | blood service | \| 0.111 ( 0.053 , 0.19 ) \| \| --- \| | NA | NA | 9 ( 5 , 19 ) | 0.2 ( 0.09 , 0.34 ) |
| Haiti | Gressier, Jacmel, Chabin | \| 0.292 ( 0.213 , 0.414 ) \| \| --- \| | 3 ( 2, 4 ) | 8 ( 6 , 12 ) | 4 ( 3 , 5 ) | 0.11 ( 0.07 , 0.16 ) |
| India | Chennai | \| 0.067 ( 0.042 , 0.101 ) \| \| --- \| | NA | NA | 14 ( 11 , 24 ) | 0.28 ( 0.18 , 0.42 ) |
| Singapore | national | \| 0.005 ( 0.002 , 0.01 ) \| \| --- \| | 0 (0, 0) | NA | NA | 0.19 ( 0.08 , 0.35 ) |
| Tanzania | Zanzibar | \| 0.109 ( 0.066 , 0.24 ) \| \| --- \| | NA | NA | 9 ( 14 , 16 ) | 0.1 ( 0.05 , 0.23 ) |
| Venezuela | Cana de Azucar | \| 0.02 ( 0.012 , 0.034 ) \| \| --- \| | NA | NA | 52 ( 31 , 74 ) | 0.16 ( 0.08 , 0.3 ) |

**Table S8. Region-specific FOI estimates obtained under model B2.** Specifically, the table reports the FOI estimated under model B2 (beta-binomial likelihood) together with the age at which we expect 50% seroprevalence and 70% seroprevalence and the average age at first infection. The table also reports the estimates of the age decay alpha parameter and the over-dispersion parameter phi obtained under model B2. “NA” is used when the average age was above 100 years old. Each measure is reported as median and 95% CrI.

| Country | Location | FOI | age_50%  serop | age_70%  serop | age_first_  infection | | | alpha | phi |
| --- | --- | --- | --- | --- | --- | --- | --- | --- | --- |
| Bangladesh | blood service | \| 0.133 ( 0.038 , 0.288) \| \| --- \| | 10 ( 3 , 30 ) | 17 ( 15 , 19 ) | | 9 ( 3 , 28 ) | 0.16 ( 0.05 , 0.31 ) | | 0.26 ( 0.12 , 0.52 ) |
| Haiti | Gressier, Jacmel, Chabin | \| 0.209 ( 0.100 , 0.351) \| \| --- \| | 4 ( 3 , 8 ) | 10 ( 5 , 28 ) | | 5 ( 3 , 9 ) | 0.11 ( 0.04 , 0.23 ) | | 0.22 ( 0.11 , 0.42 ) |
| India | Chennai | \| 0.072 ( 0.024 , 0.172 ) \| \| --- \| | 21 (7 , 34 ) | NA | | 14 ( 7 , 41) | 0.19 ( 0.06 , 0.35 ) | | 0.21 ( 0.11 , 0.42 ) |
| Singapore | national | \| 0.034 ( 0.007 , 0.143 ) \| \| --- \| | NA | NA | | 27 ( 9 , 177) | 0.18 ( 0.04 , 0.35 ) | | 0.34 ( 0.15 , 0.66 ) |
| Tanzania | Zanzibar | \| 0.114 ( 0.034 , 0.254 ) \| \| --- \| | 7 ( 4 , 28 ) | 12 ( 8 , 18 ) | | 7 (4 , 24 ) | 0.13 ( 0.03 , 0.29 ) | | 0.32 ( 0.15 , 0.65 ) |
| Venezuela | Cana de Azucar | \| 0.059 ( 0.015 , 0.181 ) \| \| --- \| | 29 ( 6 , 40 ) | 99 ( 82 , 116) | | 15 ( 6 , 64 | 0.17 ( 0.03 , 0.34 ) | | 0.29 ( 0.13 , 0.59 ) |

**Table S9. Region-specific FOI estimates obtained under model C.** Specifically, the table reports the FOI estimated under model C (normal likelihood) together with the age at which we expect 50% seroprevalence and 70% seroprevalence and the average age at first infection. “NA” is used when the average age was above 100 years old. Each measure is reported as median and 95% CrI.

| Country | Location | FOI | Age_50%serop | Age_70%serop | Age_first_infection |
| --- | --- | --- | --- | --- | --- |
| Brazil | Fortaleza | 0.096 (0.08 , 0.114 ) | 7 (6 , 9 ) | 13 (11 , 15 ) | 11 (9 , 13 ) |
| French Guiana | national | 0.069 (0.042, 0.173) | 10 (4, 16) | 17 (7, 27) | 14 (6, 22) |
| French Polynesia | Tahiti | 0.091 (0.077, 0.108) | 8 (6, 9) | 13 (11, 15) | 11 (9, 13) |
| Kenya | Kilifi (Junju and Ngernya dublocations) | 0.045 (0.038, 0.052) | 16 (13, 19) | 27 (23, 33) | 22 (19, 27) |
| Laos | Xay, Xiengngeun, Viengxay, Pek districts | 0.001 (0, 0.005) | NA | NA | NA |
| Peru | Iquitos | 0.051 (0.044, 0.066) | 13(11, 15) | 23 (20, 27) | 19 (16. 22) |

**Table S10. City-specific FOI estimates obtained under model A1 in Mexico [63].** Specifically, the table reports the FOI estimated under model A1 (binomial likelihood) together with the age at which we expect 50% seroprevalence and 70% seroprevalence and the average age at first infection. The table also reports the estimates of the age decay alpha parameter obtained under model B1. “NA” is used when the average age was above 100 years old. Each measure is reported as median and 95% CrI.

| City in Mexico | FOI | Age_50%serop | Age_70%serop | Age_first_infection |
| --- | --- | --- | --- | --- |
| Acapulco | 0.225 (0.164, 0.315) | NA | NA | NA |
| Ahome | 0.054 (0.026, 0.1) | NA | NA | NA |
| Apaseo el Agrande | 0.003 (0, 0.017) | 214 (40, 5799) | 372 (70, 10073) | 309 (58, 8366) |
| Cancun | 0.119 (0.072, 0.183) | 6 (4, 10) | 10 (7, 17) | 8 (5, 14) |
| Cardenas | 0.131 (0.092, 0.181) | 5 (4, 8) | 9 (7, 13) | 8 (6, 11) |
| Chetumal | 0.079 (0.046, 0.128) | 9 (5, 15) | 15 (9, 26) | 13 (8, 22) |
| Chilpancingo | 0.047 (0.025, 0.078) | 15 (9, 27) | 25 (15, 48) | 21 (13, 39) |
| Ciudad Acuña | 0.034 (0.011, 0.074) | 20 (9, 61) | 35 (16, 105) | 29 (13, 87) |
| Ciudad Apodaca | 0.011 (0.002, 0.031) | 61 (23, 278) | 107 (39, 483) | 89 (33, 401) |
| Ciudad Benito Juarez | 0.013 (0.002, 0.044) | NA | NA | NA |
| Ciudad del Carmen | 0.093 (0.053, 0.15) | 7 (5, 13) | 13 (8, 23) | 11 (7, 19) |
| Ciudad Victoria | 0.029 (0.015, 0.05) | 24 (14, 46) | 42 (24, 100) | 35 (20, 66) |
| Coatzacoalcos | 0.069 (0.027, 0.143) | NA | NA | NA |
| Comalcalco | 0.104 (0.064, 0.157) | NA | NA | NA |
| Cordoba | 0.151 (0.074, 0.291) | NA | NA | NA |
| Cuernavaca | 0.081 (0.043, 0.14) | NA | NA | NA |
| Culiacan | 0.128 (0.058, 0.243) | 5 (3, 12) | 9 (5, 21) | 8 (4, 17) |
| Ebano | 0.025 (0.011, 0.049) | 28 (14, 66) | 48 (25, 114) | 40 (20, 95) |
| El Salto | 0.007 (0.001, 0.023) | 98 (30, 620) | 171 (52, 1077) | 142 (43, 894) |
| Empalme Escobedo | 0.003 (0, 0.016) | 237 (43, 6200) | 412 (75, 10769) | 342 (63, 8944) |
| Etzatlan | 0.002 (0, 0.01) | NA | NA | NA |
| General Escobedo | 0.007 (0.001, 0.022) | NA | NA | NA |
| Guadalajara | 0.031 (0.013, 0.059) | 23 (12, 53) | 39 (20, 92) | 32 (17, 76) |
| Guaymas | 0.077 (0.04, 0.132) | NA | NA | NA |
| Hermosillo | 0.016 (0.007, 0.029) | 44 (24, 93) | 77 (42, 162) | 64 (35, 134) |
| Macuspana | 0.121 (0.065, 0.205) | NA | NA | NA |
| Mapastepec | 0.451 (0.304, 0.706) | NA | NA | NA |
| Matamoros | 0.034 (0.019, 0.056) | 20 (12, 37) | 35 (22, 64) | 29 (18, 53) |
| Mazatlan | 0.071 (0.032, 0.139) | 10 (5, 22) | 17 (9, 38) | 14 (7, 31) |
| Merida | 0.071 (0.054, 0.093) | 10 (7, 13) | 17 (13, 22) | 14 (11, 19) |
| Mexicali | 0.007 (0.001, 0.023) | 102 (31, 743) | 177 (53, 1290) | 147 (44, 1072) |
| Monterrey | 0.015 (0.008, 0.026) | 46 (27, 100) | NA | 66 (39, 100) |
| Navolato | 0.129 (0.08, 0.198) | 5 (3, 100) | 9 (6, 100) | 8 (5, 100) |
| Nuevo laredo | 0.026 (0.009, 0.055) | NA | NA | NA |
| Oaxaca | 0.03 (0.015, 0.056) | NA | NA | NA |
| Orizaba | 0.011 (0.003, 0.029) | 63 (24, 100) | 110 (41, 100) | 91 (34, 100) |
| Piedras negras | 0.002 (0, 0.008) | 438 (82, 14327) | 760 (142, 24886) | 631 (118, 20670) |
| Poza Rica | 0.131 (0.077, 0.215) | 5 (3, 9) | 9 (6, 16) | 8 (5, 13) |
| Progreso | 0.108 (0.069, 0.161) | 6 (4, 10) | 11 (7, 17) | 9 (6, 15) |
| Reynosa | 0.042 (0.021, 0.074) | 17 (9, 33) | 29 (16, 58) | 24 (14, 48) |
| Salamanca | 0.001 (0, 0.007) | 505 (97, 14134) | 877 (168, 24550) | 728 (139, 20391) |
| San Francisco de Campeche | 0.171 (0.104, 0.276) | 4 (3, 7) | 7 (4, 12) | 6 (4, 10) |
| San Gabriel Chilac | 0.031 (0.015, 0.055) | NA | NA | NA |
| San Jose del Cabo | 0.052 (0.027, 0.091) | 13 (8, 26) | 23 (13, 45) | 19 (11, 38) |
| San Pedro | 0.058 (0.025, 0.116) | 12 (6, 28) | 21 (10, 48) | 17 (9, 40) |
| San Pedro Pochutla | 0.152 (0.085, 0.26) | 5 (3, 8) | 8 (5, 14) | 7 (4, 12) |
| Santa Catarina | 0.013 (0.003, 0.035) | 52 (20, 217) | 90 (34, 378) | 74 (28, 314) |
| Tanquián de Escobedo | 0.003 (0, 0.018) | 204 (38, 5696) | 354 (65, 9894) | 294 (54, 8217) |
| Tapachula | 0.274 (0.202, 0.37) | 3 (2, 3) | 4 (3, 100) | 4 (3, 100) |
| Tecpan de Galeana | 0.235 (0.16, 0.342) | 3 (2, 4) | 5 (4, 8) | 4 (3, 6) |
| Teocaltiche | 0.003 (0, 0.015) | 236 (45, 5782) | 410 (78, 10043) | 340 (65, 8341) |
| Tepic | 0.055 (0.03, 0.089) | 13 (8, 23) | 22 (14, 40) | 18 (11, 33) |
| Tijuana | 0.004 (0.001, 0.015) | 160 (47, 1089) | 277 (82, 1891) | 230 (69, 1571) |
| Tlapacoyan | 0.124 (0.068, 0.21) | 6 (3, 10) | 10 (6, 18) | 8 (5, 15) |
| Tonala | 0.024 (0.011, 0.045) | 29 (15, 66) | 50 (26, 100) | 42 (22, 95) |
| Tuxtla Gutierrez | 0.159 (0.102, 0.237) | 4 (3, 7) | 8 (5, 12) | 6 (4, 10) |
| Uruapan | 0.004 (0.001, 0.014) | 166 (49, 1161) | 288 (85, 2016) | 240 (70, 1674) |
| Valle de Santiago | 0.002 (0, 0.011) | 335 (63, 9154) | 582 (110, 15901) | 484 (91, 13207) |
| Veracruz | 0.13 (0.085, 0.194) | 5 (4, 100) | NA | NA |
| Xalapa | 0.005 (0.002, 0.01) | 149 (69, 427) | 260 (120, 741) | 216 (99, 616) |
| Zacatepec | 0.046 (0.018, 0.095) | 15 (7, 38) | 26 (13, 67) | 22 (11, 55) |
| Zamora | 0.032 (0.012, 0.066) | 22 (10, 56) | 38 (18, 97) | 31 (15, 80) |
| Zapopan | 0.005 (0.001, 0.016) | NA | NA | NA |
| Zaragoza | 0.019 (0.007, 0.042) | 36 (17, 106) | 63 (29, 184) | 52 (24, 153) |
| Zihuatanejo | 0.649 (0.245, 0.984) | 1 (1, 3) | 2 (1, 5) | 2 (1, 4) |

**Table S11. City-specific FOI estimates obtained under model A1 in Madagascar [56].** Specifically, the table reports the FOI estimated under model A1 (binomial likelihood) together with the age at which we expect 50% seroprevalence and 70% seroprevalence and the average age at first infection. The table also reports the estimates of the age decay alpha parameter obtained under model B1. “NA” is used when the average age was above 100 years old. Each measure is reported as median and 95% CrI.

| City | FOI | Age_50%serop | Age_70%serop | Age_first_infection |
| --- | --- | --- | --- | --- |
| Ambovombe | 0.001 (0, 0.003) | NA | NA | NA |
| Ambatondrazaka | 0.001 (0, 0.002) | NA | NA | NA |
| Antsohihy | 0.002 (0.001, 0.004) | NA | NA | NA |
| Anjozorobe | 0 (0, 0.002) | NA | NA | NA |
| Antsirabe | 0 (0, 0.001) | NA | NA | NA |
| Belo | 0.002 (0.001, 0.004) | NA | NA | NA |
| Ambato-Boeny | 0.002 (0.001, 0.005) | NA | NA | NA |
| Ambositra | 0 (0, 0.001) | NA | NA | NA |
| Antananarivo-Renivohitra | 0.001 (0, 0.002) | NA | NA | NA |
| Antsiranana | 0.004 (0.002, 0.007) | NA | NA | NA |
| Ampanihy | 0 (0, 0.002) | NA | NA | NA |
| Farafangana | 0.002 (0.001, 0.005) | NA | NA | NA |
| Fianarantsoa | 0 (0, 0.001) | NA | NA | NA |
| Ihosy | 0.002 (0.001, 0.004) | NA | NA | NA |
| Maevatanana | 0 (0, 0.002) | NA | NA | NA |
| Mandritsara | 0 (0, 0.001) | NA | NA | NA |
| Morondava | 0.002 (0.001, 0.004) | NA | NA | NA |
| Miandrivazo | 0.001 (0, 0.002) | NA | NA | NA |
| Mahajanga | 0.001 (0, 0.002) | NA | NA | NA |
| Mananjary | 0.004 (0.002, 0.007) | NA | NA | NA |
| Morombe | 0.001 (0, 0.003) | NA | NA | NA |
| Moramanga | 0.001 (0, 0.003) | NA | NA | NA |
| Nosy-Be | 0.017 (0.011, 0.024) | 41 (29, 61) | 71 (50, 105) | 59 (42, 88) |
| Sambava | 0.001 (0, 0.002) | NA | NA | NA |
| Tsiroanomandidy | 0.001 (0, 0.003) | NA | NA | NA |
| Taolagnaro | 0.002 (0.001, 0.005) | NA | NA | NA |
| Toamasina | 0.016 (0.01, 0.023) | 44 (30, 66) | 76 (53, 115) | 63 (44, 96) |
| Toliary | 0.001 (0, 0.003) | NA | NA | NA |

### **Table S12.** **Summary of FOI estimates identified in this review and in Cattarino et al [15].** NA was used when the location was not specified. The table reports the country, location, date of the survey, median FOI and the source of the estimate (Cattarino et al 2020 or the current Review).

| **Country** | **Location** | **Study date** | **FOI** | **Source** |
| --- | --- | --- | --- | --- |
| **Australia** | Charters Towers | 1993 | 0.003 | Cattarino et al 2020 |
| **Bangladesh** | blood service | 2022 | 0.133 | Review |
| **Bangladesh** | Dhaka city | 2012 | 0.045 | Review |
| **Bangladesh** | Dhaka City Corporation | 2012 | 0.014 | Cattarino et al 2020 |
| **Bangladesh** | NA | 2014-2016 | 0.002 | Cattarino et al 2020 |
| **Bangladesh** | NA | 2014-2016 | 0.002 | Cattarino et al 2020 |
| **Bangladesh** | NA | 2014-2016 | 0.001 | Cattarino et al 2020 |
| **Bangladesh** | NA | 2014-2016 | 0.003 | Cattarino et al 2020 |
| **Bangladesh** | NA | 2014-2016 | 0.004 | Cattarino et al 2020 |
| **Bangladesh** | NA | 2014-2016 | 0.042 | Cattarino et al 2020 |
| **Bangladesh** | NA | 2014-2016 | 0.001 | Cattarino et al 2020 |
| **Bangladesh** | NA | 2014-2016 | 0.031 | Cattarino et al 2020 |
| **Bangladesh** | NA | 2014-2016 | 0.002 | Cattarino et al 2020 |
| **Bangladesh** | NA | 2014-2016 | 0.003 | Cattarino et al 2020 |
| **Bangladesh** | NA | 2014-2016 | 0.002 | Cattarino et al 2020 |
| **Bangladesh** | NA | 2014-2016 | 0.001 | Cattarino et al 2020 |
| **Bangladesh** | NA | 2014-2016 | 0.003 | Cattarino et al 2020 |
| **Bangladesh** | NA | 2014-2016 | 0.001 | Cattarino et al 2020 |
| **Bangladesh** | NA | 2014-2016 | 0.001 | Cattarino et al 2020 |
| **Bangladesh** | NA | 2014-2016 | 0.001 | Cattarino et al 2020 |
| **Bangladesh** | NA | 2014-2016 | 0.001 | Cattarino et al 2020 |
| **Bangladesh** | NA | 2014-2016 | 0.002 | Cattarino et al 2020 |
| **Bangladesh** | NA | 2014-2016 | 0.002 | Cattarino et al 2020 |
| **Bangladesh** | NA | 2014-2016 | 0.002 | Cattarino et al 2020 |
| **Bangladesh** | NA | 2014-2016 | 0.002 | Cattarino et al 2020 |
| **Bangladesh** | NA | 2014-2016 | 0.003 | Cattarino et al 2020 |
| **Bangladesh** | NA | 2014-2016 | 0.033 | Cattarino et al 2020 |
| **Bangladesh** | NA | 2014-2016 | 0.005 | Cattarino et al 2020 |
| **Bangladesh** | NA | 2014-2016 | 0.006 | Cattarino et al 2020 |
| **Bangladesh** | NA | 2014-2016 | 0.004 | Cattarino et al 2020 |
| **Bangladesh** | NA | 2014-2016 | 0.009 | Cattarino et al 2020 |
| **Bangladesh** | NA | 2014-2016 | 0.001 | Cattarino et al 2020 |
| **Bangladesh** | NA | 2014-2016 | 0.000 | Cattarino et al 2020 |
| **Bangladesh** | NA | 2014-2016 | 0.001 | Cattarino et al 2020 |
| **Bangladesh** | NA | 2014-2016 | 0.001 | Cattarino et al 2020 |
| **Bangladesh** | NA | 2014-2016 | 0.001 | Cattarino et al 2020 |
| **Bangladesh** | NA | 2014-2016 | 0.001 | Cattarino et al 2020 |
| **Bangladesh** | NA | 2014-2016 | 0.004 | Cattarino et al 2020 |
| **Bangladesh** | NA | 2014-2016 | 0.002 | Cattarino et al 2020 |
| **Bangladesh** | NA | 2014-2016 | 0.001 | Cattarino et al 2020 |
| **Bangladesh** | NA | 2014-2016 | 0.005 | Cattarino et al 2020 |
| **Bangladesh** | NA | 2014-2016 | 0.003 | Cattarino et al 2020 |
| **Bangladesh** | NA | 2014-2016 | 0.002 | Cattarino et al 2020 |
| **Bangladesh** | NA | 2014-2016 | 0.004 | Cattarino et al 2020 |
| **Bangladesh** | NA | 2014-2016 | 0.009 | Cattarino et al 2020 |
| **Bangladesh** | NA | 2014-2016 | 0.010 | Cattarino et al 2020 |
| **Bangladesh** | NA | 2014-2016 | 0.004 | Cattarino et al 2020 |
| **Bangladesh** | NA | 2014-2016 | 0.001 | Cattarino et al 2020 |
| **Bangladesh** | NA | 2014-2016 | 0.005 | Cattarino et al 2020 |
| **Bangladesh** | NA | 2014-2016 | 0.018 | Cattarino et al 2020 |
| **Bangladesh** | NA | 2014-2016 | 0.019 | Cattarino et al 2020 |
| **Bangladesh** | NA | 2014-2016 | 0.001 | Cattarino et al 2020 |
| **Bangladesh** | NA | 2014-2016 | 0.007 | Cattarino et al 2020 |
| **Bangladesh** | NA | 2014-2016 | 0.001 | Cattarino et al 2020 |
| **Bangladesh** | NA | 2014-2016 | 0.002 | Cattarino et al 2020 |
| **Bangladesh** | NA | 2014-2016 | 0.003 | Cattarino et al 2020 |
| **Bangladesh** | NA | 2014-2016 | 0.002 | Cattarino et al 2020 |
| **Bangladesh** | NA | 2014-2016 | 0.005 | Cattarino et al 2020 |
| **Bangladesh** | NA | 2014-2016 | 0.001 | Cattarino et al 2020 |
| **Bangladesh** | NA | 2014-2016 | 0.002 | Cattarino et al 2020 |
| **Bangladesh** | NA | 2014-2016 | 0.001 | Cattarino et al 2020 |
| **Bangladesh** | NA | 2014-2016 | 0.001 | Cattarino et al 2020 |
| **Bangladesh** | NA | 2014-2016 | 0.001 | Cattarino et al 2020 |
| **Bangladesh** | NA | 2014-2016 | 0.001 | Cattarino et al 2020 |
| **Bangladesh** | NA | 2014-2016 | 0.001 | Cattarino et al 2020 |
| **Bangladesh** | NA | 2014-2016 | 0.001 | Cattarino et al 2020 |
| **Bangladesh** | NA | 2014-2016 | 0.001 | Cattarino et al 2020 |
| **Bangladesh** | NA | 2014-2016 | 0.001 | Cattarino et al 2020 |
| **Bangladesh** | NA | 2014-2016 | 0.001 | Cattarino et al 2020 |
| **Bangladesh** | NA | 2014-2016 | 0.002 | Cattarino et al 2020 |
| **Bangladesh** | NA | 2014-2016 | 0.001 | Cattarino et al 2020 |
| **Bangladesh** | NA | 2014-2016 | 0.001 | Cattarino et al 2020 |
| **Bangladesh** | NA | 2014-2016 | 0.000 | Cattarino et al 2020 |
| **Bangladesh** | NA | 2014-2016 | 0.000 | Cattarino et al 2020 |
| **Brazil** | Sau Paulo | 2015-2016 | 0.030 | Review |
| **Brazil** | Fortaleza | 2011-2015 | 0.096 | Review |
| **Brazil** | Fortaleza | 2019-2020 | 0.086 | Review |
| **Brazil** | Recife (city) | 2018-2019 | 0.052 | Review |
| **Brazil** | Tapera (village), in Itarema (municipality) in state of Ceara | 2015 | 0.009 | Review |
| **Brazil** | Recife, State of Pernambuco | 2005_2006 | 0.014 | Cattarino et al 2020 |
| **Brazil** | Niterói, State of Rio de Janeiro | 1991 | 0.026 | Cattarino et al 2020 |
| **Brazil** | Paracambi, State of Rio de Janeiro | 1994 | 0.013 | Cattarino et al 2020 |
| **Brazil** | Acrelândia, State of Acre | 2004_2005 | 0.002 | Cattarino et al 2020 |
| **Brazil** | São Luis Island, State of Maranhão | 1996 | 0.003 | Cattarino et al 2020 |
| **Brazil** | Campinas, State of São Paulo | 1998 | 0.002 | Cattarino et al 2020 |
| **Burkina Faso** | Ougadougou | 2015-2017 | 0.063 | Review |
| **Cameroon** | Doualaa (town) | 2006-2007 | 0.034 | Review |
| **Cameroon** | Garoua (town) | 2006-2007 | 0.009 | Review |
| **Cameroon** | Garoua | 2006 | 0.002 | Cattarino et al 2020 |
| **Cameroon** | Douala | 2006 | 0.009 | Cattarino et al 2020 |
| **Cameroon** | Yaounde | 2006 | 0.001 | Cattarino et al 2020 |
| **Colombia** | Anapoima, Apulo, Buenaventura, Quibdo, Tumaco,Tierralta | 2013-2015 | 0.047 | Review |
| **Colombia** | Quobdo | 2015* | 0.041 | Review |
| **Colombia** | Santander | 2015* | 0.029 | Cattarino et al 2020 |
| **Costa Rica** | San José | 2002_2003 | 0.028 | Cattarino et al 2020 |
| **Cuba** | Santiago de Cuba | 1997_1998 | 0.004 | Cattarino et al 2020 |
| **Cuba** | El Cerro | 1983 | 0.010 | Cattarino et al 2020 |
| **Djibouti** | national | 2010-2011 | 0.008 | Review |
| **Dominican Republic** | Santo Domingo district, Santo Domingo | 2002 | 0.029 | Cattarino et al 2020 |
| **Ecuador** | Town of Quininde | 2005-2009 | 0.022 | Review |
| **El Salvador** | Aguilares | 2000_2001 | 0.031 | Cattarino et al 2020 |
| **France (Carribean)** | Guadeloupe and Martinique | 2011 | 0.078 | Review |
| **French Guiana** | national | 2017 | 0.069 | Review |
| **French Polynesia** | Tahiti | 2014-2015 | 0.091 | Review |
| **Haiti** | Gressier, Jacmel, Chabin | 2013 | 0.209 | Review |
| **India** | Delhi | 2011-2012 | 0.136 | Review |
| **India** | Kalayani | 2011-2013 | 0.035 | Review |
| **India** | Wardha | 2011-2014 | 0.144 | Review |
| **India** | Mumbai | 2011-2015 | 0.22 | Review |
| **India** | Hyderabad | 2011-2016 | 0.116 | Review |
| **India** | Bangalore | 2011-2017 | 0.141 | Review |
| **India** | North (Delhi, Punjab and Uttar Pradesh) | 2017-2018 | 0.058 | Review |
| **India** | NorthEast (Tripura, Meghalaya and Assam) | 2017-2018 | 0.002 | Review |
| **India** | East (Bihar, West Bengal and Odisha) | 2017-2018 | 0.008 | Review |
| **India** | West (Rajasthan, Madhya Pradesh and Maharashtra) | 2017-2018 | 0.048 | Review |
| **India** | South (Andhra Pradesh, Karnataka and Tamil Nadu) | 2017-2018 | 0.069 | Review |
| **India** | Chennai | 2011 | 0.088 | Review |
| **India** | Delhi | 2012 | 0.043 | Review |
| **India** | Pune | 2017 | 0.078 | Review |
| **India** | Vaddu area | 2011 | 0.021 | Review |
| **India** | Chennai, Tamil Nadu | 2017-2018 | 0.007 | Review |
| **India** | Kerala (urban Kanjiarappally) | 2016 | 0.024 | Review |
| **India** | Kerala ( rural as Koruthodu and Erumeli in Kottayam district) | 2016 | 0.016 | Review |
| **India** | Maharashtra (Vaddu area) 2014 | 2014 | 0.017 | Review |
| **India** | Maharashtra (Vaddu area) 2016 | 2016 | 0.024 | Review |
| **India** | Pune 2019 | 2019 | 0.059 | Review |
| **India** | Pune 2009 | 2009 | 0.037 | Review |
| **India** | Andaman Islands | 1988_1989 | 0.001 | Cattarino et al 2020 |
| **India** | Chennai | 2011 | 0.040 | Cattarino et al 2020 |
| **India** | New Delhi | 2011-2012 | 0.030 | Cattarino et al 2020 |
| **India** | New Delhi | 2011-2012 | 0.035 | Cattarino et al 2020 |
| **India** | Kalyani | 2011-2012 | 0.010 | Cattarino et al 2020 |
| **India** | Wardha | 2011-2012 | 0.038 | Cattarino et al 2020 |
| **India** | Mumbai | 2011-2012 | 0.051 | Cattarino et al 2020 |
| **India** | Medchal | 2011-2012 | 0.029 | Cattarino et al 2020 |
| **India** | Medchal | 2011-2012 | 0.029 | Cattarino et al 2020 |
| **India** | Bangalore | 2011-2012 | 0.032 | Cattarino et al 2020 |
| **India** | Pune, Koregaon Bhima | 2011 | 0.011 | Cattarino et al 2020 |
| **India** | Pune, Pimpale Jagatap | 2012 | 0.006 | Cattarino et al 2020 |
| **Indonesia** | Denpasar Bali | 2020-2021 | 0.036 | Review |
| **Indonesia** | national | 2014 | 0.144 | Review |
| **Indonesia** | national | 2014 | 0.149 | Review |
| **Indonesia** | Simpang Kiri (in Subulussalam, Nanggroe Aceh Darussalam) | 2014 | 0.126 | Review |
| **Indonesia** | Medan Denai (in Medan, Sumatera Utara) | 2014 | 0.188 | Review |
| **Indonesia** | Pauh (in Padang, Sumatera Barat) | 2014 | 0.164 | Review |
| **Indonesia** | Bungo Dani (in Bungo, Jambi) | 2014 | 0.225 | Review |
| **Indonesia** | Kalianda (in Lampung Selatan, Lampung) | 2014 | 0.073 | Review |
| **Indonesia** | Cikupa (in Tangerang, Banten) | 2014 | 0.262 | Review |
| **Indonesia** | Benda (in Tangerang, Banten) | 2014 | 0.122 | Review |
| **Indonesia** | Pesanggrahan (in Jakarta Selatan, Dki Jakarta) | 2014 | 0.196 | Review |
| **Indonesia** | Pulo Gadung (in Jakarta Timur, Dki Jakarta) | 2014 | 0.157 | Review |
| **Indonesia** | Kali Deres (in Jakarta Barat, Dki Jakarta) | 2014 | 0.143 | Review |
| **Indonesia** | Gunung Putri (in Bogor, Jawa Barat) | 2014 | 0.162 | Review |
| **Indonesia** | Banjaran (in Bandung, Jawa Barat) | 2014 | 0.124 | Review |
| **Indonesia** | Gunung Sari (in Cirebon, Jawa Barat) | 2014 | 0.22 | Review |
| **Indonesia** | Cikarang Utara (in Bekasi, Jawa Barat) | 2014 | 0.212 | Review |
| **Indonesia** | Bojongloa Kaler (in Bandung, Jawa Barat) | 2014 | 0.253 | Review |
| **Indonesia** | Bekasi Timur (in Bekasi, Jawa Barat) | 2014 | 0.154 | Review |
| **Indonesia** | Singaparna (in Tasikmalaya, Jawa Barat) | 2014 | 0.068 | Review |
| **Indonesia** | Trucuk (in Klaten, Jawa Tengah) | 2014 | 0.115 | Review |
| **Indonesia** | Pecangaan (in Jepara, Jawa Tengah) | 2014 | 0.225 | Review |
| **Indonesia** | Dukuhturi (in Tegal, Jawa Tengah) | 2014 | 0.288 | Review |
| **Indonesia** | Tegal Barat (in Tegal, Jawa Tengah) | 2014 | 0.296 | Review |
| **Indonesia** | Pulung (in Ponorogo, Jawa Timur) | 2014 | 0.047 | Review |
| **Indonesia** | Cluring (in Banyuwangi, Jawa Timur) | 2014 | 0.137 | Review |
| **Indonesia** | Ngoro (in Mojokerto, Jawa Timur) | 2014 | 0.233 | Review |
| **Indonesia** | Kalianget (in Sumenep, Jawa Timur) | 2014 | 0.132 | Review |
| **Indonesia** | Sawahan (in Surabaya, Jawa Timur) | 2014 | 0.258 | Review |
| **Indonesia** | Denpasar Selatan (in Denpasar, Bali) | 2014 | 0.147 | Review |
| **Indonesia** | Samarinda Ulu (in Samarinda, Kalimantan Timur) | 2014 | 0.118 | Review |
| **Indonesia** | Rantepao (in Toraja Utara, Sulawesi Selatan) | 2014 | 0.068 | Review |
| **Indonesia** | Kendari (in Kendari, Sulawesi Tenggara) | 2014 | 0.336 | Review |
| **Indonesia** | Gondokusuman subdistrict, Yogyakarta province | 1995 | 0.030 | Cattarino et al 2020 |
| **Indonesia** | Jakarta | 2014 | 0.037 | Cattarino et al 2020 |
| **Kenya** | national | 2007 | 0.004 | Review |
| **Kenya** | Kilifi (Junju and Ngernya dublocations) | 1998-2018 | 0.045 | Review |
| **Kenya** | Kenya | 2007 | 0.002 | Cattarino et al 2020 |
| **Kenya** | Kwale County | 2009-2011 | 0.006 | Cattarino et al 2020 |
| **Kenya** | Busia | 2004 | 0.000 | Cattarino et al 2020 |
| **Kenya** | Malindi | 2004 | 0.003 | Cattarino et al 2020 |
| **Kenya** | Maralal | 2004 | 0.000 | Cattarino et al 2020 |
| **Laos** | Champasak | 2019 | 0.216 | Review |
| **Laos** | Savannakhet | 2019 | 0.081 | Review |
| **Laos** | Xay, Xiengngeun, Viengxay, Pek districts | 2009 | 0.001 | Review |
| **Laos** | Khammouane | 2007_2008 | 0.005 | Cattarino et al 2020 |
| **Laos** | Vientiane | 2006 | 0.010 | Cattarino et al 2020 |
| **Madagascar** | Ambovombe | 2011-2013 | 0.001 | Review |
| **Madagascar** | Ambatondrazaka | 2011-2013 | 0.001 | Review |
| **Madagascar** | Antsohihy | 2011-2013 | 0.002 | Review |
| **Madagascar** | Anjozorobe | 2011-2013 | 0.000 | Review |
| **Madagascar** | Antsirabe | 2011-2013 | 0.000 | Review |
| **Madagascar** | Belo | 2011-2013 | 0.002 | Review |
| **Madagascar** | Ambato-Boeny | 2011-2013 | 0.002 | Review |
| **Madagascar** | Ambositra | 2011-2013 | 0.000 | Review |
| **Madagascar** | Antananarivo-Renivohitra | 2011-2013 | 0.001 | Review |
| **Madagascar** | Antsiranana | 2011-2013 | 0.004 | Review |
| **Madagascar** | Ampanihy | 2011-2013 | 0.000 | Review |
| **Madagascar** | Farafangana | 2011-2013 | 0.002 | Review |
| **Madagascar** | Fianarantsoa | 2011-2013 | 0.000 | Review |
| **Madagascar** | Ihosy | 2011-2013 | 0.002 | Review |
| **Madagascar** | Maevatanana | 2011-2013 | 0.000 | Review |
| **Madagascar** | Mandritsara | 2011-2013 | 0.000 | Review |
| **Madagascar** | Morondava | 2011-2013 | 0.002 | Review |
| **Madagascar** | Miandrivazo | 2011-2013 | 0.001 | Review |
| **Madagascar** | Mahajanga | 2011-2013 | 0.001 | Review |
| **Madagascar** | Mananjary | 2011-2013 | 0.004 | Review |
| **Madagascar** | Morombe | 2011-2013 | 0.001 | Review |
| **Madagascar** | Moramanga | 2011-2013 | 0.001 | Review |
| **Madagascar** | Nosy-Be | 2011-2013 | 0.017 | Review |
| **Madagascar** | Sambava | 2011-2013 | 0.001 | Review |
| **Madagascar** | Tsiroanomandidy | 2011-2013 | 0.001 | Review |
| **Madagascar** | Taolagnaro | 2011-2013 | 0.002 | Review |
| **Madagascar** | Toamasina | 2011-2013 | 0.016 | Review |
| **Madagascar** | Toliary | 2011-2013 | 0.001 | Review |
| **Malaysia** | Federal territory of Kuala Lumpur, Perak, Kedah, Penang, Johor, Pahang, Kelantan and Sabah | 2008-2009 | 0.010 | Review |
| **Malaysia** | Damansara Damai | 2018-2019 | 0.033 | Review |
| **Malaysia** | Peninsular Malaysia | 2006-2012 | 0.038 | Review |
| **Malaysia** | Petaling district | 2018 | 0.059 | Review |
| **Malaysia** | Sungai Segamat | 2015 | 0.029 | Review |
| **Malaysia** | forest areas of Peninsular Malaysia | 2007-2010 | 0.023 | Review |
| **Malaysia** | Segamat district, Johor state | 2015 | 0.008 | Cattarino et al 2020 |
| **Mayotte** | Mayotte | 2006 | 0.003 | Cattarino et al 2020 |
| **Mexico** | Acapulco | 2016 | 0.225 | Review |
| **Mexico** | Ahome | 2016 | 0.054 | Review |
| **Mexico** | Apaseo el Grande | 2016 | 0.003 | Review |
| **Mexico** | Cancun | 2016 | 0.119 | Review |
| **Mexico** | Cardenas | 2016 | 0.131 | Review |
| **Mexico** | Chetumal | 2016 | 0.079 | Review |
| **Mexico** | Chilpancingo | 2016 | 0.047 | Review |
| **Mexico** | Ciudad Acuña | 2016 | 0.034 | Review |
| **Mexico** | Ciudad Apodaca | 2016 | 0.011 | Review |
| **Mexico** | Ciudad Benito Juarez | 2016 | 0.013 | Review |
| **Mexico** | Ciudad del Carmen | 2016 | 0.093 | Review |
| **Mexico** | Ciudad Victoria | 2016 | 0.029 | Review |
| **Mexico** | Coatzacoalcos | 2016 | 0.069 | Review |
| **Mexico** | Comalcalco | 2016 | 0.104 | Review |
| **Mexico** | Cordoba | 2016 | 0.151 | Review |
| **Mexico** | Cuernavaca | 2016 | 0.081 | Review |
| **Mexico** | Culiacan | 2016 | 0.128 | Review |
| **Mexico** | Ebano | 2016 | 0.025 | Review |
| **Mexico** | El Salto | 2016 | 0.007 | Review |
| **Mexico** | Empalme Escobedo | 2016 | 0.003 | Review |
| **Mexico** | Etzatlan | 2016 | 0.002 | Review |
| **Mexico** | General Escobedo | 2016 | 0.007 | Review |
| **Mexico** | Guadalajara | 2016 | 0.031 | Review |
| **Mexico** | Guaymas | 2016 | 0.077 | Review |
| **Mexico** | Hermosillo | 2016 | 0.016 | Review |
| **Mexico** | Macuspana | 2016 | 0.121 | Review |
| **Mexico** | Mapastepec | 2016 | 0.451 | Review |
| **Mexico** | Matamoros | 2016 | 0.034 | Review |
| **Mexico** | Mazatlan | 2016 | 0.071 | Review |
| **Mexico** | Merida | 2016 | 0.071 | Review |
| **Mexico** | Mexicali | 2016 | 0.007 | Review |
| **Mexico** | Monterrey | 2016 | 0.015 | Review |
| **Mexico** | Navolato | 2016 | 0.129 | Review |
| **Mexico** | Nuevo Laredo | 2016 | 0.026 | Review |
| **Mexico** | Oaxaca | 2016 | 0.030 | Review |
| **Mexico** | Orizaba | 2016 | 0.011 | Review |
| **Mexico** | Piedras Negras | 2016 | 0.002 | Review |
| **Mexico** | Poza Rica | 2016 | 0.131 | Review |
| **Mexico** | Progreso | 2016 | 0.108 | Review |
| **Mexico** | Reynosa | 2016 | 0.042 | Review |
| **Mexico** | Salamanca | 2016 | 0.001 | Review |
| **Mexico** | San Francisco de Campeche | 2016 | 0.171 | Review |
| **Mexico** | San Gabriel Chilac | 2016 | 0.031 | Review |
| **Mexico** | San Jose del Cabo | 2016 | 0.052 | Review |
| **Mexico** | San Pedro | 2016 | 0.058 | Review |
| **Mexico** | San Pedro Pochutla | 2016 | 0.152 | Review |
| **Mexico** | Santa Catarina | 2016 | 0.013 | Review |
| **Mexico** | Tanquián De Escobedo | 2016 | 0.003 | Review |
| **Mexico** | Tapachula | 2016 | 0.274 | Review |
| **Mexico** | Tecpan de Galeana | 2016 | 0.235 | Review |
| **Mexico** | Teocaltiche | 2016 | 0.003 | Review |
| **Mexico** | Tepic | 2016 | 0.055 | Review |
| **Mexico** | Tijuana | 2016 | 0.004 | Review |
| **Mexico** | Tlapacoyan | 2016 | 0.124 | Review |
| **Mexico** | Tonala | 2016 | 0.024 | Review |
| **Mexico** | Tuxtla Gutierrez | 2016 | 0.159 | Review |
| **Mexico** | Uruapan | 2016 | 0.004 | Review |
| **Mexico** | Valle de Santiago | 2016 | 0.002 | Review |
| **Mexico** | Veracruz | 2016 | 0.130 | Review |
| **Mexico** | Xalapa | 2016 | 0.005 | Review |
| **Mexico** | Zacatepec | 2016 | 0.046 | Review |
| **Mexico** | Zamora | 2016 | 0.032 | Review |
| **Mexico** | Zapopan | 2016 | 0.005 | Review |
| **Mexico** | Zaragoza | 2016 | 0.019 | Review |
| **Mexico** | Zihuatanejo | 2016 | 0.649 | Review |
| **Mexico** | Yucatan | 2014 | 0.045 | Review |
| **Mexico** | State of Morelos | 2011 | 0.044 | Review |
| **Mexico** | Matamoros, Tamaulipas | 2004 | 0.009 | Cattarino et al 2020 |
| **Mexico** | Matamoros, Tamaulipas | 2005 | 0.009 | Cattarino et al 2020 |
| **Mexico** | Morelos | 2015* | 0.014 | Cattarino et al 2020 |
| **Namibia** | Windhoek | 2011-2012 | 0.001 | Cattarino et al 2020 |
| **Nigeria** | Osogbo, Osun State | 2014-2015 | 0.018 | Review |
| **Nigeria** | Kainji Lake | 1980 | 0.006 | Cattarino et al 2020 |
| **Pakistan** | Lahore | 2010-2015 | 0.060 | Review |
| **Pakistan** | Khyber Pakhtunkhawa | pre_2013 | 0.002 | Cattarino et al 2020 |
| **Pakistan** | Lahore | 2012 | 0.008 | Cattarino et al 2020 |
| **Pakistan** | Lahore | 2011-2016 | 0.020 | Cattarino et al 2020 |
| **Papua New Guinea** | Madang province | 2007_2008 | 0.056 | Cattarino et al 2020 |
| **Peru** | Iquitos | 2010 | 0.051 | Review |
| **Peru** | Iquitos | 1992 | 0.009 | Cattarino et al 2020 |
| **Peru** | Santa Clara, Iquitos | 1996 | 0.003 | Cattarino et al 2020 |
| **Peru** | Iquitos | 1999 | 0.032 | Cattarino et al 2020 |
| **Philippines** | Cebu | 2015* | 0.061 | Cattarino et al 2020 |
| **Reunion** | Reunion Island | 2008 | 0.001 | Cattarino et al 2020 |
| **Saudi Arabia** | Makkah | 2016-2017 | 0.021 | Review |
| **Saudi Arabia** | Madinah | 2016-2017 | 0.004 | Review |
| **Saudi Arabia** | Jeddah | 2016-2017 | 0.007 | Review |
| **Saudi Arabia** | Jizan | 2016-2017 | 0.023 | Review |
| **Saudi Arabia** | Jeddah | 2015* | 0.017 | Review |
| **Saudi Arabia** | Jeddah | pre_2016 | 0.005 | Cattarino et al 2020 |
| **Singapore** | national | 2008-2010 | 0.020 | Review |
| **Singapore** | blood service | 2009-2010 | 0.049 | Review |
| **Singapore** | national | 2010 | 0.034 | Review |
| **Singapore** | Singapore | 2010 | 0.006 | Cattarino et al 2020 |
| **Singapore** | Singapore | 1982_1984 | 0.014 | Cattarino et al 2020 |
| **Singapore** | Singapore | 2007 | 0.007 | Cattarino et al 2020 |
| **Singapore** | Singapore | 2004_2007 | 0.006 | Cattarino et al 2020 |
| **Singapore** | Singapore | 2009-2010 | 0.005 | Cattarino et al 2020 |
| **Sri Lanka** | City of Colombo | 2008-2010 | 0.149 | Review |
| **Sri Lanka** | Ramalana community in Colombo district | 2017 | 0.077) | Review |
| **Sri Lanka** | Trincomalee district | 2022-2023 | 0.065 | Review |
| **Sri Lanka** | Jaffna district | 2022-2023 | 0.039 | Review |
| **Sri Lanka** | Kurunegala district | 2022-2023 | 0.013 | Review |
| **Sri Lanka** | Matara district | 2022-2023 | 0.014 | Review |
| **Sri Lanka** | Ratnapura district | 2022-2023 | 0.046 | Review |
| **Sri Lanka** | Polonaruwa district | 2022-2023 | 0.026 | Review |
| **Sri Lanka** | Gampha district | 2022-2023 | 0.029 | Review |
| **Sri Lanka** | Kandy district | 2022-2023 | 0.015 | Review |
| **Sri Lanka** | Badulla district | 2022-2023 | 0.012 | Review |
| **Sri Lanka** | Sri Jayewadenpura | 2013-2014 | 0.068 | Review |
| **Sri Lanka** | Maharagama Medical Officer Health, Colombo | pre_2006 | 0.010 | Cattarino et al 2020 |
| **Sri Lanka** | Colombo | 2008_2009 | 0.032 | Cattarino et al 2020 |
| **Sri Lanka** | Colombo | 2008 | 0.034 | Cattarino et al 2020 |
| **Sudan** | Karima | 1989 | 0.002 | Cattarino et al 2020 |
| **Sudan** | Kassala state | 2011 | 0.001 | Cattarino et al 2020 |
| **Taiwan** | Taipei, Taoyuan, Tainan | 2010 | 0.005 | Review |
| **Taiwan** | Nanzih district, Kaohsiung City | 2015-2016 | 0.001 | Review |
| **Taiwan** | Sanmin district, Kaohsiung City | 2015-2016 | 0.001 | Review |
| **Taiwan** | Pingtung County | 1997 | 0.005 | Cattarino et al 2020 |
| **Tanzania** | Buhigwe, Kalambo, Kilindi, Kinondoni, Kondoa, Kyela, Mvomero and Ukerewe | 2018 | 0.011 | Review |
| **Tanzania** | Zanzibar | 2010 | 0.114 | Review |
| **Tanzania** | Zanzibar | 2011 | 0.007 | Cattarino et al 2020 |
| **Thailand** | Mukdahn | 2019 | 0.112 | Review |
| **Thailand** | Ubon | 2019 | 0.119 | Review |
| **Thailand** | South (Narathiwat and Trang) | 2014 | 0.070 | Review |
| **Thailand** | Central (Ayutthaya and Lop Buri) | 2014 | 0.070 | Review |
| **Thailand** | Ratchaburi province | 2012-2015 | 0.136 | Review |
| **Thailand** | Ratchaburi province | 2019-2020 | 0.094 | Review |
| **Thailand** | Bangkok | 2000 | 0.028 | Cattarino et al 2020 |
| **Thailand** | Mueang district, Ratchaburi Province | 2000 | 0.035 | Cattarino et al 2020 |
| **Thailand** | Mueang Rayong District | 2010 | 0.019 | Cattarino et al 2020 |
| **Thailand** | Rayong, Rayong Province | 1980 | 0.044 | Cattarino et al 2020 |
| **Thailand** | Ayutthaya province | 2014 | 0.021 | Cattarino et al 2020 |
| **Thailand** | Narathiwat province | 2014 | 0.071 | Cattarino et al 2020 |
| **United States** | Brownsville, Texas | 2004 | 0.003 | Cattarino et al 2020 |
| **United States** | Brownsville, Texas | 2005 | 0.003 | Cattarino et al 2020 |
| **Venezuela** | Cana de Azucar | 2010-2011 | 0.059 | Review |
| **Venezuela** | Isla de San Carlos | 1995 | 0.005 | Cattarino et al 2020 |
| **Venezuela** | Maracay | 2010 | 0.025 | Cattarino et al 2020 |
| **Vietnam** | Nha Trang City, Nih Hoa district and Dien Khanh district (in Khanh Hoa province) | 2011 | 0.012 | Review |
| **Vietnam** | Cao Lanh City, Dong Thap Province | 1996_1997 | 0.036 | Cattarino et al 2020 |
| **Vietnam** | Binh Thuan Province | pre_2005 | 0.028 | Cattarino et al 2020 |

*The date of the survey was not specified, instead we report the year of publication of the article.

**Figures**

**
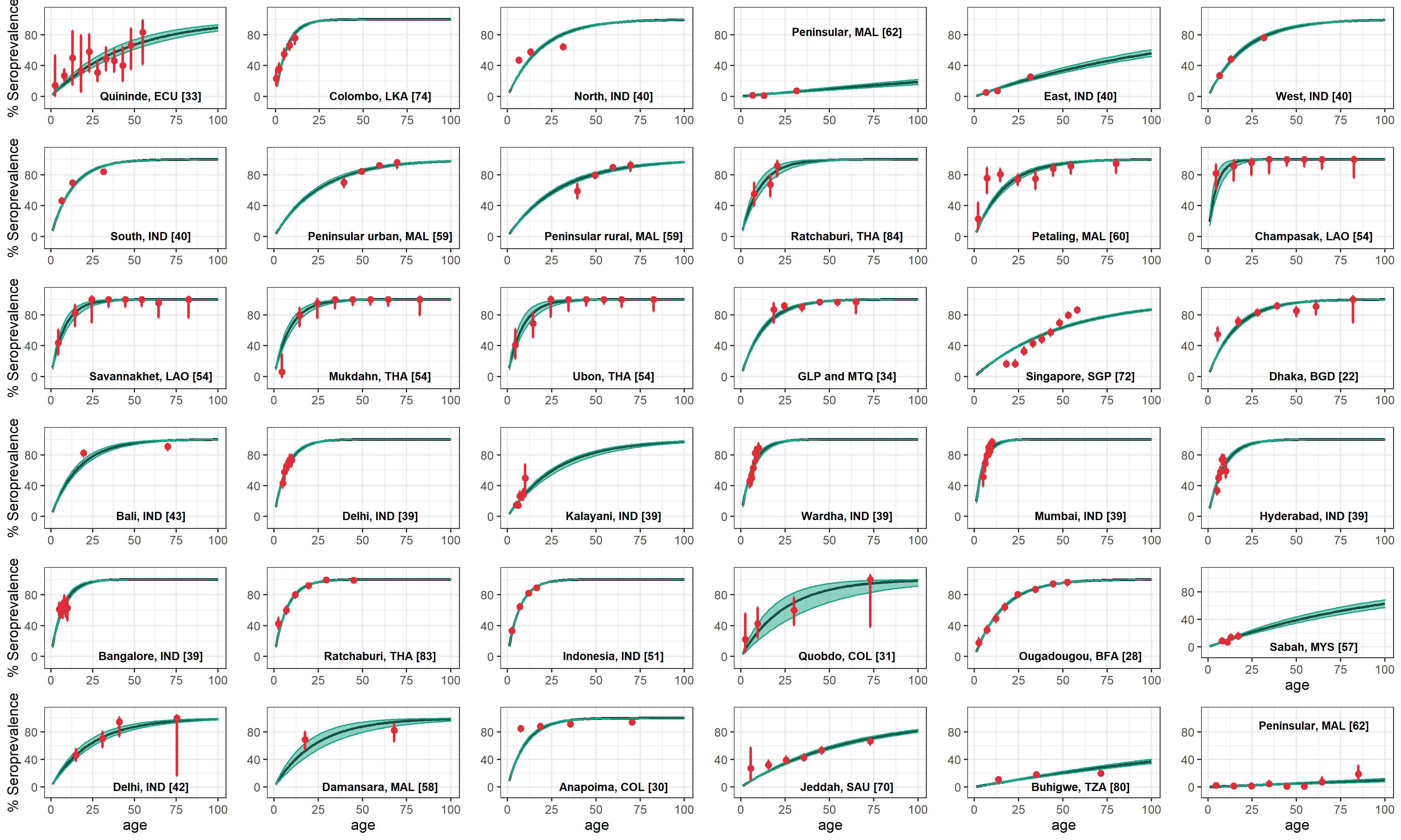
**

**Figure S1 Model fit obtained under model A1 (binomial) part 1.** The points (in red) represent all available samples with their binomial 95% confidence interval (CI) and the continuous black line and green shading represent the median and 95% credible interval (CrI) obtained from 1000 random samples of the estimated FOI from the posterior distribution.


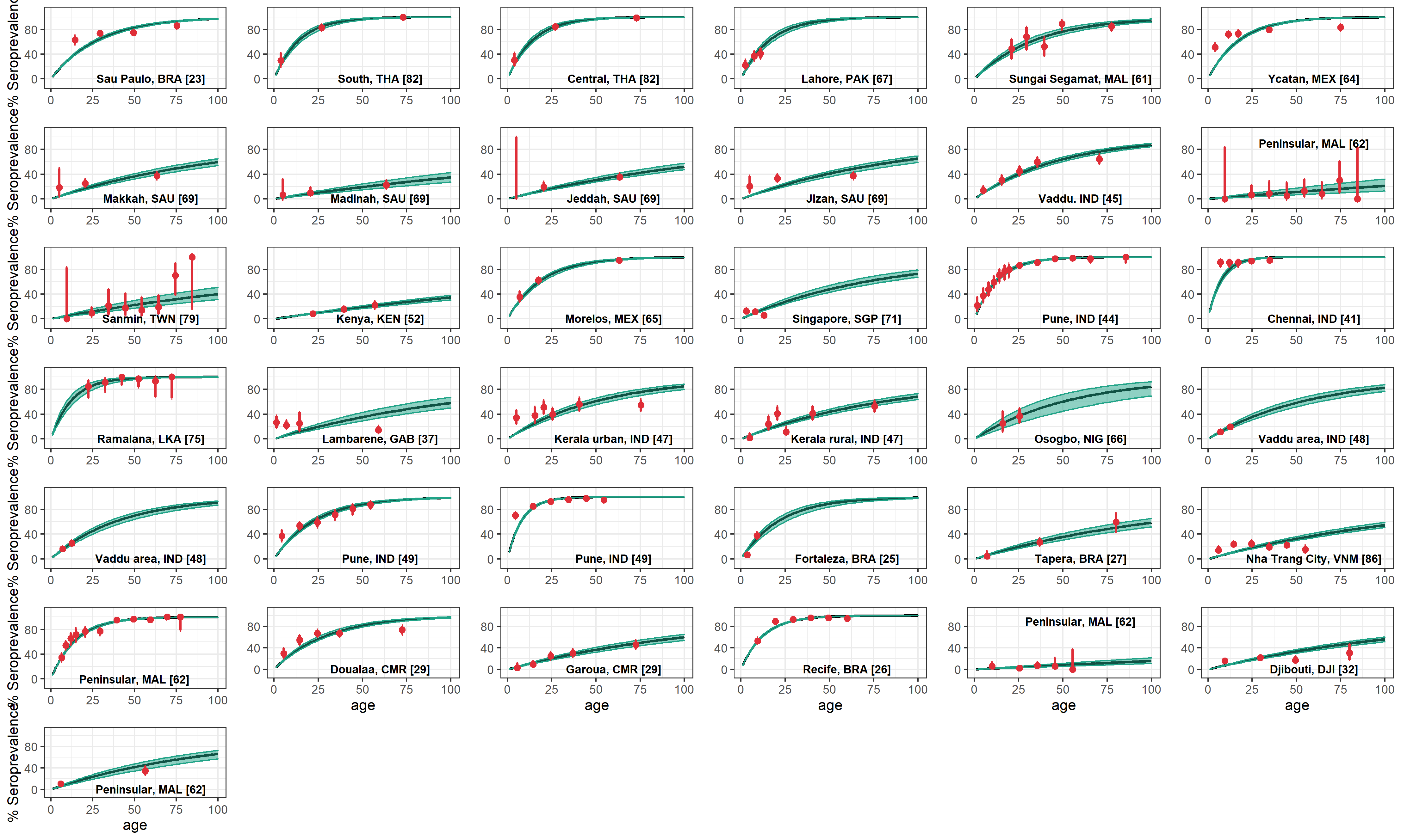


**Figure S2 Model fit obtained under model A1 (binomial) part 2.** The points (in red) represent all available samples with their binomial 95% confidence interval (CI) and the continuous black line and green shading represent the median and 95% credible interval (CrI) obtained from 1000 random samples of the estimated FOI from the posterior distribution.

**
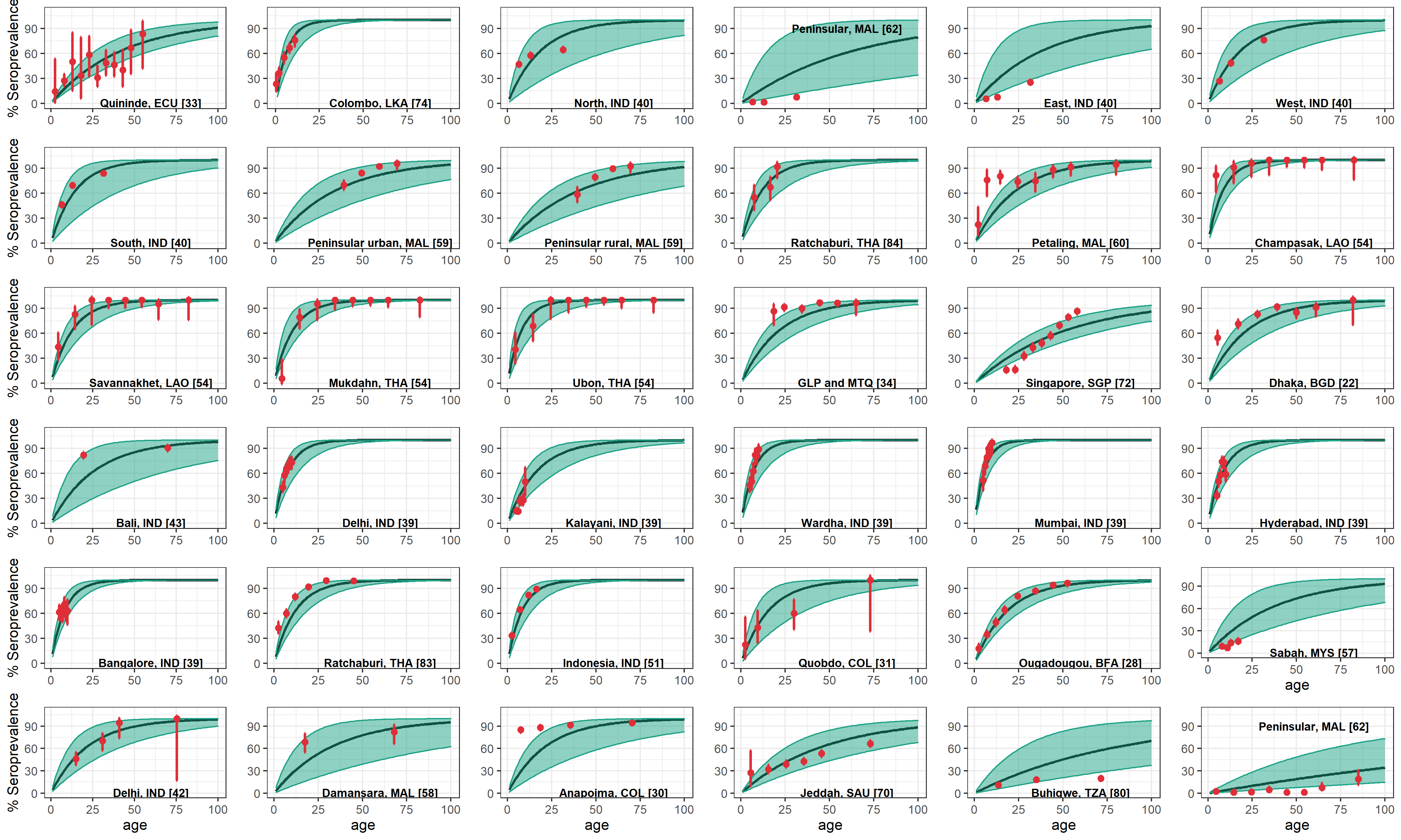
**

**Figure S3 Model fit obtained under model A2 (beta-binomial) part 1.** The points (in red) represent all available samples with their binomial 95% confidence interval (CI) and the continuous black line and green shading represent the median and 95% credible interval (CrI) obtained from 1000 random samples of the estimated FOI from the posterior distribution.

**
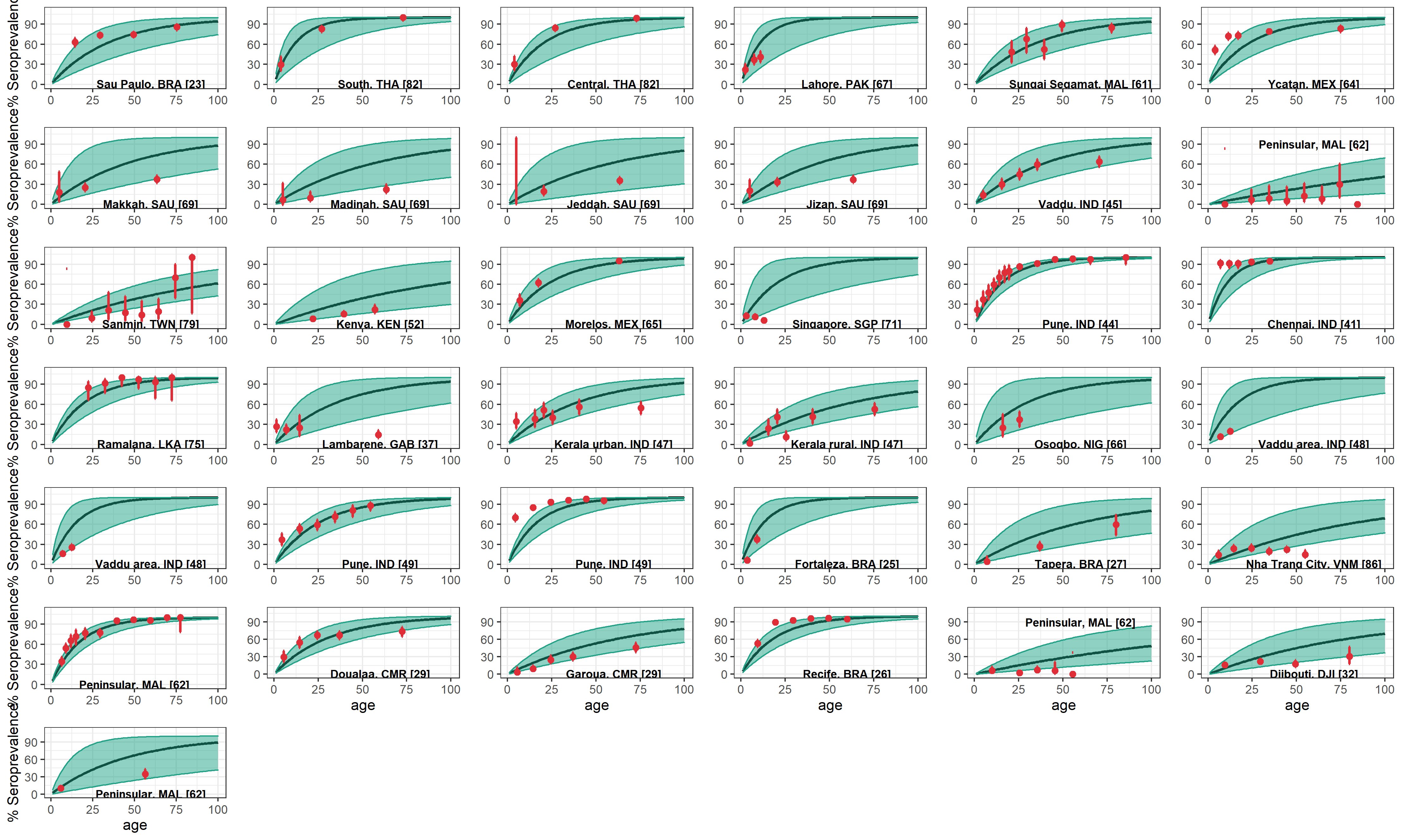
**

**Figure S4 Model fit obtained under model A2 (beta-binomial) part 2.** The points (in red) represent all available samples with their binomial 95% confidence interval (CI) and the continuous black line and green shading represent the median and 95% credible interval (CrI) obtained from 1000 random samples of the estimated FOI from the posterior distribution.


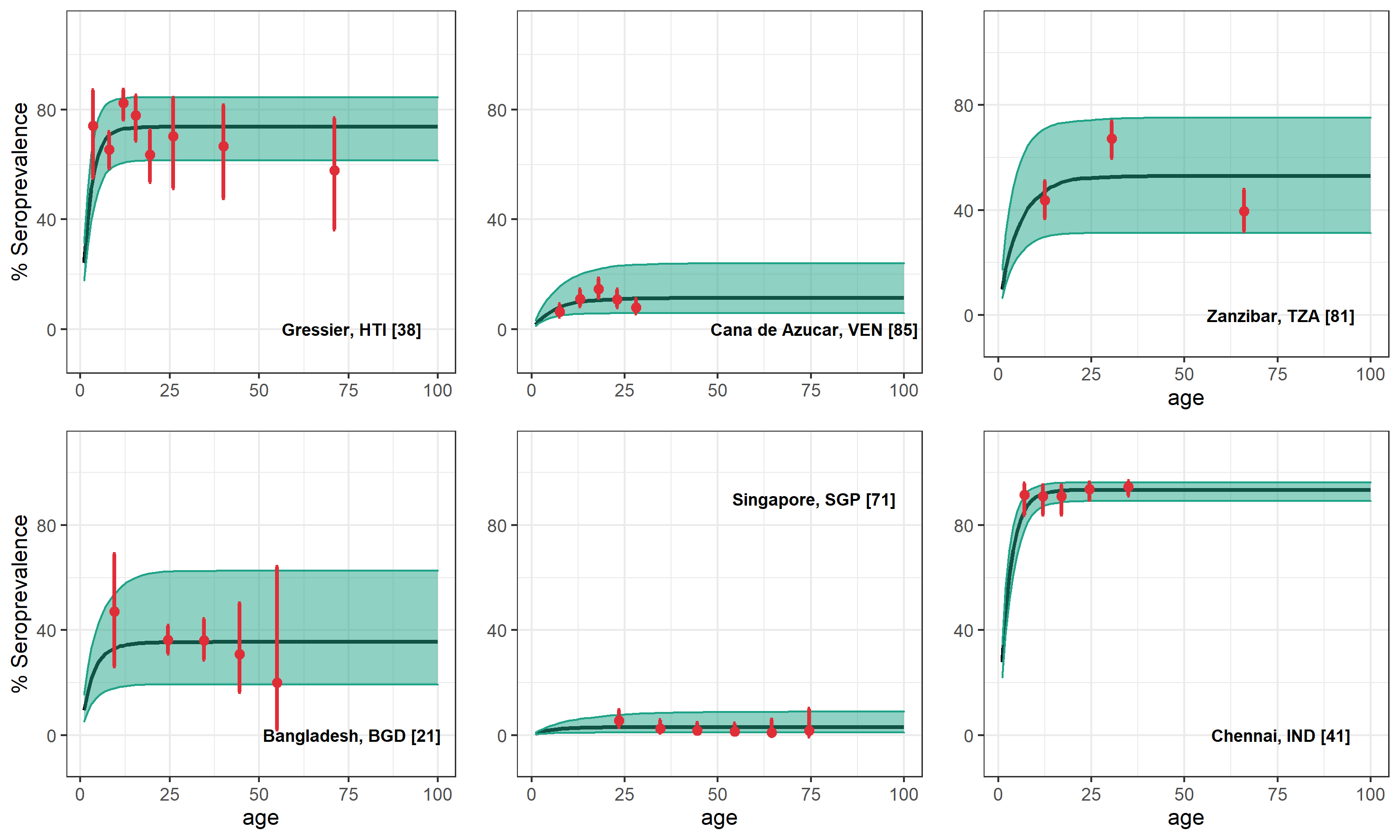


**Figure S5 Model fit obtained under model B1 (binomial).** The points (in red) represent all available samples with their binomial 95% confidence interval (CI) and the continuous black line and green shading represent the median and 95% credible interval (CrI) obtained from 1000 random samples of the estimated FOI from the posterior distribution.


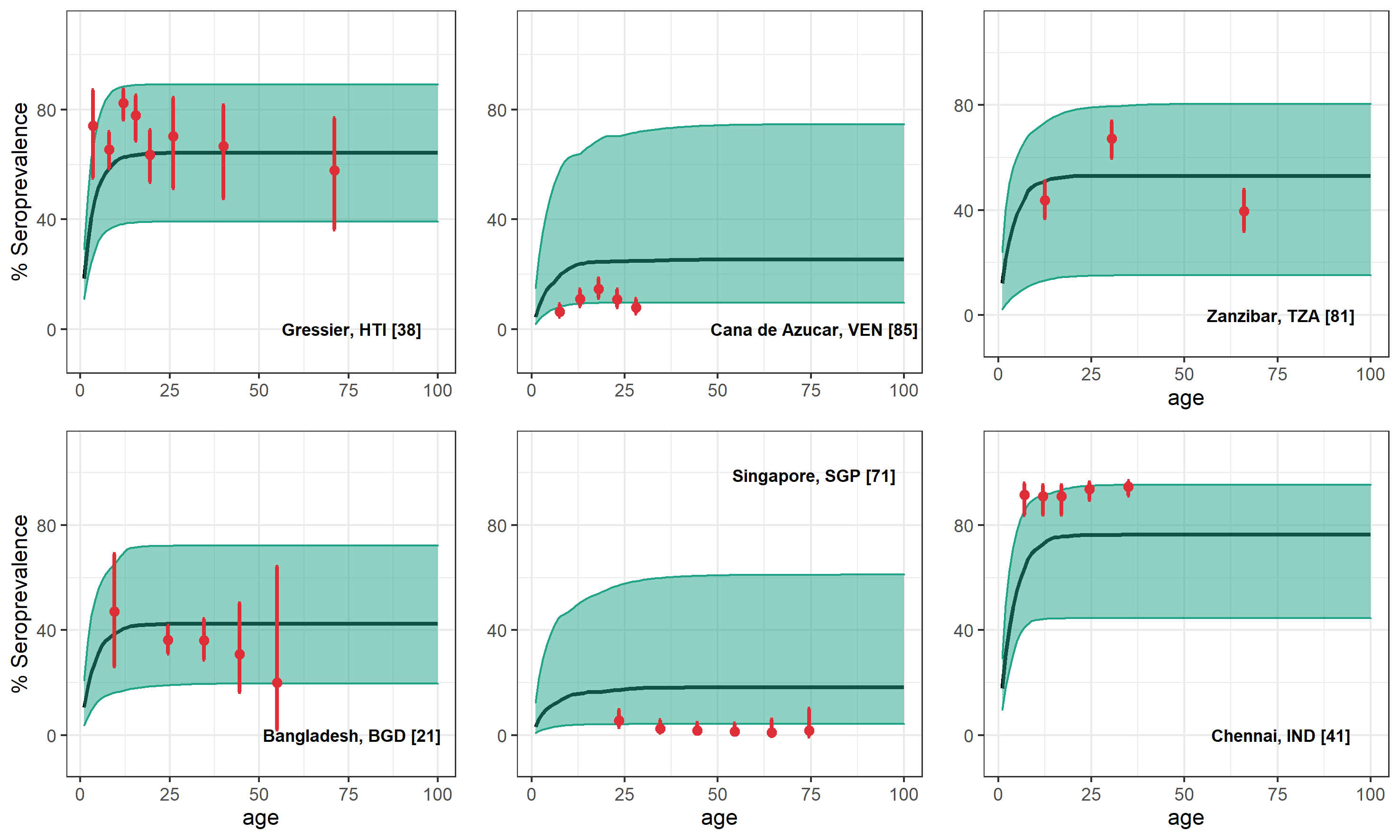


**Figure S6 Model fit obtained under model B2 (beta-binomial).** The points (in red) represent all available samples with their binomial 95% confidence interval (CI) and the continuous black line and green shading represent the median and 95% credible interval (CrI) obtained from 1000 random samples of the estimated FOI from the posterior distribution.


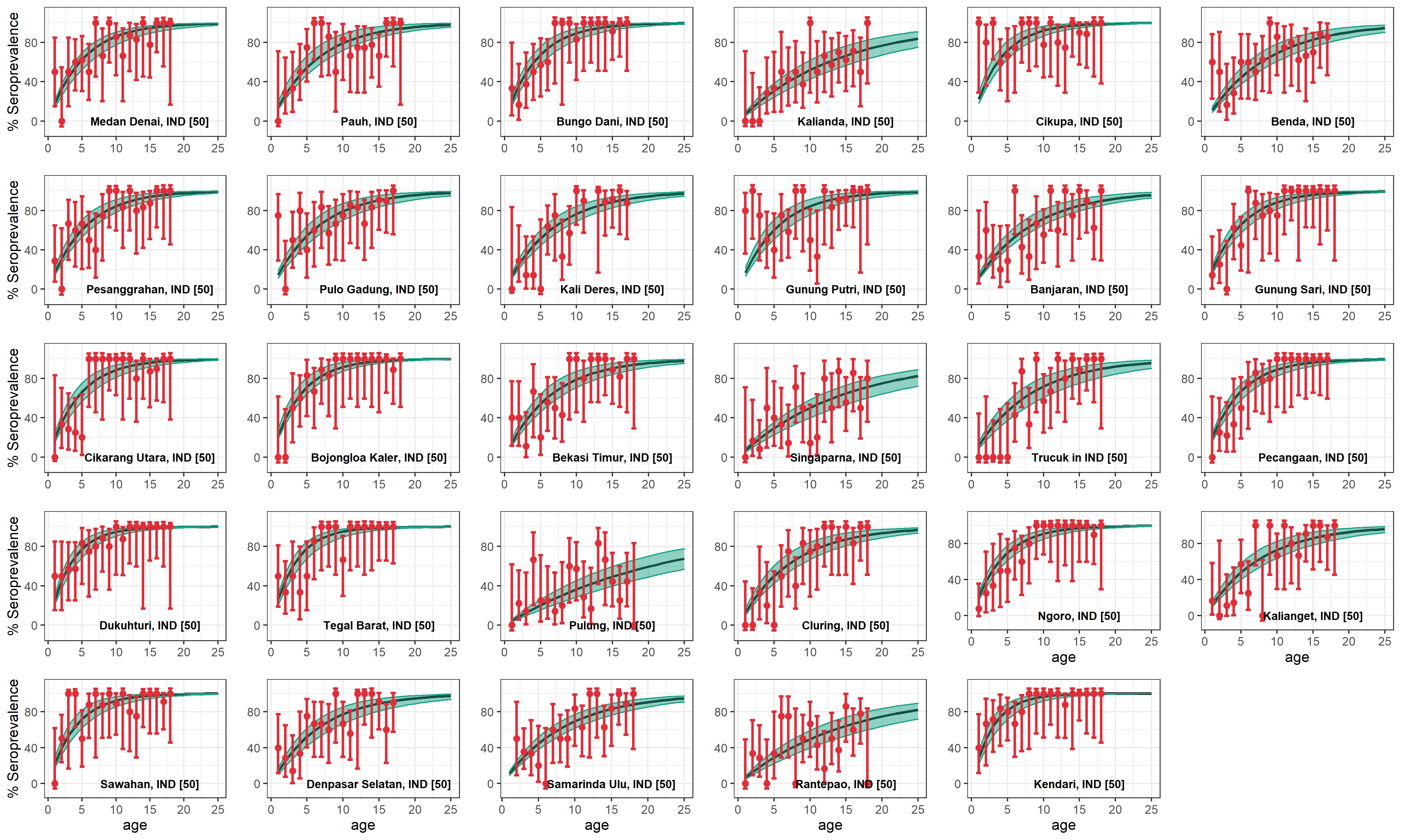


**Figure S7 Model fit obtained under model A1 (binomial) in Indonesia [50].** The points (in red) represent all available samples with their binomial 95% confidence interval (CI) and the continuous black line and green shading represent the median and 95% credible interval (CrI) obtained from 1000 random samples of the estimated FOI from the posterior distribution.


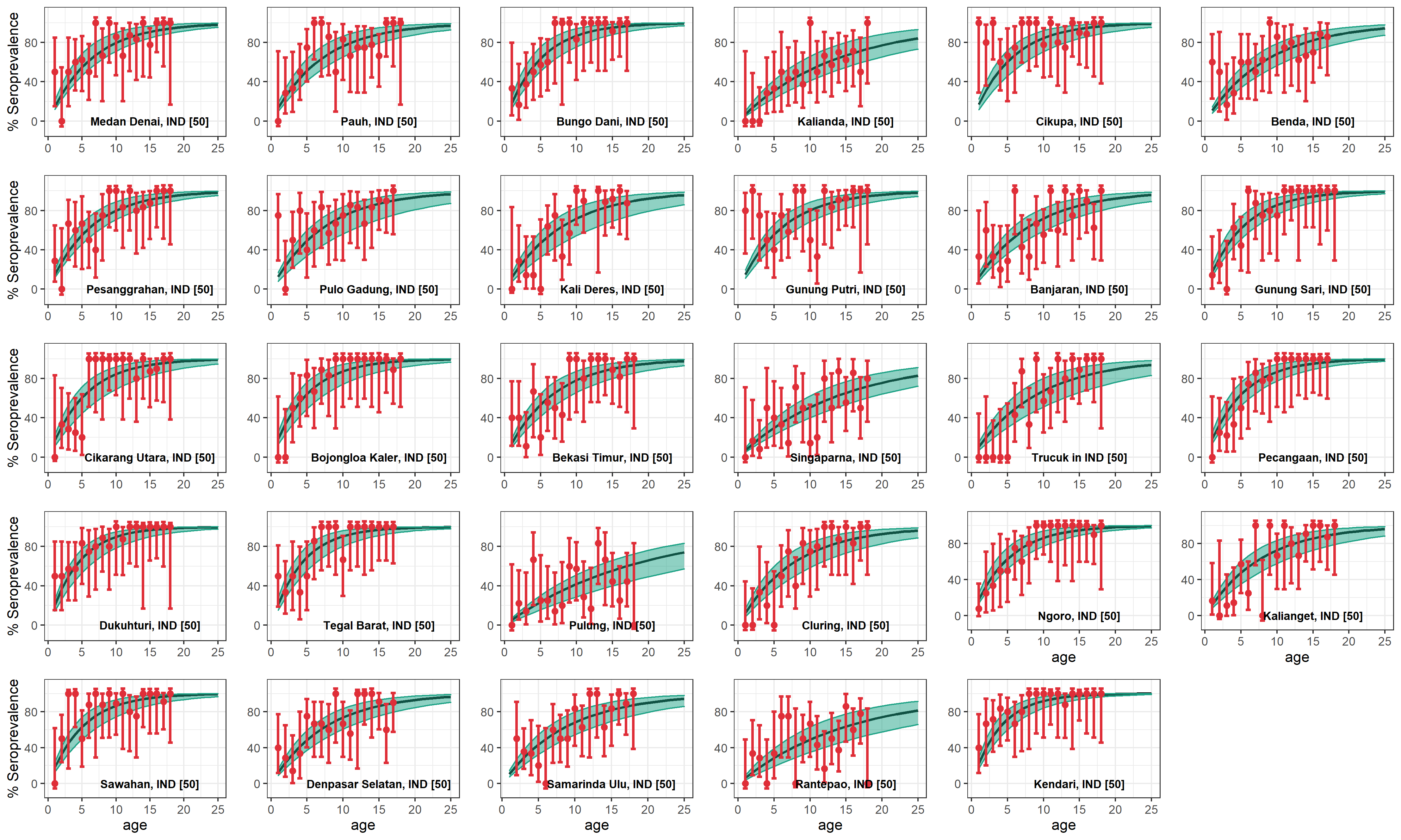


**Figure S8 Model fit obtained under model A2 (beta-binomial) in Indonesia [50].** The points (in red) represent all available samples with their binomial 95% confidence interval (CI) and the continuous black line and green shading represent the median and 95% credible interval (CrI) obtained from 1000 random samples of the estimated FOI from the posterior distribution.


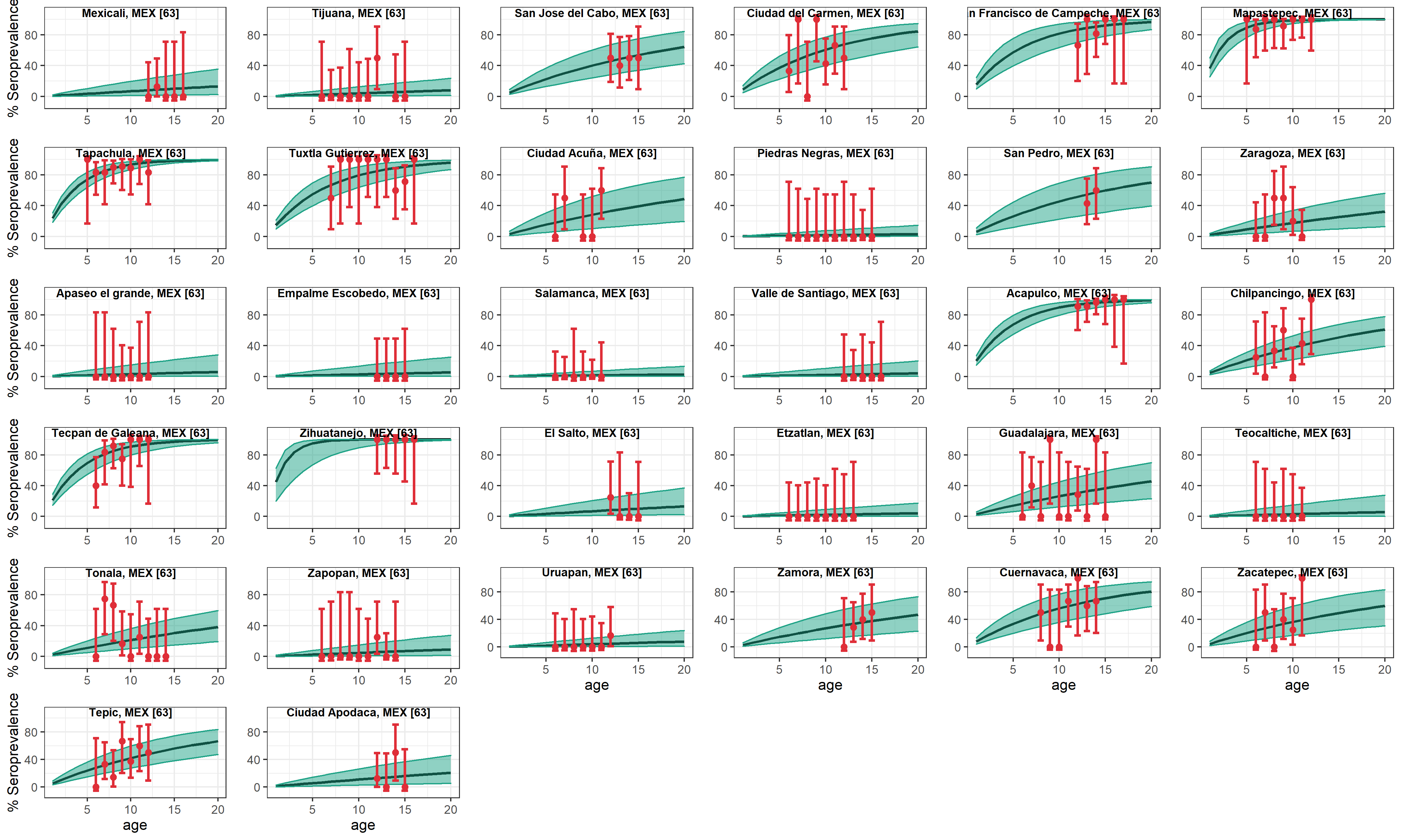


**Figure S9 Model fit obtained under model A1 (binomial) in Mexico part 1 [63].** The points (in red) represent all available samples with their binomial 95% confidence interval (CI) and the continuous black line and green shading represent the median and 95% credible interval (CrI) obtained from 1000 random samples of the estimated FOI from the posterior distribution.


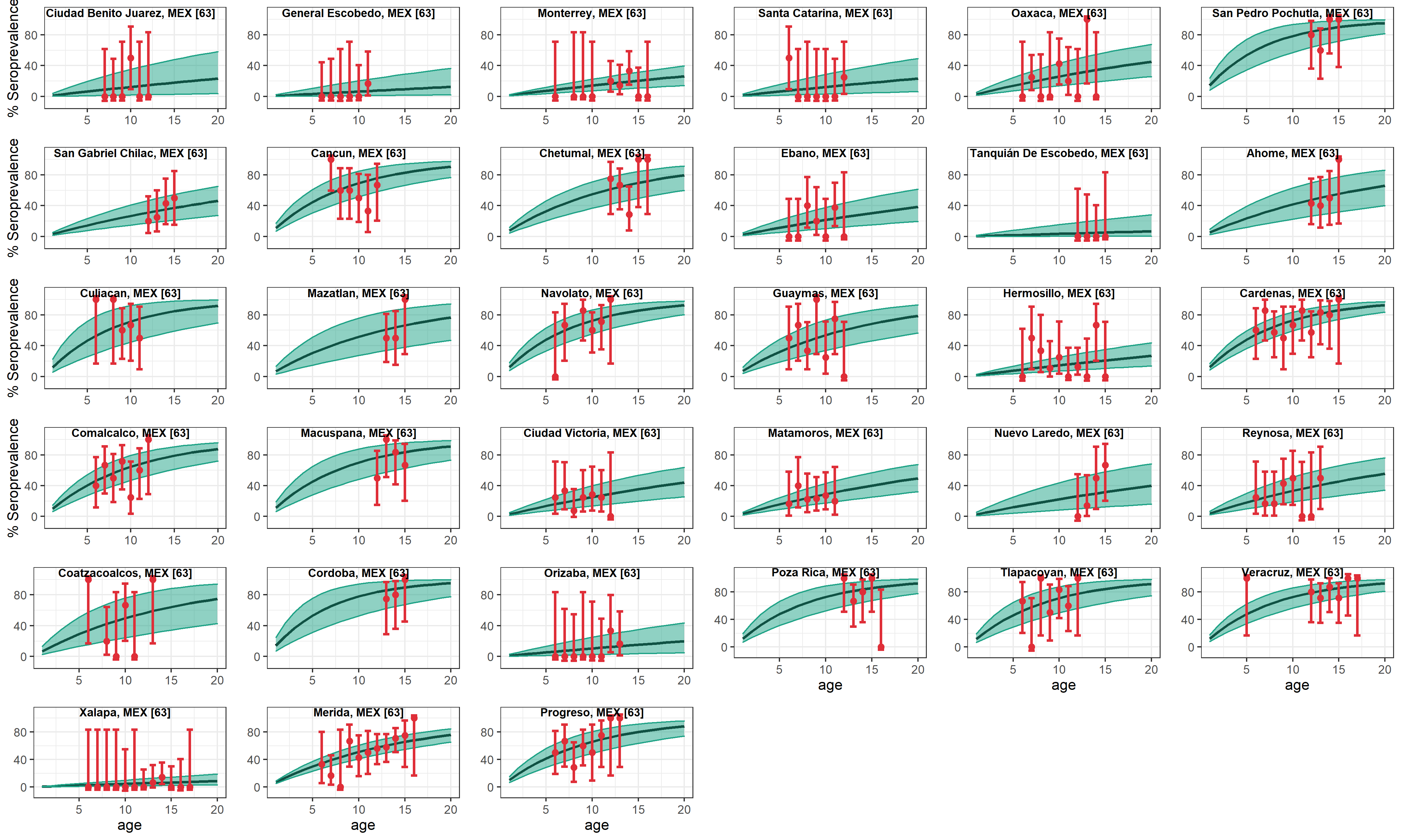


**Figure S10 Model fit obtained under model A1 (binomial) in Mexico part2 [63].** The points (in red) represent all available samples with their binomial 95% confidence interval (CI) and the continuous black line and green shading represent the median and 95% credible interval (CrI) obtained from 1000 random samples of the estimated FOI from the posterior distribution.


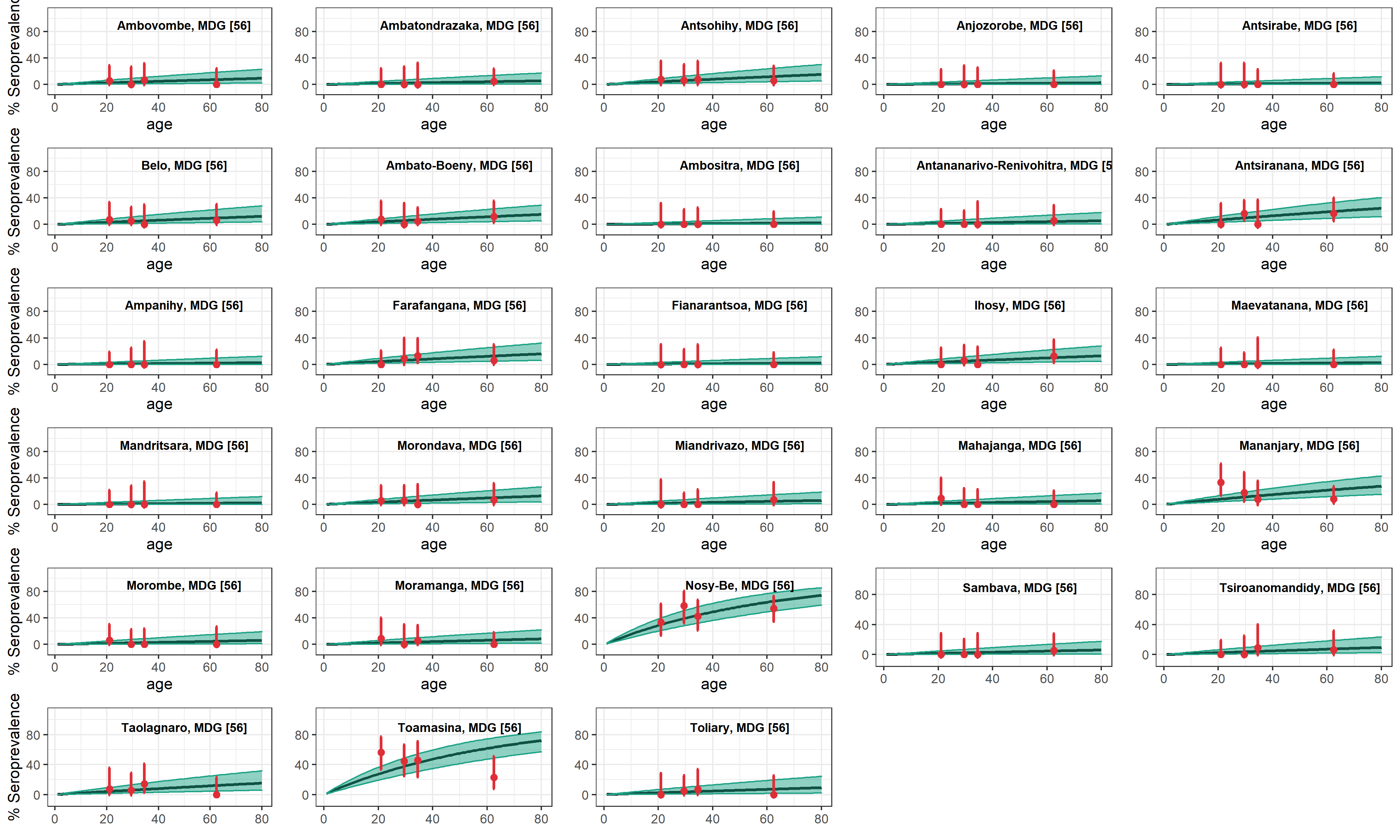


**Figure S11 Model fit obtained under model A1 (binomial) in Madagascar [56].** The points (in red) represent all available samples with their binomial 95% confidence interval (CI) and the continuous black line and green shading represent the median and 95% credible interval (CrI) obtained from 1000 random samples of the estimated FOI from the posterior distribution.

**
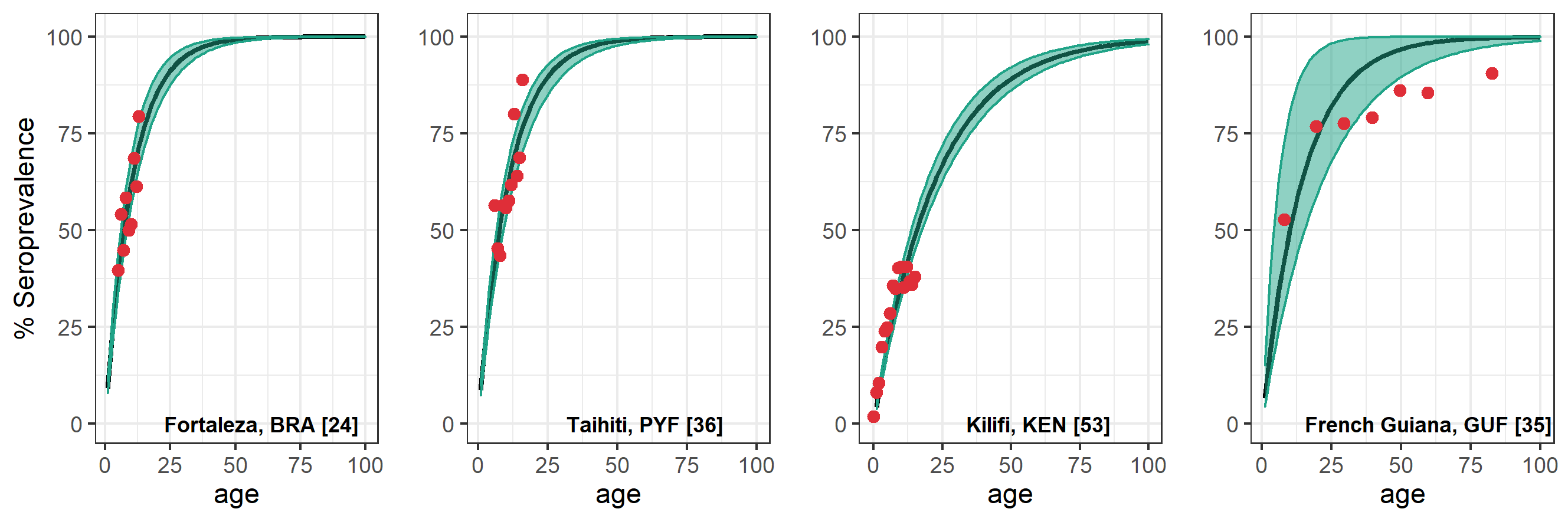
**

**Figure S12 Model fit obtained under model C (normal).** The points (in red) represent all available samples with their binomial 95% confidence interval (CI) and the continuous black line and green shading represent the median and 95% credible interval (CrI) obtained from 1000 random samples of the estimated FOI from the posterior distribution.


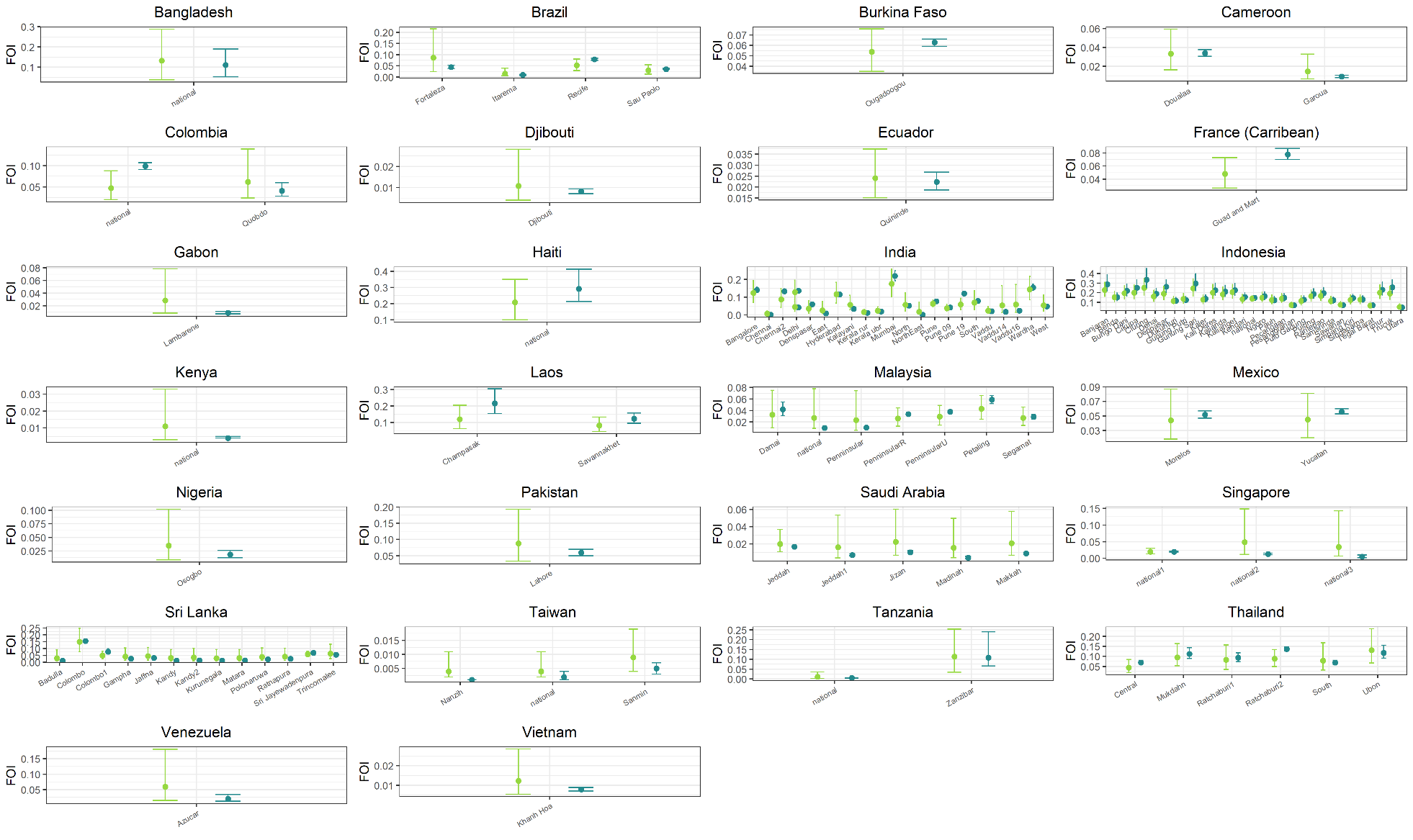


**Figure S13 FOI estimates obtained under models A1, A2, B1, B2 and C for each dataset across several countries.** The x axis reports the location of the serological survey. Each FOI estimate is reported as median and 95% credible interval (CrI). The results from the model A1 and B1 (binomial likelihood) are reported in light blue and compared with those from model A2 and B2 (beta-binomial likelihood) highlighted in green.
